# Improving genomic prediction accuracy of complex traits by integrating massive types of functional annotation information

**DOI:** 10.1038/s41467-026-72470-0

**Published:** 2026-04-24

**Authors:** Zhenshuang Tang, Xiong Xiong, Haohao Zhang, Dong Yin, Yuhua Fu, Yunxia Zhao, Jingjin Li, Yuan Quan, Xiang Zhou, Xinyun Li, Lilin Yin, Shuhong Zhao, Xiaolei Liu

**Affiliations:** 1 Yazhouwan National Laboratory, Sanya, China; 2 College of Animal Science and Technology, Huazhong Agricultural University, Wuhan, China; 3 Hubei Hongshan Laboratory, Wuhan, China; 4 School of Computer Science and Technology, Wuhan University of Technology, Wuhan, China; 5 Agricultural Animal Genetics, Breeding and Reproduction, Ministry of Education, Wuhan, China; 6 Frontiers Science Center for Animal Breeding and Sustainable Production, Ministry of Education, Wuhan, China; 7 College of Informatics, Huazhong Agricultural University, Wuhan, China; 8 Hubei Key Laboratory of Agricultural Bioinformatics, Huazhong Agricultural University, Wuhan, China; 9 Department of Biostatistics, University of Michigan, Ann Arbor, MI USA; 10 Center for Statistical Genetics, University of Michigan, Ann Arbor, MI USA; 11 Key Laboratory of Swine Genetics and Breeding, Ministry of Agriculture and Rural Affairs, Wuhan, China

**Keywords:** Functional genomics, Data integration, Genomics

## Abstract

The development of omics techniques has increased the number of functional genomic annotations, making it possible to leverage this information in statistical models to improve genomic prediction performance. However, it remains challenging to effectively use the variety of functional annotations available. Here, we develop an adaptive model we call IFAM, which extends the linear mixed model with multiple random effects to accommodate a greater number of types of functional annotations to improve prediction accuracy for complex traits. The IFAM yields improvements in prediction accuracy across diverse datasets compared to the GBLUP model, achieving an average improvement of 9.46%, 6.15%, 4.59%, 4.09%, and 10.83% in the WTCCC1, UK Biobank, Duroc pig, Yorkshire pig and rice datasets, respectively. Notably, excluding trait-specific GWAS-derived annotations, functional annotations alone yielded a 1.40% predictive gain for the UK Biobank dataset but only marginal improvements in livestock and crop datasets. This suggests that the effectiveness of multi-omics information is constrained by species-specific annotation quality, tissue and trait relevance, and the limited resolution of existing functional datasets. As functional genomics resources continue to expand, their integration is expected to provide increasing benefits for genomic prediction.

## Abstract (web summary)

Functional genomics annotations can be used to improve genomic predictions, but it is challenging to do so effectively. Here, the authors develop an adaptive model called IFAM to address this problem and validate it in five datasets.

## Introduction

Genomic prediction plays an important role in plant and animal breeding, as well as in human precision medicine^1–3^. The genomic prediction relies on linkage disequilibrium (LD) between single-nucleotide polymorphisms (SNPs) and their nearby quantitative trait nucleotides (QTNs, also known as causal variants) to predict objective traits. A variety of genomic prediction models have been proposed, including methods under the Best Linear Unbiased Prediction (BLUP) framework, e.g., genomic BLUP (GBLUP)^4^, Single-step genomic BLUP^5^, and MultiBLUP^6^; and the family of methods constituting the “Bayesian alphabet”, such as BayesA^3^, BayesC$\pi$^7^, and BayesR^8^. In particular, the MultiBLUP and BayesR are representative methods among them, the former allowed a mixture of normal distribution of SNP effects by extending the BLUP to include multiple random effects^6^, while the latter assumed four distinct classes (zero, small, medium, and large) for SNP effects^8^. The flexible assumptions on the distribution of SNP effects allow them to capture the genetic architecture of complex traits, making it more accurate than other methods.

Studies have revealed that the SNPs explain approximately 5%–49% of the phenotypic variance of complex traits, and the “missing heritability” is widespread in genetic evaluation^9,10^. The omics data, as a bridge between genetic variants and phenotype, could potentially solve it to a certain extent^11–13^. Incorporating omics data of related processes, such as the transcription and translation of genes into the prediction model, not only enhances the dissection of complex traits, thereby improving the accuracy of prediction, but also endows the results with stronger biological relevance^14,15^. The functional annotations of the genome refer to the process of mapping genomic variants to their biological roles, and their data are more readily understood, obtained, and utilized. Therefore, integrating functional annotations into genomic prediction models can offer a promising strategy for improving prediction accuracy and conducting omics-based genetic evaluation and breeding.

Some methods, such as BayesRC^16^, AnnoPred^17^, and LDpred-funct^18^, leverage the genomic annotations as prior information to improve genomic prediction. MacLeod et al.^16^ proposed the BayesRC model to improve the accuracy of prediction by incorporating prior biological information into the BayesR model. It works by dividing the genome into different annotation categories and then assigning different prior probabilities to the genetic variants within each category. But it is not suitable for the case that one SNP belongs to multiple annotations and its computational complexity is pretty high due to the huge number of MCMC iterations. Recently, Zheng et al.^19^ extended the BayesRC model to handle summary-level data, called SBayesRC, which refines signals from functional annotations by affecting both causal variant probability and causal effect distribution. Compared to other models, the SBayesRC achieved 5.28% improvements on prediction accuracy for 28 independent traits of the UK Biobank (UKB) dataset. Although a variety of advanced models, especially those based on summary statistics, have been proposed and have achieved substantial progress in leveraging functional annotations for predicting human traits or diseases, few published studies have validated or investigated the applicability of summary-level models in plant or animal breeding. This is primarily because these summary-level models rely on population-universal and chromosome-wide LD matrices derived from common reference panels (e.g., the 1000 Genomes Project)^20^. However, such reference panels are impractical for plant or animal populations due to substantial genetic diversity across breeds or strains. Moreover, the LD structure within and among chromosomes in plants and animals is considerably more complex than that observed in humans^21^. Ignoring inter-chromosomal LD and forcibly applying summary-level models to predict traits in these non-human species would lead to suboptimal or unexpected performance. Therefore, it is crucial to develop an efficient and effective model that can utilize individual-level genotypic and phenotypic data while maximizing and automating the processing of multiple functional annotations ranging from several to dozens or even hundreds to enhance genomic prediction.

In this work, we propose a model named IFAM, which is an abbreviation for “Integrating Functional Annotations by Multi random effect model”, to achieve accurate and fast genomic prediction. For the aspect of accuracy, IFAM extends the GBLUP model with multiple random effects to accommodate massive types of functional annotations and the functional annotation types with similar contributions to phenotypic variance are automatically merged into one single random effect. For the aspect of speed, IFAM efficiently handles UKB scale datasets by using our previously proposed the phenotypic variance-covariance ${\bf{V}}$ matrix-based “HE + PCG” strategy, which is pretty friendly to multiple random effect model since its computational complexity remains unchanged with the number of random effects increased^22,23^. Compared to GBLUP model, IFAM achieved an average improvement of 9.46%, 6.15%, 4.59%, 4.09%, and 10.83% in the WTCCC1, UKB, Duroc pig, Yorkshire pig, and rice datasets, respectively. The results highlight the great potential of IFAM in enhancing genomic prediction through the utilization of functional annotations for various traits across species.

## Results

The importance of functional annotations was assessed first. Subsequently, the performance of IFAM was evaluated across five diverse datasets spanning human, animal, and plant species. A comprehensive benchmark test was conducted using the WTCCC1 dataset. The scalability of IFAM on big data was assessed using the UKB dataset. For animal breeding applications, IFAM was evaluated in terms of growth traits for both Duroc and Yorkshire pig populations. Additionally, its performance on yield-related traits of rice was assessed using the data of the 3000 Rice Genome Project.

### The importance of functional annotations

**Fig. 1:**
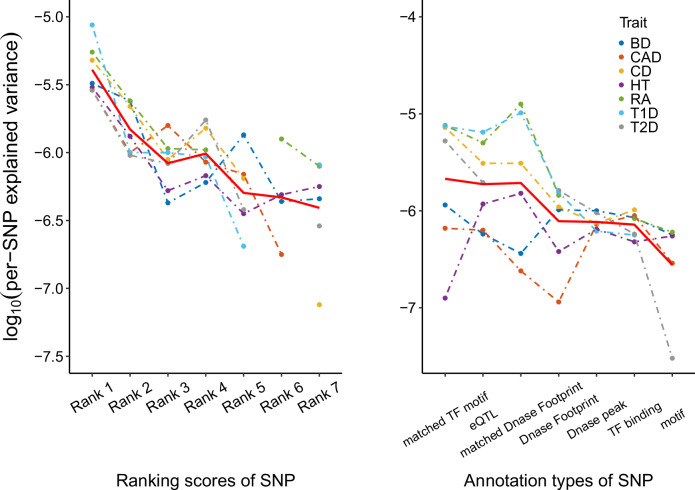
Examples of the importance assessment of functional annotations. The log10 scale of the per-SNP explained variance (*y*-axis) was estimated from the non-auto-merged multiple random effect model for ranking scores of SNP (left) and annotation types of SNP (right), which are from the RegulomeDB database. The data are from the WTCCC1 dataset, which consists of seven binary disease traits, namely bipolar disorder (BD), coronary artery disease (CAD), Crohn’s disease (CD), hypertension (HT), rheumatoid arthritis (RA), type 1 diabetes (T1D), and type 2 diabetes (T2D). The average information restricted maximum likelihood (AI-REML) algorithm was chosen to estimate the variance components. The dotted lines of different colors represent the values of different traits, and the solid red line is the mean value of all traits. Supplementary Tables 1 and2 for more details.

To determine whether functional annotations enhance genomic prediction for complex traits, we first assessed the importance of functional annotations upon the WTCCC1 and UKB datasets using SNP ranking scores from the RegulomeDB database. The results demonstrated a consistent trend where the estimated per-SNP explained variance and heritability progressively decreased with higher ranking scores (Fig. 1 and Supplementary Figs. 1 and 2), which indicating that the annotation information can server as a stable and reliable prior knowledge or guidance for SNP clustering on the genetic contribution of complex traits, i.e., variants located in genomic regions with a higher abundance of functional annotation evidence exhibited a greater average explained variance and heritability for target traits. Besides, the functional annotations such as matched TF motif, eQTL, and match DNase footprint also demonstrated a similar conclusion of the usefulness of functional annotations (Fig. 1). However, the ranking of SNPs based on functional annotations is inherently subjective, its stability and accuracy may vary across species. Therefore, we directly incorporated functional annotations into the downstream model comparisons across species.

### WTCCC1 dataset

**Fig. 2:**
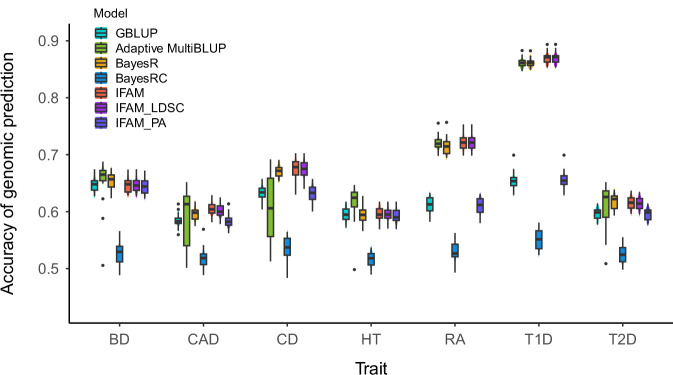
Prediction performance of different models in the WTCCC1 dataset. The IFAM represents running the IFAM model using trait-specific significant SNPs generated from GWAS and functional annotations derived from the RegulomeDB database. The IFAM_LDSC represents running the IFAM model using trait-specific significant SNPs generated from GWAS and functional annotations derived from the linkage disequilibrium score regression (LDSC) model. The IFAM_PA represents running the IFAM model using pseudo annotation information, which is selected randomly based on the scale of true annotations from the RegulomeDB database. The WTCCC1 dataset consists of seven binary disease traits, namely bipolar disorder (BD), coronary artery disease (CAD), Crohn’s disease (CD), hypertension (HT), rheumatoid arthritis (RA), type 1 diabetes (T1D), and type 2 diabetes (T2D). The accuracy of genomic prediction was assessed using the area under the curve (AUC) between estimated genetic values and phenotypic values using 20 repetitions. In all box plots, the center line represents the median, the bounds of the box represent the 25th and 75th percentiles, and the whiskers represent 1.5× the interquartile range, with outliers plotted as individual points. See the Supplementary Table 3 for more details.

The Fig. 2 presents the prediction accuracy of GBLUP, Adaptive MultiBLUP, BayesR, and BayesRC across 7 human diseases on the WTCCC1 dataset. Overall, except for the BayesRC, GBLUP showed the lowest average accuracy, despite performing relatively well for the polygenic traits such as BD and HT. Adaptive MultiBLUP and BayesR achieved comparable performance, particularly for traits with the strongest marginal associations, but adaptive MultiBLUP exhibited substantial instability across replicates, especially for CAD and CD. For our IFAM model, we first ran it using functional annotations derived from the RegulomeDB database and the trait-specific significant SNPs generated from GWAS. The peaks of DNase, transcription factor (TF) binding sites, and motifs are the top three annotation types by coverage (Supplementary Fig. 3). Incorporating these annotations, IFAM improved prediction accuracy by 9.46% on average compared to GBLUP, with a particularly notable increase of 32.98% for T1D, while maintaining comparable prediction bias (Supplementary Table 4). Against adaptive MultiBLUP, IFAM achieved an accuracy gain of 1.48% and a prediction bias closer to 1, with a 10.74% improvement for CD. IFAM also slightly outperformed BayesR, with clearer advantages for CAD and T1D. For the BayesRC, we applied the same functional annotations as those used in IFAM; nevertheless, it yielded the lowest prediction accuracy, which was unexpected (Fig. 2). When we restricted the analysis to a curated subset of highly trait-relevant annotations (e.g., eQTL and matched TF motif), the prediction accuracy of BayesRC improved substantially (0.5290 vs. 0.6030), particularly for the T1D trait (Supplementary Table 5). This sensitivity underscored BayesRC’s heavy reliance on pre-processed, high-quality annotations; in contrast, the IFAM model exhibited greater robustness when integrated with larger, more heterogeneous annotations. Compared to the non-auto-merged multiple random effect model (Supplementary Tables 3 and 4), IFAM showed comparable accuracy while consistently yielding superior prediction bias. Notably, IFAM demonstrated pronounced improvements for traits with strong genetic signals such as T1D and RA.

To investigate the impact of annotation quantity and quality, we further ran IFAM using 74 annotation types from the LDSC model^24^. The genomic coverage of functional annotations was detailed in Supplementary Data 1. Performance of IFAM with annotations from LDSC was slightly lower than that from RegulomeDB (Fig. 2), a difference likely driven by the large number and high degree of overlap among annotations from LDSC (Supplementary Fig. 4), which consequently led to suboptimal variance component estimates (Supplementary Data 2). Crucially, the non-auto-merged multiple random effect model using the annotations from LDSC showed a 5.33% decrease in accuracy and a substantial drop in prediction bias (to 0.54) compared to IFAM (Supplementary Tables 3 and 4), underscoring the necessity of the merging strategy implemented in IFAM. This also confirmed that the performance of IFAM depends on the relevance between annotations and target trait than on the sheer quantity of annotations.

Furthermore, we generated pseudo annotations by randomly selecting SNPs that matched the true annotations from the RegulomeDB database in terms of count number, the MAF and LD distribution (Supplementary Figs. 5 and 6). Using pseudo annotations significantly reduced the accuracy of IFAM by 8.79% compared to the true annotations (Fig. 2), confirming its sensitivity to biologically meaningful information. Notably, IFAM with pseudo annotations performed only 0.16% worse than GBLUP, demonstrating the strong robustness of IFAM against uninformative annotations. Overall, IFAM effectively balances predictive accuracy with computational efficiency. Its accuracy is on par with that of BayesR, while its stability surpasses that of Adaptive MultiBLUP. It stands as one of the optimal models for integrating functional annotations into genomic prediction.

### UK Biobank dataset

**Fig. 3:**
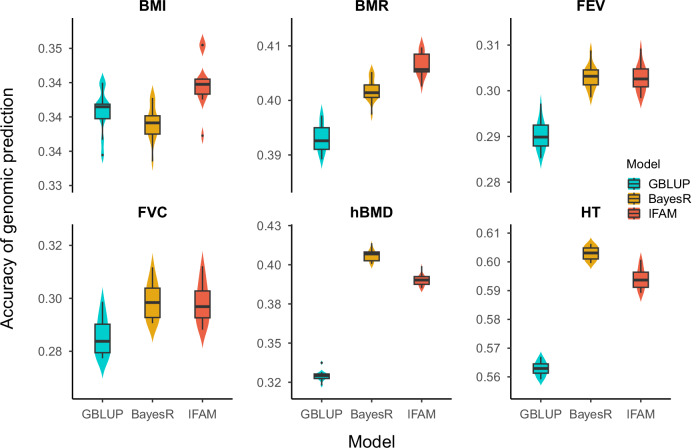
Prediction performance of different models in the UK Biobank dataset. The IFAM represents running the IFAM model using trait-specific significant SNPs generated from GWAS and functional annotations derived from the RegulomeDB database. The UKB dataset includes six traits, namely height (HT), basal metabolic rate (BMR), heel bone mineral density T-score (hBMD), forced vital capacity (FVC), body mass index (BMI), and forced expiratory volume in 1s (FEV). The accuracy of genomic prediction was assessed by the correlation between estimated genetic values and phenotypic values using 10 repetitions. In all box plots, the center line represents the median, the bounds of the box represent the 25th and 75th percentiles, and the whiskers represent 1.5× the interquartile range, with outliers plotted as individual points. See the Supplementary Table 6 for more details.

Due to the large scale of the UKB dataset, it is infeasible to run Adaptive MultiBLUP and BayesRC. Therefore, a benchmark test was conducted among GBLUP, BayesR, and IFAM (Fig. 3). IFAM was performed using functional annotations derived from the RegulomeDB database and trait-specific significant SNPs generated from GWAS. Genomic coverage by these annotations was similar to that observed in the WTCCC1 dataset (Supplementary Fig. 3). Among the three models, GBLUP yielded the lowest prediction accuracy. Compared to GBLUP, BayesR and IFAM improved average accuracy by 6.84% and 6.16%, respectively. While BayesR achieved marginally higher overall accuracy (an average difference of 0.0025), IFAM consistently demonstrated superior prediction bias (Supplementary Table 7). IFAM exhibited a distinct advantage for BMR and BMI, whereas its performance for FVC and FEV was comparable to BayesR. Moreover, IFAM substantially outperformed the non-auto-merged multiple random effect model, increasing average accuracy by 43.25% and producing a prediction bias much closer to 1 (Supplementary Tables 6 and 7). The performance of non-auto-merged multiple random model was likely compromised by suboptimal variance component estimates, a consequence of the high number of random effects and substantial overlap among annotations (Supplementary Table 8 and Supplementary Fig. 4). This result also underscored the critical importance of the automated merging strategy of IFAM in ensuring robust estimation and stable prediction performance.

As is well known, estimating variance components remains a primary computational bottleneck in big data analyses. To address this challenge, IFAM adopted an efficient “HE + PCG” strategy based on our previous work, which utilized the Haseman-Elston (HE) regression for variance component estimation and the Pre-conditioned Conjugate Gradient (PCG) iterative algorithm for genetic value prediction, thereby completely avoiding inverting any of the large matrices^22,23^. We assessed the computational scalability of IFAM using the hBMD trait from the UKB dataset, varying the number of random effects from 1 to 5. The results indicated that IFAM completed the analyses within 1.5 h, with runtime increasing only marginally alongside the number of random effects (Supplementary Fig. 7). In contrast, BayesR required over one week to complete the same task. This demonstrates that IFAM is computationally feasible for datasets comprising several hundred thousand individuals.

### Duroc pig dataset

**Fig. 4:**
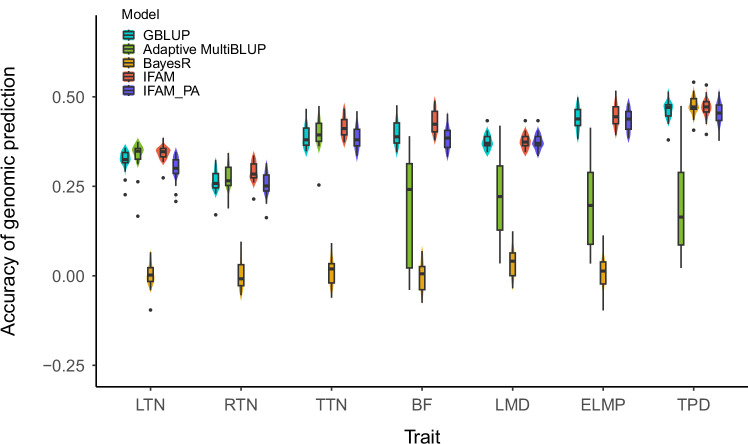
Prediction performance of different models in the Duroc pig dataset. The IFAM represents running the IFAM model using trait-specific significant SNPs generated from GWAS and functional annotations derived from the IFmut database. The IFAM_PA represents running the IFAM model using pseudo annotation information, which is selected randomly based on the scale of true annotations from the IFmut database. The data comprises the seven traits, namely backfat thickness (BF), loin muscle depth (LMD), estimated lean meat percentage (ELMP), left teat number (LTN), right teat number (RTN), total teat number (TTN), and time spent eating per day (TPD). The accuracy of genomic prediction was assessed by the correlation between estimated genetic values and phenotypic values using 20 repetitions. In all box plots, the center line represents the median, the bounds of the box represent the 25th and 75th percentiles, and the whiskers represent 1.5× the interquartile range, with outliers plotted as individual points. See the Supplementary Table 9 for more details.

We first evaluated the performance of GBLUP, Adaptive MultiBLUP, BayesR, and IFAM across seven production traits using a public dataset of Duroc pigs (Fig. 4). BayesRC was excluded due to its prohibitive computational time. For the IFAM, we utilized 11 annotation types from the IFmut database combined with trait-specific significant SNPs generated from GWAS, among which the narrow peaks of enhancer annotation showed the highest genomic coverage (Supplementary Fig. 8). Compared to GBLUP, IFAM improved prediction accuracy for all traits, with the largest gain observed for BF (7.90%). Adaptive MultiBLUP, executed with the default chunk size, exhibited considerable variability across replicates, and IFAM improved the accuracy by 52.57% on average compared to it. BayesR, except for TPD, generally underperformed expectations. The non-auto-merged multiple random effect model using the same annotations achieved a prediction accuracy of 0.3416 and a prediction bias of 0.4854 on average (Supplementary Tables 9 and 10), which was significantly worse than IFAM. When pseudo annotations were used, the mean accuracy of IFAM decreased to 0.3700, which was slightly lower than the GBLUP model, highlighting its sensitivity to biologically relevant information. In summary, IFAM demonstrated superior predictive accuracy, robustness, and stability in the pig population, establishing it as an effective method for incorporating functional annotations in livestock breeding.

### Yorkshire pig dataset

**Fig. 5:**
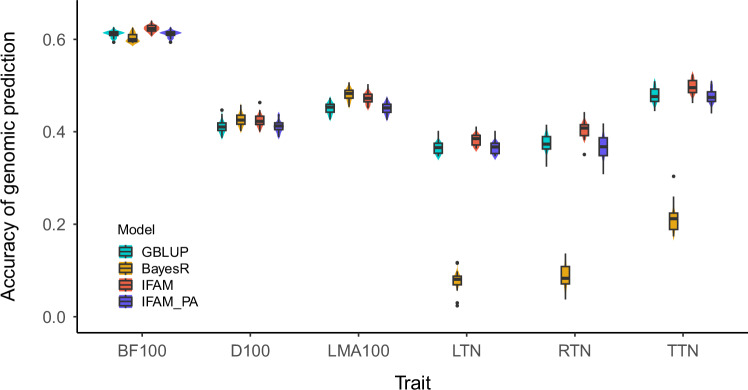
Prediction performance of four models in the Yorkshire pig dataset. The IFAM represents running the IFAM model using trait-specific significant SNPs generated from GWAS and functional annotations derived from the IFmut database. The IFAM_PA represents running the IFAM model using pseudo annotation information, which is selected randomly based on the scale of true annotations from the IFmut database. The data includes the six traits, namely age adjusted to 100 kg (D100), backfat thickness adjusted to 100 kg (BF100), eye muscle area adjusted to 100 kg (LMA100), left teat number (LTN), right teat number (RTN), and total teat number (TTN). The accuracy of genomic prediction was assessed by the correlation between estimated genetic values and phenotypic values using 20 repetitions. In all box plots, the center line represents the median, the bounds of the box represent the 25th and 75th percentiles, and the whiskers represent 1.5× the interquartile range, with outliers plotted as individual points. See the Supplementary Table 11 for more details.

We further evaluated the difference between IFAM, GBLUP, Adaptive MultiBLUP, and BayesR in a larger Yorkshire pig population. For IFAM, the functional annotations were from the IFmut database along with trait-specific significant SNPs generated from GWAS. IFAM improved prediction accuracy by an average of 4.09% compared to GBLUP (Fig. 5). Adaptive MultiBLUP failed to yield meaningful results, as it generated estimated genetic values with zero variance; this underscores its limited applicability to complex livestock traits. Meanwhile, BayesR underperformed substantially on teat number traits, which diminished its overall accuracy (Fig. 5). It is also not feasible to run BayesRC since computational time is pretty long due to the large number of SNP markers. Compared to the non-auto-merged multiple random effect model using the same annotations (Supplementary Tables 11 and 12), IFAM increased the average accuracy by 5.58%, with the most notable improvement for teat number traits. When the pseudo annotations were applied, the accuracy of IFAM decreased substantially, consistent with findings in the Duroc population.

Furthermore, we evaluated the computational efficiency of four models under identical resource allocations. GBLUP was the most efficient (0.30 h), followed by IFAM (0.74 h) and Adaptive MultiBLUP (1.75 h); in contrast, BayesR required several days to complete (Supplementary Table 13). The results highlighted the high computational efficiency of IFAM. Collectively, IFAM achieved the superior overall performance in this large-scale and complex animal population, characterized by high prediction accuracy, competitive efficiency, and exceptional robustness.

### Rice dataset

**Fig. 6:**
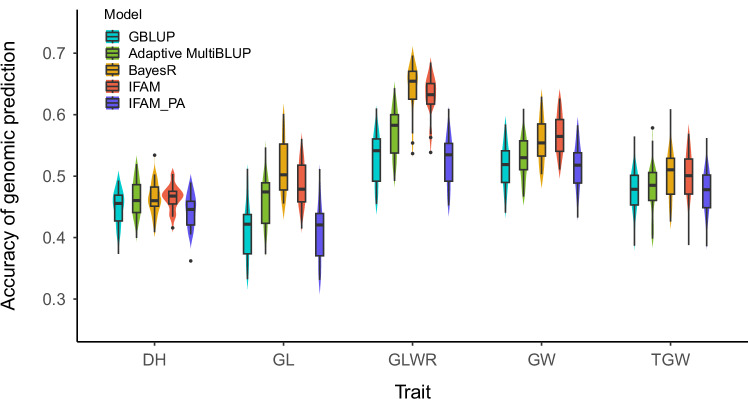
Prediction performance of different models in the rice dataset. The IFAM represents running the IFAM model using trait-specific significant SNPs generated from GWAS and functional annotations derived from the RGAP database. The IFAM_PA represents running the IFAM model using pseudo annotation information, which is selected randomly based on the scale of true annotations from the RGAP database. The data comprises the five traits, namely grain length (GL), grain width (GW), grain length-to-width ratio (GLWR), days to heading (DH), and thousand grain weight (TGW). The accuracy of genomic prediction was assessed by the correlation between estimated genetic values and phenotypic values using 20 repetitions. In all box plots, the center line represents the median, the bounds of the box represent the 25th and 75th percentiles, and the whiskers represent 1.5× the interquartile range, with outliers plotted as individual points. See the Supplementary Table 14 for more details.

We benchmarked GBLUP, Adaptive MultiBLUP, BayesR, BayesRC, and IFAM using a rice dataset (Fig. 6 and Supplementary Tables 14 and 15). IFAM was implemented using 9 functional annotations from the RGAP database, combined with trait-specific significant SNPs. The genomic coverage of functional annotations was detailed in Supplementary Fig. 9. Compared to GBLUP, IFAM improved prediction accuracy by an average of 10.83%, with the most substantial gain observed for GLWR (18.15%). IFAM also consistently outperformed adaptive MultiBLUP, yielding a mean accuracy increase of 5.13%. While IFAM showed a marginal accuracy reduction (1.54%) relative to BayesR, it demonstrated superior prediction bias (Supplementary Table 15). Notably, IFAM outperformed BayesR on GW trait and maintained competitive results for DH and TGW, while reducing runtime from several hours to just a few minutes. Similar to previous experiments, BayesRC yielded anomalous results. Furthermore, IFAM surpassed the non-auto-merged multiple random effect model by 4.88% in accuracy with a more favorable bias (Supplementary Tables 14 and 15). Substituting true annotations with pseudo annotations resulted in a 10.33% accuracy drop, falling slightly below the GBLUP. Overall, these results confirm IFAM’s robustness and efficiency for complex yield-related traits in rice.

## Discussion

We propose IFAM, a precise, efficient, and robust genomic prediction model for complex traits. Traditional genomic prediction models often construct a single relationship matrix from all genetic markers to fit a genetic random effect. These approaches implicitly assume that all marker effects come from the same distribution, which compresses the effect size for some major markers within a limited statistical region, underestimates their true contribution to phenotypic variance, and diminishes prediction accuracy^25^. IFAM addresses this by extending traditional genomic prediction with multiple random effects to incorporate diverse omics data via functional annotation information using individual-level data. The annotations utilized within IFAM are highly flexible, sourced from multiple types and origins (e.g., enhancers, CTCF sites, QTLs, and trait-relevant genes).

Previous attempts to integrate annotation information have primarily focused on summary-level models based on the LD matrices, largely within human genetics^18^, or Bayesian models that impose various assumptions on SNP effects^19^. However, the application of these models is often constrained because the summary statistics remain scarce in non-human research and the computational burden of Bayesian models is substantial. It is not practical to compare IFAM with all rival models, and we focus on individual-level models. Specifically, we compare IFAM with GBLUP and BayesR, the most widely utilized and robust BLUP and Bayesian methods, alongside Adaptive MultiBLUP and BayesRC.

The Adaptive MultiBLUP is an optimized version of the multiple random effect model, which can automatically identify genomic regions with different effect-size variances^6^. Duarte et al.^26^ found that the prediction accuracy of Adaptive MultiBLUP was less accurate than Bayesian methods, but its computational efficiency was valuable at higher marker density. Although Adaptive MultiBLUP outperformed GBLUP on the WTCCC1 and rice datasets, it remained inferior to both BayesR and IFAM. Furthermore, in contrast to its stable performance on the WTCCC1 dataset, Adaptive MultiBLUP exhibited poor and highly variable accuracy across replicates in the pig datasets, even after exhaustive tuning of chunk sizes and significance thresholds (Supplementary Table 16). In the Yorkshire pig population, this model frequently failed, either halting during variance component estimation or by yielding zero-variance predictions within the validation set. We attribute these failures to instabilities in variance component estimation. Moreover, as noted by the developers, high levels of relatedness present in the data will challenge the identification of individual causal loci with relatively strong influence on the phenotype, and the runtime of Adaptive MultiBLUP will be significantly prolonged.

BayesRC, an extension of BayesR, was the first model to integrate functional annotations into livestock genomic prediction^16^. BayesRC is a key comparator for this study. Given the substantial overlap among functional annotations, we utilized an optimized version of BayesRC designed for non-disjoint annotations (See the Supplementary Note for more details)^27^. Contrary to expectations, its performance was suboptimal. Based on the WTCCC1 dataset and functional annotations from the RegulomeDB database, the prediction accuracy of BayesRC remained significantly below both GBLUP and BayesR, regardless of parameter tuning (Supplementary Table 5). We attribute this primarily to annotation complexity and potential non-convergence. Compared to original and subsequent applications of BayesRC typically used only 2–3 pre-processed annotations^16,27^, our study employed raw, overlapping annotations. When we restricted the BayesRC to two annotations (eQTL and matched TF motif), its accuracy improved markedly, rivaling GBLUP, particularly for T1D (Supplementary Table 5). Furthermore, although increasing MCMC iterations to 100,000 yielded minor improvements, the gains were insignificant. Given its computational intensity and lack of parallelization, further increasing iteration is impractical. These results suggested that BayesRC requires high-quality, trait-relevant annotations to perform optimally. Since previous studies indicated that BayesRC generally offers marginal gains over BayesR^27–29^, a baseline method included in our comparisons, this limitation does not undermine the comparative assessment of IFAM against Bayesian approaches.

A core innovation of IFAM is its data-adaptive strategy for merging annotations with similar variance component estimates (10X). This is motivated by the challenge that when numerous annotations are treated as independent random effects, their variance estimates often converge to local optimal, thereby compromising prediction accuracy. Our results (Supplementary Table 8, Supplementary Tables 17–20, and Supplementary Data 2) confirmed that the total genetic variance estimates become either inflated or attenuated if the random effects are not merged, particularly in the UKB and Duroc pig populations, leading to reduced predictive performance, and this instability was further exacerbated by the number of random effects increasing. The choice of 10X difference as the merging criterion was based on two considerations. First, it effectively groups annotations with a similar order-of-magnitude contribution to total genetic variance, mitigating the impact of overlapping SNPs among annotations, simplifying the model, and enhancing the stability of variance component estimation. Second, this threshold aligns with the BayesR model^8^, which categorizes effect sizes (e.g., explaining 0.01%, 0.1%, and 1% of the total genetic variance) into distinct, order-of-magnitude classes. We found this rule to be robust and generally applicable for capturing the genetic architecture of diverse complex traits.

In IFAM, it is flexible and straightforward to utilize functional annotations derived from diverse sources. Our results indicated that the performance of IFAM does not necessarily improve with the sheer number of annotations but is more closely tied to their relevance to the target trait (Fig. 2). Furthermore, the quality of annotations, tissue and trait relevance, the number and interpretation of overlapping annotations, and the resolution of existing functional datasets are factors as well. Initially, we opted for easily accessible functional annotations as proxies for multi-omics data to avoid the complexities of omics data integration, given their general applicability across multiple traits within a species. However, preliminary experiments revealed that we had overestimated the capacity of current functional annotations to explain the phenotypic variation of interesting traits, which represent only a fraction of the complex regulatory landscape underlying causal variants. Acknowledging this limitation, referring to other studies^30–32^, such as the adaptive MultiBLUP^6^ and BayesRC^16^, which utilized QTLs or SNPs significantly associated with the target trait to improve the accuracy of genomic prediction. We incorporated trait-specific significant association signals as an annotation category to enhance biological relevance. Notably, excluding trait-specific GWAS-derived annotations, functional annotations alone yielded a 1.40% predictive gain for UKB dataset but only marginal improvements in livestock and crop datasets. Given that the performance of IFAM improves alongside increases in population size and annotation quality, this gain is poised to increase as multi-omics resources become more comprehensive.

There is no limitation for genomic data in IFAM, which applies to studies using sequencing or custom genotyping arrays. Our method currently focuses on additive genetic effects, but it could be readily extended to handle non-additive genetic effects. Although our proposed model has enabled powerful analysis of omics data, it has some constraints. For example, it is assumed that all SNPs within each functional annotation region contributed equally to the trait, which may not fully align with biological reality, as certain causal variants likely exert larger effects. Non-linear models, such as deep learning, could help assess the reliability of functional annotations and perform genomic prediction in the future^33^.

In summary, the proposed IFAM model overcomes the barriers between multi-omics data and classical genomic breeding data, which facilitate automatic adjustment of the model to accommodate diverse multi-omics annotations for different species, thereby achieving accurate prediction of complex traits. It is anticipated that this advancement will enhance the accuracy of predicting human diseases and agricultural economic traits beyond the current reliance on “genomic breeding value” to a more comprehensive “omics breeding value” paradigm. We believe that IFAM would be increasingly beneficial as the volume of functional annotation data expands and the quality of these data improves.

## Methods

### Ethics

All research was conducted in accordance with the relevant guidelines and the criteria set by the Declaration of Helsinki. This study involved the secondary analysis of de-identified, publicly available data from the UK Biobank and the WTCCC. The UK Biobank received ethical approval from the North West Haydock Research Ethics Committee (reference no. 21/NW/0157), and all participants provided informed consent. The WTCCC project was approved by the South East Multicenter Research Ethics Committee (reference no. 05/Q0106/74), and all participants provided written informed consent. The Animal Ethics Committee of Huazhong Agricultural University granted approval for the experimental designs and procedures (approval no: [HZAUSW20260016]). Rigorous measures were undertaken to assure the well-being and humane handling of the Yorkshire pigs engaged in this study. The gathering of specimens and data was executed in conformity with the pertinent regulations and directives on animal welfare and protection.

### Data

*WTCCC1 dataset*: The Welcome Trust Case-Control Consortium (WTCCC1) dataset, which was designed to facilitate advancements in comprehending the genetic mechanisms that underlie common diseases, is utilized extensively for evaluating the performance of various genomic prediction models^34^. Therefore, we chose this dataset to comprehensively test the performance of IFAM model. The WTCCC1 dataset consists of seven binary disease traits, namely bipolar disorder (BD, *N* = 4806), coronary artery disease (CAD, *N* = 4864), Crohn’s disease (CD, *N* = 4686), hypertension (HT, *N* = 4890), rheumatoid arthritis (RA, *N* = 4798), type 1 diabetes (T1D, *N* = 4901), and type 2 diabetes (T2D, *N* = 4862). The genotypic data from the WTCCC1 dataset were acquired through high-throughput genotyping techniques, which encompassed approximately 450,000 SNPs per individual. Genotypic data quality control was conducted as described previously^6,8^. Briefly, SNPs were excluded using PLINK software (v1.90) based on the following criteria: minor allele frequency (MAF) < 0.01, genotype call rate < 0.95, and *P*-value < 0.05 from the Hardy-Weinberg equilibrium test^35^. The detailed information on the remaining data was shown in the Supplementary Table 21.

*UK Biobank dataset*: The UK Biobank is a large-scale biomedical database, which contains in-depth genetic and health information from approximately half a million UK participants^36^. The UKB dataset is utilized to assess the feasibility and effectiveness of IFAM model in big data analysis. The data processing followed the procedure described in the study by Lloyd-Jones et al.^37^. Briefly, the HapMap3 SNPs, genotyped in 2504 participants of the 1000 Genomes Project, were extracted from the UKB dataset, the SNPs with MAF > 0.01, probability of departure from Hardy-Weinberg equilibrium <${10}^{-6}$, and missingness < 0.05 were retained, which resulted in 1,094,840 SNPs. Genotypic principal components were used to infer ancestry and subpopulation structure, and a subset of 348,501 unrelated (absolute genomic relationship matrix off-diagonal < 0.05) individuals of Europe was selected for analysis^37^. The six representative traits with diverse genetic architectures (from less to highly polygenic) were selected for subsequent analysis, namely height (HT, *N* = 318,324), basal metabolic rate (BMR, *N* = 313,495), heel bone mineral density T-score (hBMD, *N* = 181,028), forced vital capacity (FVC, *N* = 291,054), body mass index (BMI, *N* = 317,989), and forced expiratory volume in 1 s (FEV, *N* = 291,054). All phenotypes were pre-adjusted for age, sex, and the first 10 PCs. The residuals were standardized to have mean zero and unit variance and finally rank-based inverse-normal transformed.

*Duroc pig dataset:* A published Duroc pig dataset^38^ was downloaded to assess the compatibility and power of IFAM model for economic traits in agricultural animals. This dataset comprised genotypic and phenotypic information from 2797 boars with 11,348,460 SNPs, and the genotypes were obtained using a low-coverage, whole-genome sequencing strategy^38^. Quality control was applied to the imputed genotypic data by filtering out SNPs with MAF < 0.01, genotype call rate < 0.90, and individual call rate < 0.90 using Plink software (v1.90)^35^. A total of 11,348,241 SNPs were retained for all 2796 pigs. The phenotypic data comprised the seven available traits, namely backfat thickness (BF, *N* = 2771), loin muscle depth (LMD, *N* = 2789), estimated lean meat percentage (ELMP, *N* = 2782), left teat number (LTN, *N* = 2796), right teat number (RTN, *N* = 2796), total teat number (TTN, *N* = 2796), and time spent eating per day (TPD, *N* = 2600).

*Yorkshire pig datase: *A total of 16,783 Yorkshire pigs were recruited from a commercial breeding farm and all pigs were raised under standardized husbandry conditions. Tail tissue samples were collected from each pig during the routine tail-docking procedure for piglets. This non-invasive integration into standard farm management practices minimized additional stress to the animals. Immediately after collection, the tail tissues were preserved in 75% ethanol and stored at −20 °C to maintain DNA integrity. All preserved samples were transported to Yingzi Gene Technology Co., Ltd (Wuhan, China) for genomic analysis. Genomic DNA was extracted from the tail tissues using standard protocols. High-throughput genotyping was subsequently performed using the pig 80 K functional variants genotyping array. The genotype data with 187,000 SNPs were imputed using the reference panel and the imputation tool from the AGIDB website (http://animalbreedinglab.hzau.edu.cn/AGIDB/home/home.php), and variants with imputation accuracy *R*^2^ > 0.7 were retained^39^. Following the same quality control protocols applied to the Duroc pig dataset, a total of 15,457,739 SNPs were reserved for analysis. We collected five key production traits at the end of the finishing period (approximately 120 kg body weight), including the age adjusted to 100 kg (D100, *N* = 16,732), backfat thickness adjusted to 100 kg (BF100, *N* = 16,673), eye muscle area adjusted to 100 kg (LMA100, *N* = 15,852), left teat number (LTN, *N* = 13,726), right teat number (RTN, *N* = 13,726), and total teat number (TTN, *N* = 13,726). All phenotypes were pre-adjusted for sex, line, and year-season.

*Rice dataset*: A rice dataset from the 3000 Rice Genome Project was downloaded to evaluate the broad applicability of IFAM model for yield-related traits in agricultural plant^40^. The genotype was derived from 3024 rice accessions, providing a comprehensive representation of genetic and functional diversity. Quality control was applied to the genotype data by filtering out SNPs with MAF < 0.01, genotype call rate < 0.90, and individual call rate < 0.90 using Plink software (v1.90), and a total of 333,454 SNPs were retained^35^. Five representative yield-related traits were used for genetic evaluation, namely grain length (GL, *N* = 2011), grain width (GW, *N* = 2011), grain length-to-width ratio (GLWR, *N* = 2011), days to heading (DH, *N* = 2718), and thousand grain weight (TGW, *N* = 1787). All phenotypes were pre-adjusted for the original country and rice variety.

*Genomic functional annotation information:* The functional genomic annotation information was obtained from the RegulomeDB database and the LD Scores Regression (LDSC) model for human, the IFmut database for pig, and the Rice Genome Annotation Project (RGAP) database for rice^24,41–43^. To gather comprehensive functional annotation information on common variants (MAF > 0.01) in humans, the annotations from the RegulomeDB database were downloaded^41^, which offered functional context and ranking scores. The functional context included expression quantitative trait loci, transcription factor binding sites, deoxyribonuclease (DNase) footprints, and others (Supplementary Table 22). The ranking scores of SNP had seven levels; in general, if more supporting data were available, the higher its likelihood of being functional, hence, it received a higher score (i.e., with 1 being the highest score and 7 being the lowest score) (Supplementary Table 23). Another set of annotations from the LDSC model was also used^24^, which provided up to 74 binary annotations, including coding region, conserved sites, CCCTC-binding factor (CTCF) sites, and others (Supplementary Table 24). The IFmut database provided various epigenomic regulatory elements of the pig genome, such as open chromatin region and nucleosome free region (Supplementary Table 25)^43^. The RGAP database provided annotation data for the rice genome to understand gene function in a genomic context, including coding sequence, exon, and others (Supplementary Table 26)^42^. The same version of reference genome was used for genomic data and annotation data.

In addition to species-specific functional annotation information, the SNPs associated with target traits were viewed as a type of trait-specific annotation. We employed the general linear model (GLM) for disease traits from the WTCCC1 dataset and mixed linear model (MLM) for agricultural traits from the Duroc pig, Yorkshire pig, and rice datasets using the rMVP software (v1.0.8) to perform the GWAS analysis in the training set^44^. We used the Bonferroni threshold to detect significant hits^45^, then extracted all markers as trait-specific significant SNPs that are located within a 100 kb interval upstream and downstream of the significant hits. Due to the scale of the UKB dataset, the linear regression model was used to conduct the GWAS analysis in the training set by Plink software (v1.90)^35^.

### Model

#### IFAM

Aimed at enhancing the accuracy of genomic predictions of complex traits, we proposed a powerful model we call IFAM, which can effectively integrate functional annotation information via a linear mixed model with multiple random effects. First, the SNPs across the entire genome were partitioned into multiple sets based on the coordinates of different types of functional annotations, supposing $C$ annotation types were used here. Second, it is recommended to construct genomic relationship matrices (GRMs) in advance using the SNPs within each annotation type, which will save computational time if performing cross-validation. Then, fitting a GBLUP model with multiple random effects, where each annotation type was treated as a random effect, to predict the genetic value of individuals as follows:

1 $${\bf{y}}={\bf{Xb}}+\sum _{k}^{C}{{\bf{Z}}}_{k}{{\bf{g}}}_{k}+{\bf{e}}$$

where ${\bf{y}}$ is a vector of phenotypic values, which are binary values (case 1, control 0) for disease traits or continuous values for quantitative traits; ${\bf{b}}$ is a vector of fixed effects, ${\bf{X}}$ is the corresponding incidence matrix; ${{\bf{g}}}_{k}$ is a vector of genetic effects of the *k*th annotation type, following a multivariate normal distribution ${{\bf{g}}}_{k} \sim {\rm{N}}\left(0,\,{{\bf{G}}}_{k}{\sigma }_{k}^{2}\right)$, where ${{\bf{G}}}_{k}$ is the GRM that was constructed using SNPs within the *k*th annotation type by the VanRaden method as follows^4^:

2 $${{\bf{G}}}_{k}=\frac{{{\bf{M}}}_{k}{{\bf{M}}}_{k}^{{\prime} }}{2\sum {p}_{i}\left(1-{p}_{i}\right)}$$

where ${{\bf{M}}}_{k}$ is the marker covariates matrix, in which values are coded as 0, 1, and 2 for homozygote, heterozygote, and another homozygote, respectively; ${p}_{i}$ is the frequency of the minor allele at locus $i$; ${\sigma }_{k}^{2}$ is the genetic variance explained by SNPs in the *k*th annotation type; ${{\bf{Z}}}_{k}$ is the incidence matrix for random genetic effects; ${\bf{e}}$ is a vector of residual effects, following the independent and identically normal distribution ${\bf{e}} \sim {\rm{N}}\left(0,\,{\bf{I}}{\sigma }_{e}^{2}\right)$ with ${\bf{I}}$ being an identity matrix, and ${\sigma }_{e}^{2}$ is the residual variance.

In theory, the IFAM model has the capability of incorporating various types of functional annotations as multiple random genetic effects in a linear mixed model. However, there is an obvious overlap of SNPs between different annotation categories (Supplementary Figs. 4, 10, and 11), which may affect variance component estimation and model robustness. The estimated variance components of REML algorithm have the possibility of falling into a local optimal solution if the model has many random effects, resulting in poor prediction performance (Supplementary Table 8, Supplementary Tables 17–20, and Supplementary Data 2). To solve it, the random effects generated by annotation categories with approximate variance components (within 10X) were merged, referring to the study of BayesR^8^, since they have similar contributions to the phenotypic variance. Specifically, there were $C$ variance estimates for $C$ annotations obtained through formula 1, these estimates will be merged according to the following variance component range (${R}_{{vc}}$):

3 $${R}_{{vc}}={\sigma }_{\max }^{2}/{10}^{{C}_{o}} < {\sigma }_{k}^{2}\le {\sigma }_{\max }^{2}/{10}^{{C}_{o}-1}$$

where ${C}_{o}$ (${C}_{o}\le C$) is the assumed number of random effects after merging annotations, ${\sigma }_{\max }^{2}$ is the maximum value among variances. Lastly, the ${C}_{o}$ sets of annotations were used to calculate the final genetic values according to formula 1.

This processing not only mitigated the impact of overlapping SNPs among annotations but also reduced the number of random effects in the IFAM model, potentially addressing the non-convergence of iteration and abnormal variance estimates. Besides, this processing aligned with the premise of the multi-random effect model (i.e., the ignorance of covariance between random terms), which facilitated the utilization of massive types of functional annotations in the IFAM model.

#### Evaluating functional annotations

For functional annotations, assessing their significance to complex traits is also essential for the design of Chip array and the dissection of genetic architectures. A straightforward and efficient strategy is to calculate the average phenotype variance explained (PVE) by per-SNP within functional annotation regions, which served as indicators of the genetic contribution of functional annotations. However, there are different levels of LD in these regions, and the estimates will be underestimated if not properly accounted for^46^. Therefore, we used the variance components ${\sigma }_{k}^{2}$ and narrow heritability ${h}_{k}^{2}$ of functional annotation $k$ divided by the number of SNPs after pruning (${m}_{k}$), which yielded ${\sigma }_{k}^{2}/{m}_{k}$ and ${h}_{k}^{2}/{m}_{k}$, and called them per-SNP explained variance and heritability. The average information restricted maximum likelihood algorithm (AI-REML) algorithm was used to estimate variance components and heritability^47^. When dealing with a larger population (e.g., over 100,000), we chose the Haseman–Elston (HE) regression method to estimate genetic parameters^48^. SNP pruning was performed by PLINK software (v1.90) (*--indep-pairwise*): the window size and step size were set to 1000 and 100, and the pairs of variants in the current window with squared correlation greater than 0.2 were pruned^35^. We validated this strategy by comparing our estimates against the established ranking scores of SNP from the RegulomeDB database, where high concordance would confirm the efficacy of our approach.

#### Cross-validation and prediction accuracy

We evaluated the predictive performance of IFAM model across the five datasets. For all datasets except the UKB, individuals were randomly split into a training set that contained 80% of the individuals and a validation set that contained the remaining 20%, and this process was repeated 20 times^49^. Given the scale of UKB dataset, we performed 10 times 80%/20% random splits for each trait to save computational time.

The prediction accuracy of different models was measured using Pearson’s correlation for quantitative traits and the area under the curve (AUC) statistic for disease traits in the validation set^50^. Statistical significance between IFAM and competing models was assessed via the corrected resampled two-sided t-test using correctR R package (v0.3.1)^51^. Additionally, the regression coefficient when regressing phenotypic values on estimated genetic values in the validation set was calculated as the bias of prediction, which is expected to be 1 if there is no over-dispersion or under-dispersion of estimated genetic values.

### Reporting summary

Further information on research design is available in the Nature Portfolio Reporting Summary linked to this article.

## Supplementary information

Supplementary information for manuscript

Description of Additional Supplementary Files

Supplementary Data 1

Supplementary Data 2

Supplementary Data 3

Reporting Summary

Transparent Peer Review file

## Source data

Source data file

## Data Availability

Individual-level genotype and phenotype data for height, basal metabolic rate, heel bone mineral density T-score, forced vital capacity, body mass index, and forced expiratory volume in 1 s from the UK Biobank (2017 release) were obtained under Application Number 97563. Researchers can request access to UK Biobank data on https://www.ukbiobank.ac.uk/enable-your-research/apply-for-access. The human case-control data were obtained from the WTCCC1 study, specifically covering seven diseases: bipolar disorder, coronary artery disease, Crohn’s disease, hypertension, rheumatoid arthritis, type 1 diabetes, and type 2 diabetes. Access to these data is managed by the Wellcome Trust Case Control Consortium [http://www.wtccc.org.uk/]. The Duroc pig dataset (low coverage whole-genome sequencing and agricultural economic traits) is available via GigaDB [10.5524/100894]. The Yorkshire pig genotype and six production traits^52^ generated in this study have been deposited on Figshare [10.6084/m9.figshare.30985417], and the source genotyping data will be available on the EVA under accession number PRJEB111159 on April 1 ^st^ 2028. The 3000 Rice Genomes Project SNP arrays and phenotype records are accessible at https://iric.irri.org/projects/3000-rice-genomes-project. Humans functional annotations were obtained from the RegulomeDB database [https://www.regulomedb.org/regulome-search] and the LD Scores Regression model [https://alkesgroup.broadinstitute.org/LDSCORE/baselineLD_v2.2_bedfiles.tgz/]. Pig functional annotations are available from the IFmut database [http://www.ifmutants.com:8212/#/download]. Rice genomic annotations were obtained from the Rice Genome Annotation Project Database [https://rice.uga.edu/pub/data/Eukaryotic_Projects/o_sativa/annotation_dbs/pseudomolecules/version_7.0/all.dir/]. Source data are provided with this paper.

## Code availability

The IFAM software code is available at: https://github.com/xiaolei-lab/IFAM, under the MIT license. Codes for the version of IFAM used in this paper are also deposited at Zenodo^53^ [10.5281/zenodo.18802709].
