# Supplementary material for: Improving genomic prediction accuracy of complex traits by integrating massive types of functional annotation information
Source: Nat Commun. 2026 Apr 24;17:5686. doi: 10.1038/s41467-026-72470-0 (PMC13319817; doi:10.1038/s41467-026-72470-0)
Supplement: Supplementary file 1 — Supplementary information for manuscript [file 41467_2026_72470_MOESM1_ESM.pdf]

Supplementary information for

Improving Genomic Prediction Accuracy of Complex Traits by Integrating Massive Types of Functional Annotation Information

Zhenshuang Tang[1,2], Xiong Xiong[2,3], Haohao Zhang[4], Dong Yin[2], Yuhua Fu[2,3,5,6], Yunxia Zhao[1,2], Jingjin Li[2], Yuan Quan[7,8], Xiang Zhou[9,10], Xinyun Li[2,3,5,6,11], Lilin Yin[2,3,5,6*], Shuhong Zhao[1,2,3,5,6,11*], and Xiaolei Liu[2,3,5,6*]

[1] *Yazhouwan National Laboratory, Sanya 572024, China;*
[2] *College of Animal Science and Technology, Huazhong Agricultural University, Wuhan 430070, China;*
[3] *Hubei Hongshan Laboratory, Wuhan 430070, China;*
[4] *School of Computer Science and Technology, Wuhan University of Technology, Wuhan 430070, China;*
[5] *Agricultural Animal Genetics, Breeding and Reproduction, Ministry of Education, Wuhan 430070, China;*
[6] *Frontiers Science Center for Animal Breeding and Sustainable Production, Ministry of Education, Wuhan 430070, China;*
[7] *College of Informatics, Huazhong Agricultural University, Wuhan 430070, China;*
[8] *Hubei Key Laboratory of Agricultural Bioinformatics, Huazhong Agricultural University, Wuhan 430070, China;*
[9] *Department of Biostatistics, University of Michigan, Ann Arbor, MI, USA;*
[10] *Center for Statistical Genetics, University of Michigan, Ann Arbor, MI, USA;*
[11] *Key Laboratory of Swine Genetics and Breeding, Ministry of Agriculture and Rural Affairs, Wuhan 430070, China;*
[*] *Correspondence: Lilin Yin (ylilin@mail.hzau.edu.cn), Shuhong Zhao (zhaoshuhong@yzwlab.cn), Xiaolei Liu (xiaoleiliu@mail.hzau.edu.cn)*

Supplementary Note

Genomic best linear unbiased prediction (GBLUP)

The GBLUP model is a statistical approach utilized for estimating the genetic value of individuals in livestock and plant breeding and for predicting polygenic risk scores (PRS) for diseases in humans[1, 2]. GBLUP assumes that all genetic markers contribute equally to genetic variation, which implies the absence of major genes across the genome. There are two equivalent models for GBLUP prediction. The first model

estimates individual marker effects, summing them to obtain the genomic estimated breeding values (GEBV), which is commonly known as ridge regression BLUP[3]. The second model, which we employed in this study, replaces the traditional genetic relationship matrix that is derived from pedigree data with the genomic relationship matrix[4]. The latter, which is referred to as GBLUP[5], is represented by the equation:

$$\mathbf{y} = \mathbf{Xb} + \mathbf{Zg} + \mathbf{e}$$

where $\mathbf{y}$ is a vector of phenotypic values, which are binary values (case 1, control 0) for disease traits or continuous values for quantitative traits; $\mathbf{b}$ is a vector of fixed effects, $\mathbf{X}$ is the corresponding incidence matrix; there is only one random genetic effect ($\mathbf{g}$) in this model, which is called the GEBV, $\mathbf{g}$ follows a multivariate normal distribution $\mathbf{g} \sim \mathrm{N}(0, \mathbf{G}\sigma_g^2)$, where $\mathbf{G}$ is the genomic relationship matrix constructed using all SNPs, and σ_g^2 is the genetic variance, $\mathbf{Z}$ is the incidence matrix for random genetic effect; $\boldsymbol{e}$ is a vector of residual effects, following the independent and identically normal distribution $\mathbf{e} \sim \mathrm{N}(0, \mathbf{I}\sigma_e^2)$ with $\mathbf{I}$ being an identity matrix, and σ_e^2 is the residual variance.

BayesR and BayesRC

To evaluate the differences in prediction performance between IFAM and Bayesian method, BayesR[6] and BayesRC[7] were selected to conduct benchmark tests. BayesR (Bayesian multiple regression) belongs to a Bayesian mixture model and is typically employed to calculate risk predictors for diseases in humans or GEBV for economic traits in livestock[6]. Compared to models within the BLUP framework, Bayesian models enhance predictive ability by setting different statistical priors, albeit with relatively

lower computational efficiency. The standard linear regression model that relates phenotype to markers is expressed as follows:

$$\mathbf{y} = \mathbf{1}_n\boldsymbol{\mu} + \mathbf{M}\boldsymbol{\beta} + \mathbf{e}$$

where $\mathbf{y}$ is a vector of phenotypic values as mentioned previously; $\mathbf{1}_n$ is an *n*-dimensional vector of ones, $\boldsymbol{\mu}$ is the general mean; $\mathbf{M}$ is a numeric matrix for all SNP markers with a dimension of *n* by *m* (*n* and *m* is the number of individuals and SNP markers, respectively); $\boldsymbol{\beta}$ is a vector of estimated marker effects; and $\mathbf{e}$ is a vector of residuals, following $\mathbf{e} \sim \mathrm{N}(0, \mathbf{I}\sigma_e^2)$. The prior assumption of the BayesR model is that SNP effects ($\boldsymbol{\beta}$) are derived from a mixture of four zero-mean normal distributions, where the relative variance for each mixture component is fixed:

$$\mathrm{f}(\boldsymbol{\beta}_i) = \sum_{k=1}^{4} \boldsymbol{\pi}_k \mathrm{f}_k\,(\boldsymbol{\theta}_k)$$

$$\mathrm{f}_k = \begin{cases} \delta(0) & \text{if } k = 1 \\ \phi(0, \boldsymbol{\theta}_k) & \text{otherwise} \end{cases}$$

where $\boldsymbol{\theta} = (\boldsymbol{\theta}_2,\ \boldsymbol{\theta}_3,\ \boldsymbol{\theta}_4) = (0.0001\sigma_g^2,\ 0.001\sigma_g^2, 0.01\sigma_g^2)$, $\sum_{k=1}^{4} \boldsymbol{\pi}_k = 1$, $\delta(0)$ represents a point mass at 0, and ϕ is the centered Gaussian probability density function, σ_g^2 is the genetic variance. BayesR (bayesRv2) software was used in this study, the MCMC chain length was set to 50,000, and the burn-in step was set to 20,000 for WTCCC1 and Rice datasets; the MCMC chain length was set to 10,000, and the burn-in step was set to 4,000 for UKB, Duroc pig, and Yorkshire pig datasets (Supplementary Table 27).

The BayesRC model incorporated prior biological information on the basis of BayesR model, for further details about BayesRC, see the study of MacLeod et al.[7]. Due to the BayesRC is not adapted to cases where markers may have multiple annotations, we

chose the BayesRC+ approach, which was an enhanced version of BayesRC through a cumulative way to account for the contribution of multiple annotation categories and was implemented in the BayesRCO software (v0.0.2)[8], and we continuedly used the term BayesRC representing the BayesRC+ for understanding. A cumulative mixture prior distribution for the SNP effects was defined as follows:

$$\mathrm{f}(\boldsymbol{\beta}_i|\mathbf{C}_i) = \sum_{c\in\mathbf{C}_i}\sum_{k=1}^{4}\boldsymbol{\pi}_{k,c}\mathrm{f}_k\,(\boldsymbol{\theta}_k)$$

$$\mathrm{f}_k = \begin{cases} \delta(0) & \text{if } k = 1 \\ \phi(0,\boldsymbol{\theta}_k) & \text{otherwise} \end{cases}$$

where parameters were defined as before and $\sum_{k=1}^{4}\boldsymbol{\pi}_{k,c} = 1$ for all $c \in \{c_1, c_2, \dots, c_m\}$, c_m is mth annotation. The core of BayesRCO (v0.0.2) software is based on the BayesR (v0.75) software, so it cannot perform parallel computing. We ran the BayesRC model with the default parameter set, where the MCMC chain length was set to 50,000, and the burn-in step was set to 20,000.

Adaptive MultiBLUP

The adaptive MultiBLUP model was proposed in Speed and Blading and implemented in the LDAK software[9]. This model used local genomic relationship matrices to automatically identify and weight genomic regions that were significantly associated with the trait of interest. In this study, the adaptive MultiBLUP (v6.0.0) was run to conduct the benchmark test for IFAM. There are six steps to run the basic adaptive MultiBLUP. It starts to divide the genome into chunks and to test the chunks for association, where the chunk size was set to 75,000 base pairs (default). Then, a likelihood ratio test was performed to test whether the variance of region was significantly different from 0, which the significance thresholds was set to 0.05

Bonferroni corrected for the number of chunks, if the threshold was greater than the top 20th smallest *P*-value, the top 20th smallest *P*-value was selected as the new threshold, mainly to avoid the difficulty in estimating the variance components due to an excessive number of regions. The background relationship matrix was calculated and the MultiBLUP model was used to estimate variance components. Finally, the genomic predictions were obtained by using several genomic relationship matrices and the estimated variance components. See the paper of MultiBLUP model for more details

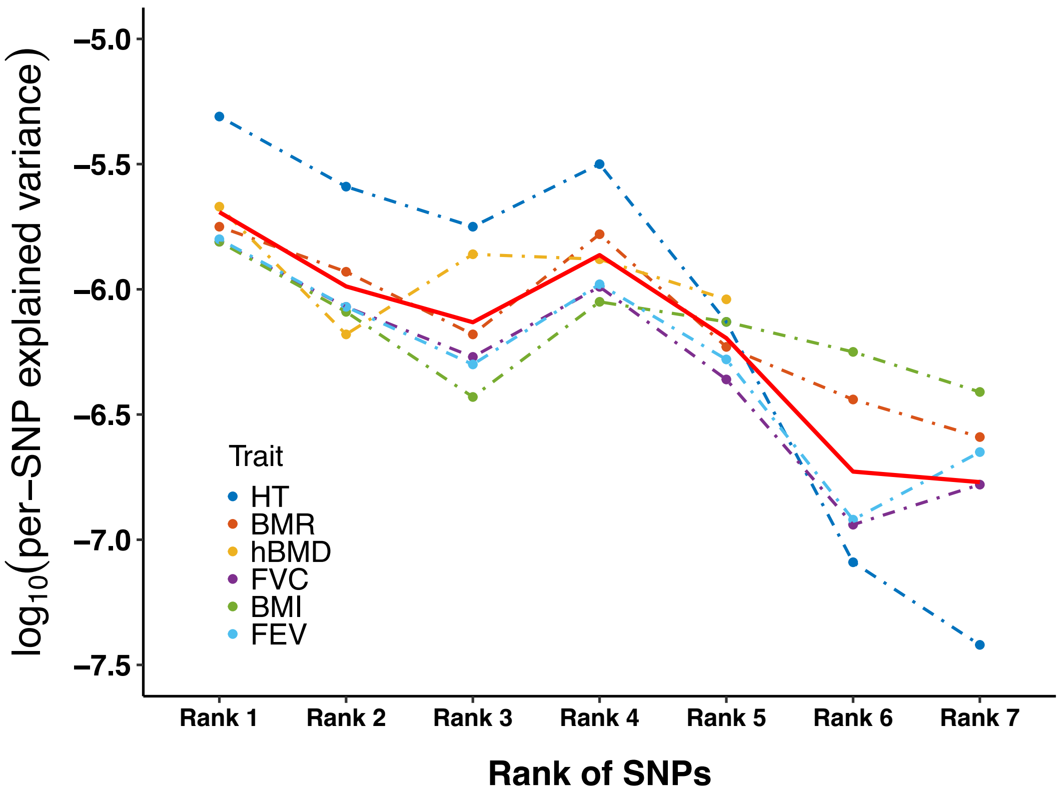

Supplementary Fig. 1. Examples of the importance assessment of functional annotation information using the UK Biobank (UKB) dataset. The log10 scale of the per-SNP explained variance (y-axis) was estimated from the non-auto-merged multiple random effect model for each ranking score (x-axis), which are from the RegulomeDB database. The Haseman–Elston (HE) regression algorithm was chosen to estimate the variance component. It needs to declare that the phenotypic values of the UKB dataset were pre-adjusted, which resulted in a phenotypic variance of 1, and this led to an identical per-SNP variance component and heritability for each category. The UKB dataset includes six traits, namely height (HT), basal metabolic rate (BMR), heel bone mineral density T-score (hBMD), forced vital capacity (FVC), body mass index (BMI), and forced expiratory volume in 1s (FEV). The dotted lines of different colors represent the values of different traits, and the solid red line is the mean value of all traits. See the Supplementary Table 28 for more details.

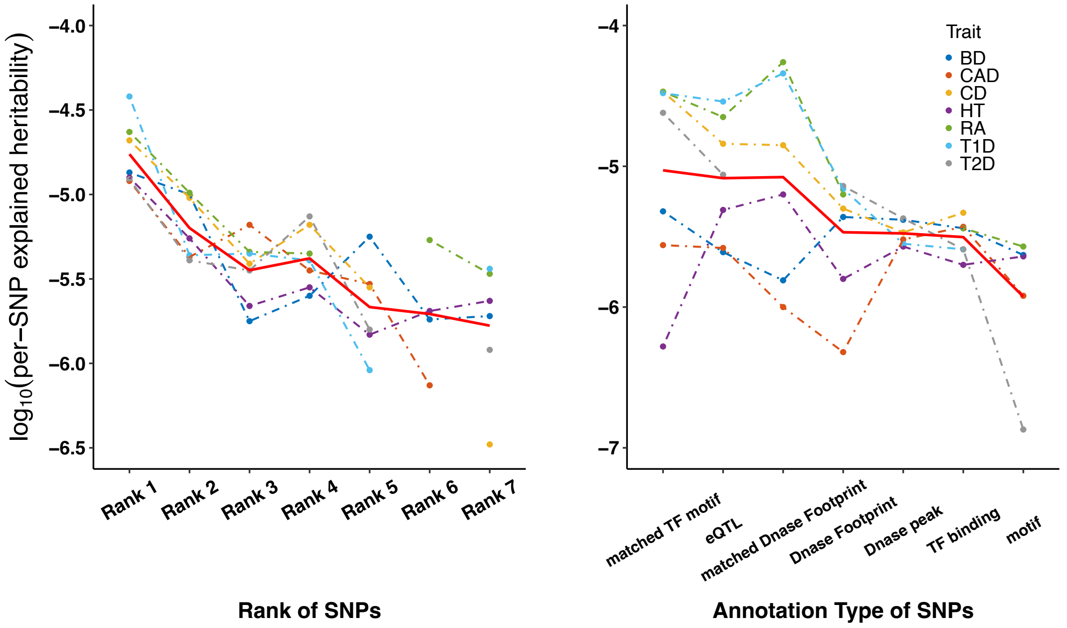

Supplementary Fig. 2. Examples of the importance assessment of functional annotation information and the criterion of assessment is the heritability using the WTCCC1 dataset. The log10 scale of the per-SNP explained heritability (y-axis) was estimated from the non-auto-merged multiple random effect model for ranking scores of SNPs (left) and annotation types of SNPs (right), which are from the RegulomeDB database. The average information restricted maximum likelihood (AI-REML) algorithm was chosen to estimate the heritability. The WTCCC1 dataset consists of seven binary disease traits, namely bipolar disorder (BD), coronary artery disease (CAD), Crohn's disease (CD), hypertension (HT), rheumatoid arthritis (RA), type 1 diabetes (T1D), and type 2 diabetes (T2D). The dotted lines of different colors represent the values of different traits, and the solid red line is the mean value of all traits. Supplementary Tables 29-30 for more details.

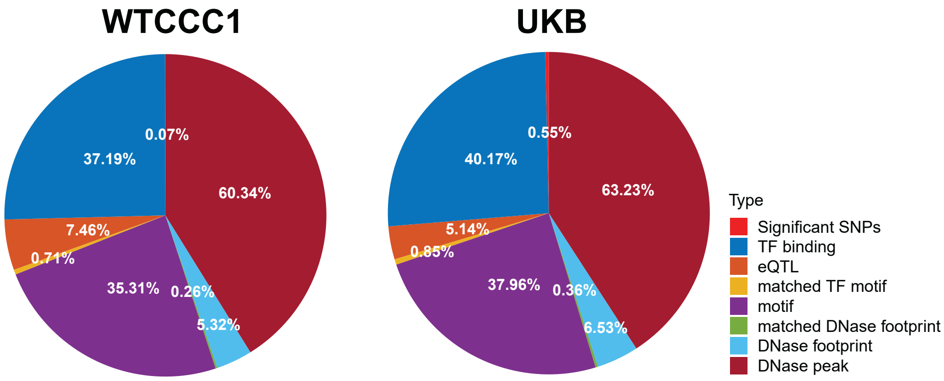

Supplementary Fig. 3. The average proportion of SNPs with distinct functional annotations across 7 traits within the WTCCC1 (left) and UK Biobank (UKB, right) datasets. The annotation process utilized the 7 functional annotations from the RegulomeDB database along with trait-specific significant SNPs generated from GWAS. See the Supplementary Tables 31-32 for more details.

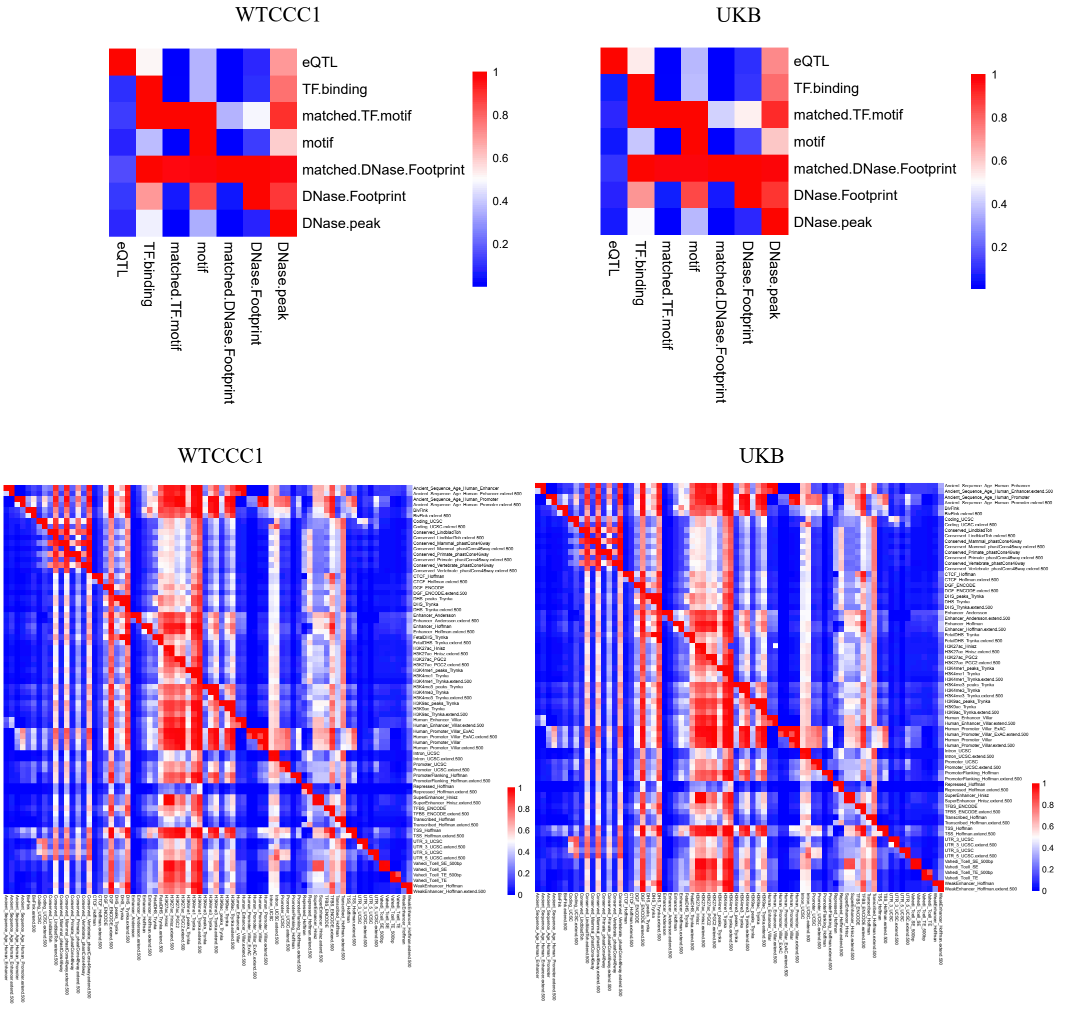

Supplementary Fig. 4. The heatmap of overlap ratio between functional annotations from the RegulomeDB database (top) and the LDSC model (bottom) based on the WTCCC1 dataset and the UK Biobank (UKB) dataset. The upper triangle represented the proportion of the number of SNPs overlapping A1 and A2 to A1, and the lower triangle represented the proportion of the number of SNPs overlapping A1 and A2 to A2.

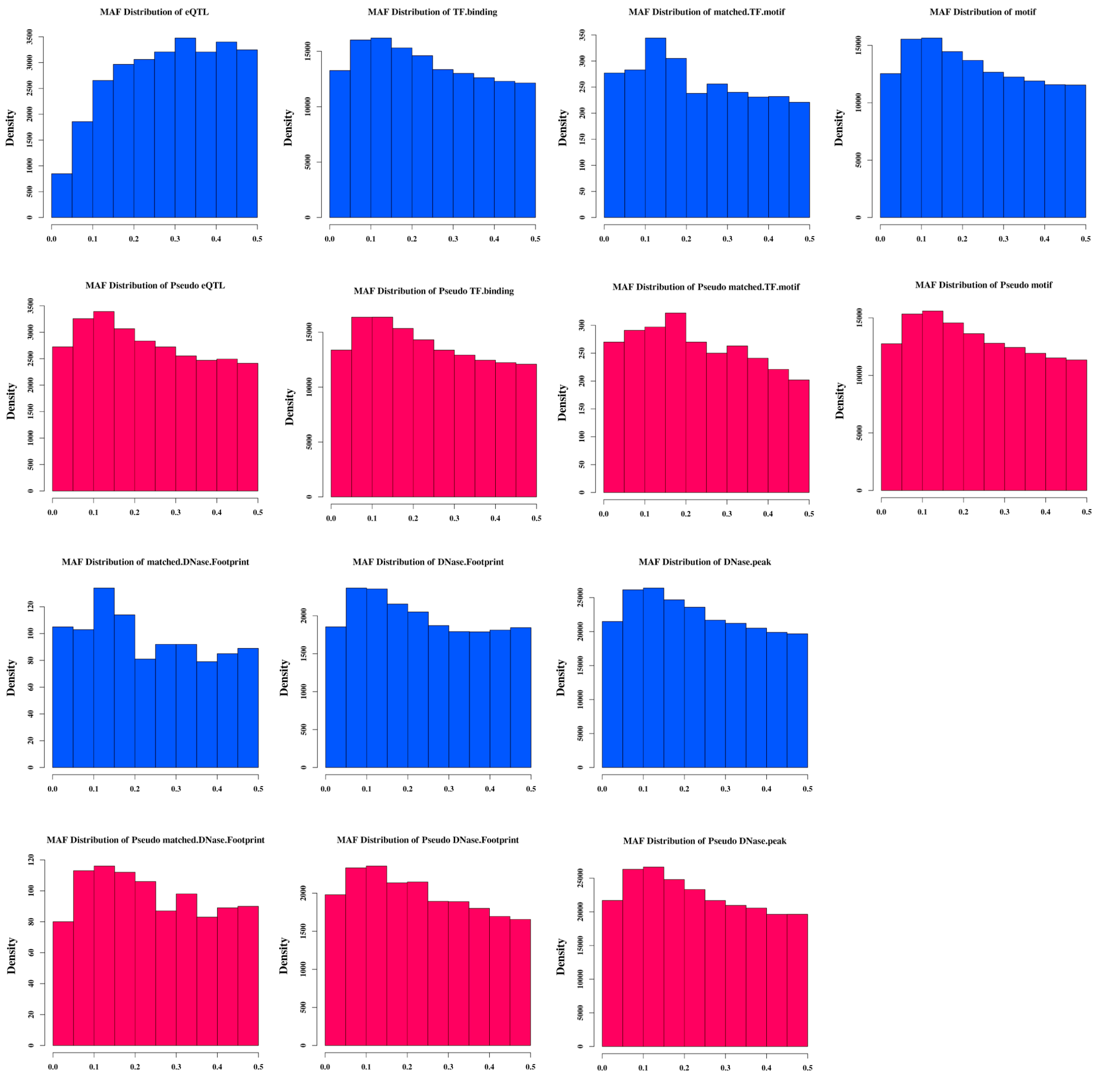

Supplementary Fig. 5. Distribution of Minor Allele Frequency (MAF) for real and pseudo annotations. The annotations are derived from the RegulomeDB database, which has 7 types. The MAF was calculated using data on bipolar disorder trait in the WTCCC1 dataset.

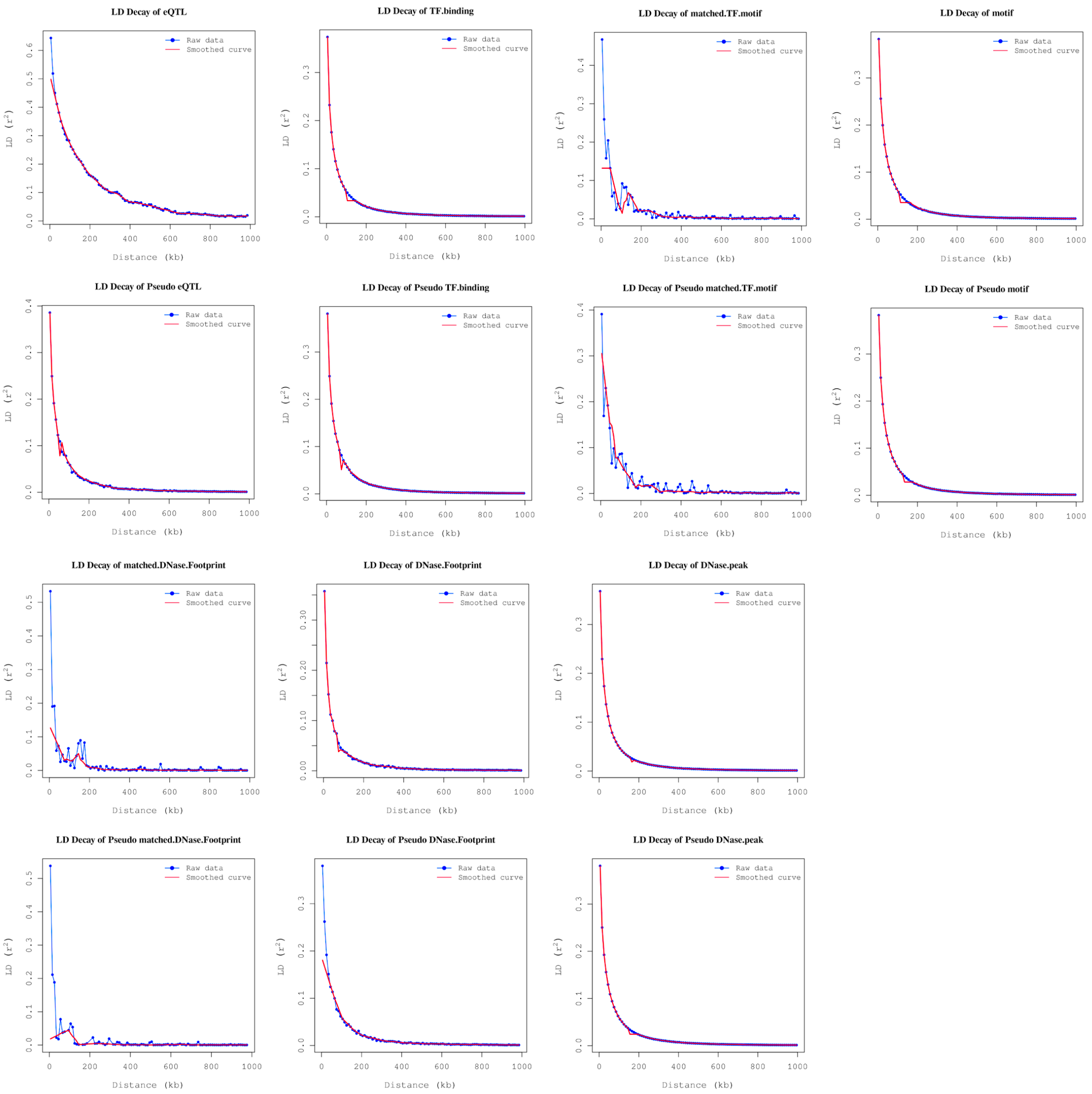

Supplementary Fig. 6. Distribution of Linkage Disequilibrium (LD) for real and pseudo annotations. The annotations are derived from the RegulomeDB database, which has 7 types. The LD was calculated using data on bipolar disorder trait in the WTCCC1 dataset.

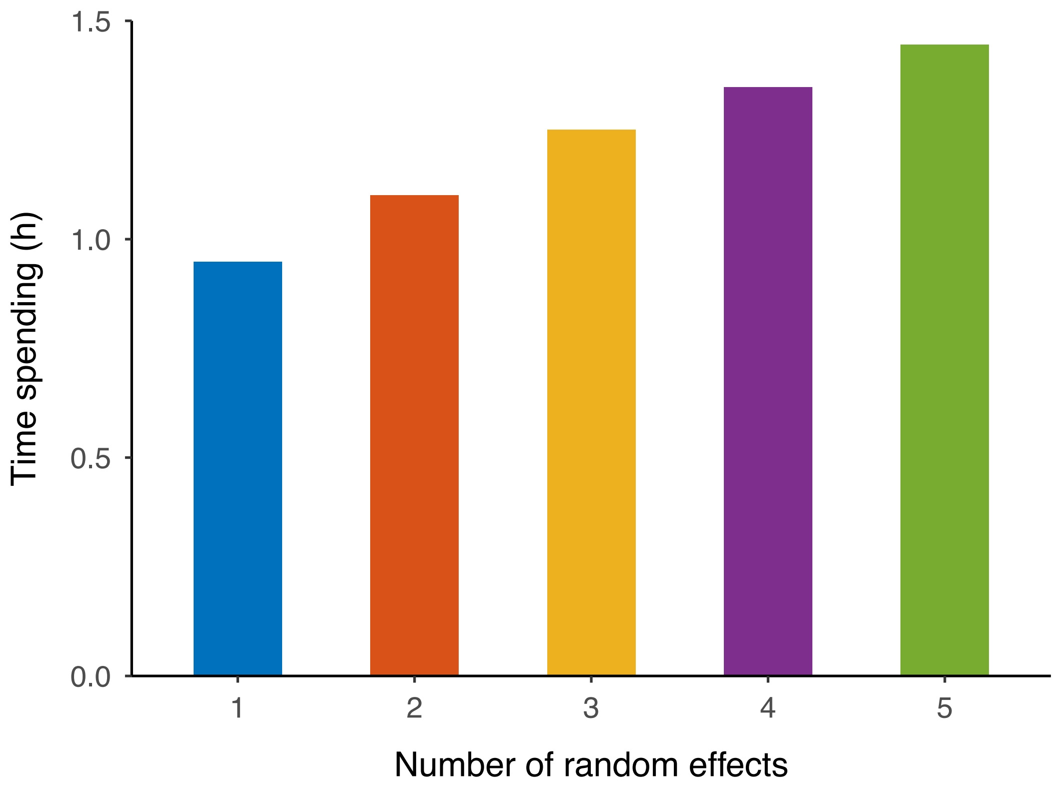

Supplementary Fig. 7. Comparisons of computational cost on time for different numbers of random effects within the IFAM model based on the "HE+PCG" strategy. The test was conducted once using the heel bone mineral density T-score (hBMD, N=181,028) trait from the UK Biobank dataset. Computational times were recorded for the estimation of the variance components using the Haseman–Elston (HE) regression algorithm and the genetics values using the Pre-conditioned Conjugate Gradient (PCG) iterative algorithm. All results were computed on a Linux server with 48 CPUs and 1,440 GB of memory.

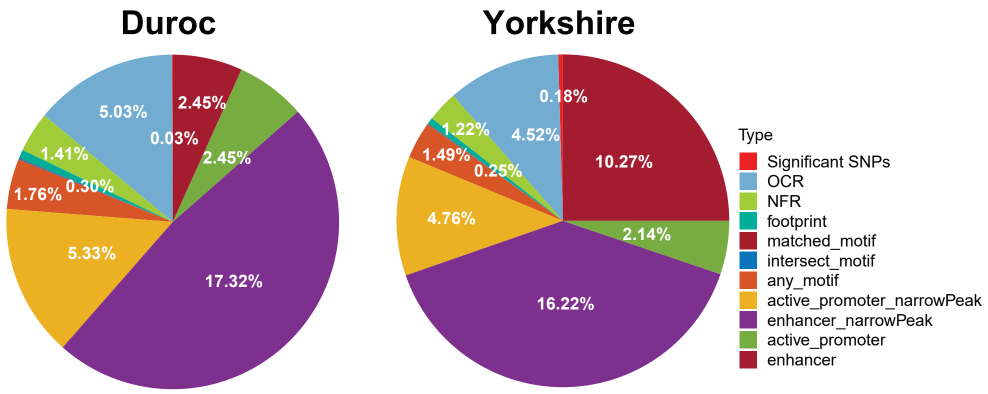

Supplementary Fig. 8. The average proportion of SNPs with distinct functional annotations within the Duroc pig (left) and Yorkshire pig (right) datasets. The annotation process utilized the 10 functional annotations from the IFmut database along with trait-specific significant SNPs generated from GWAS. See the Supplementary Table 33 for more details.

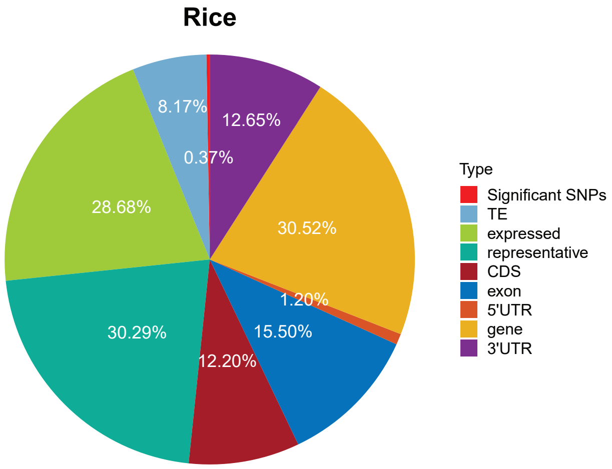

Supplementary Fig. 9. The average proportion of SNPs with distinct functional annotations within the rice datasets. The annotation process utilized the 9 functional annotations from the RGAP database along with trait-specific significant SNPs generated from GWAS. See the Supplementary Table 34 for more details.

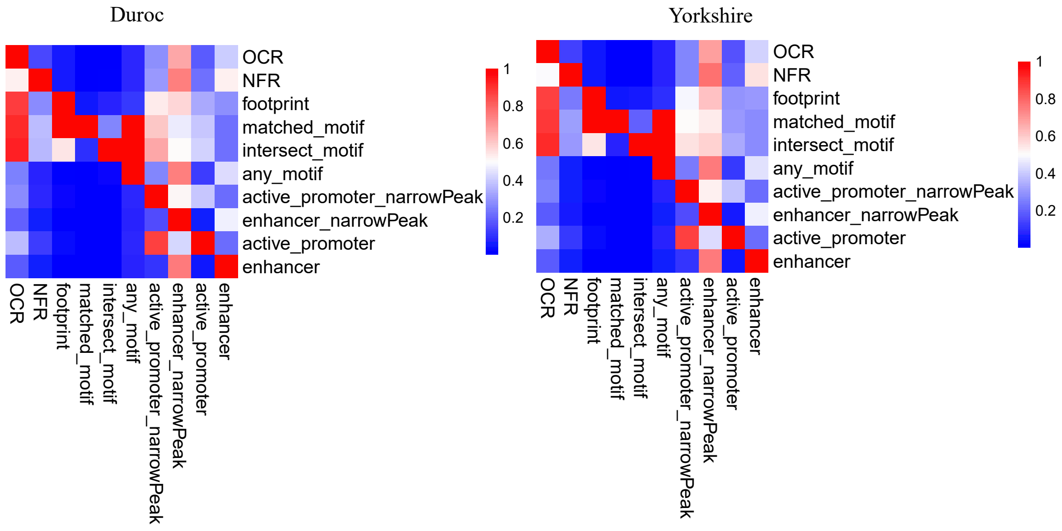

Supplementary Fig. 10. The heatmap of overlap ratio between functional annotations from the IFmut database based on the Duroc pig dataset and the Yorkshire pig dataset. The upper triangle represented the proportion of the number of SNPs overlapping A1 and A2 to A1, and the lower triangle represented the proportion of the number of SNPs overlapping A1 and A2 to A2.

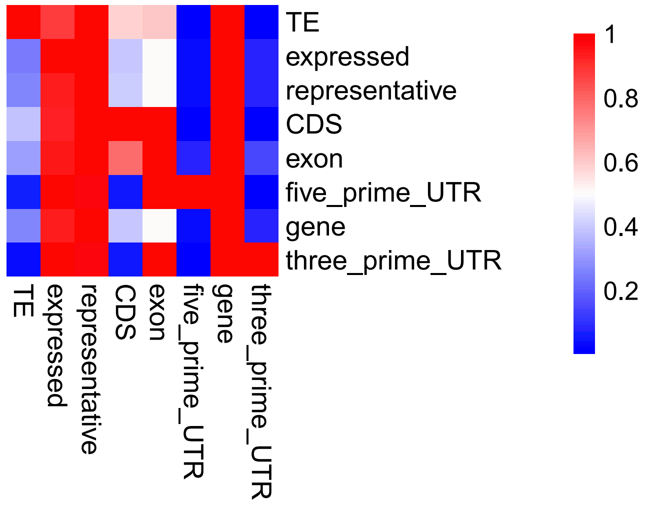

Supplementary Fig. 11. The heatmap of overlap ratio between functional annotations from the RGAP database based on the rice dataset. The upper triangle represented the proportion of the number of SNPs overlapping A1 and A2 to A1, and the lower triangle represented the proportion of the number of SNPs overlapping A1 and A2 to A2.

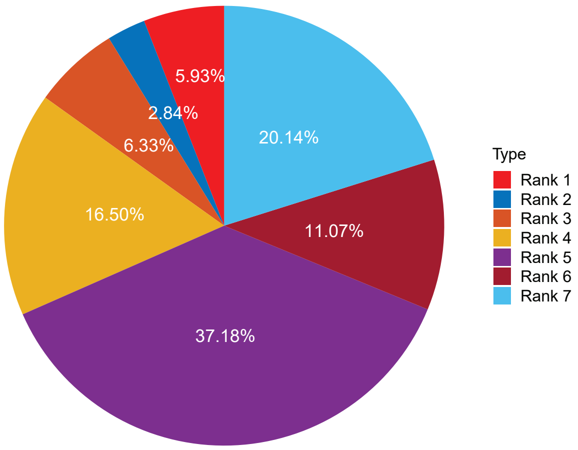

Supplementary Fig. 12. The average proportion of SNPs with distinct functional annotations across 7 traits within the WTCCC1 dataset. The annotation process utilized 7 ranking functional annotations from the RegulomeDB database. See the Supplementary Table 35 for more details.

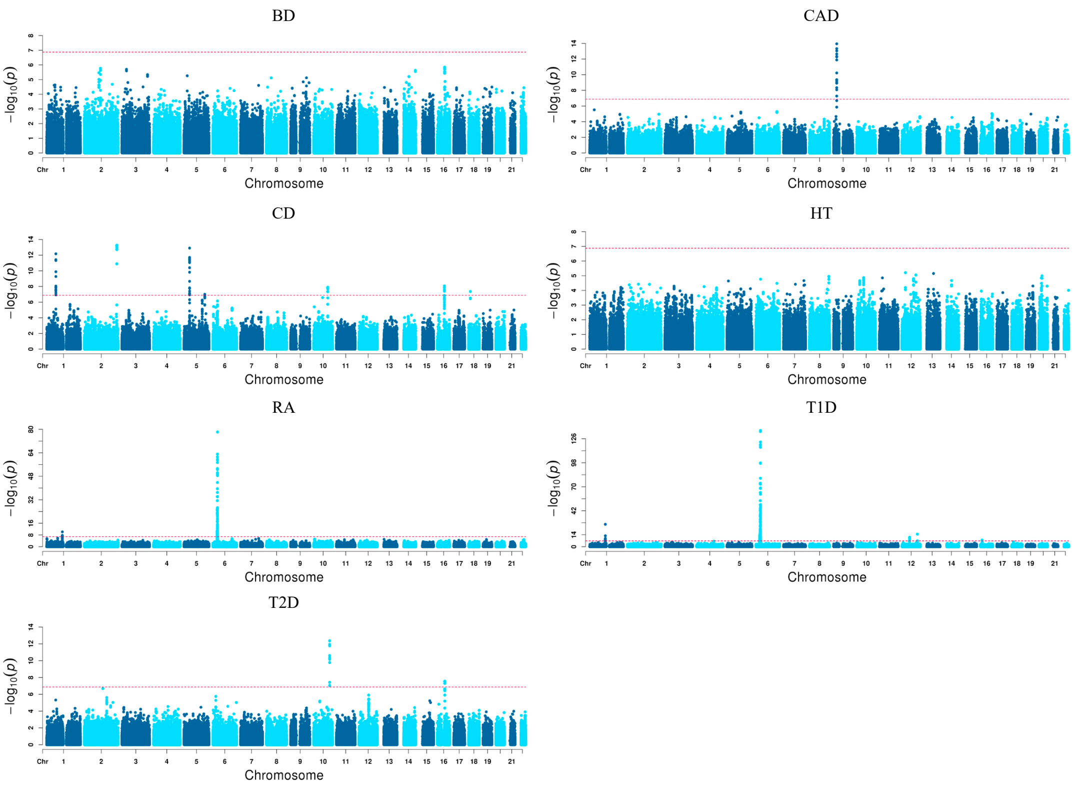

Supplementary Fig. 13. The Manhattan plots for bipolar disorder (BD), coronary artery disease (CAD), Crohn's disease (CD), hypertension (HT), rheumatoid arthritis (RA), type 1 diabetes (T1D), and type 2 diabetes (T2D) trait in the WTCCC1 dataset. The *P*-values were computed using the general linear model of the rMVP software (v1.0.8). The red line represents the threshold of significance, the X axis is the genomic coordinates of markers, and the Y axis is the negative logarithm of the association *P*-value for each SNP.

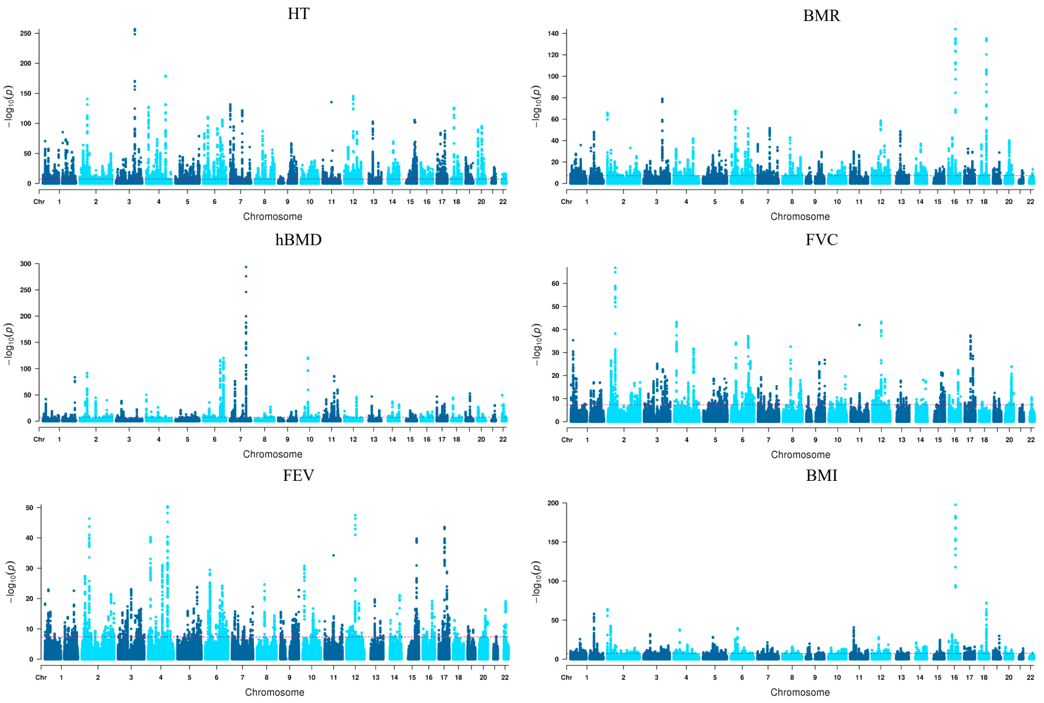

Supplementary Fig. 14. The Manhattan plots for height (HT), basal metabolic rate (BMR), heel bone mineral density T-score (hBMD), forced vital capacity (FVC), forced expiratory volume in 1 s (FEV), and body mass index (BMI) trait in the UK Biobank dataset. The *P*-values were calculated using the linear regression method of the 0PLINK software (v1.90). The red line represents the threshold of significance, the X axis is the genomic coordinates of markers, and the Y axis is the negative logarithm of the association *P*-value for each SNP.

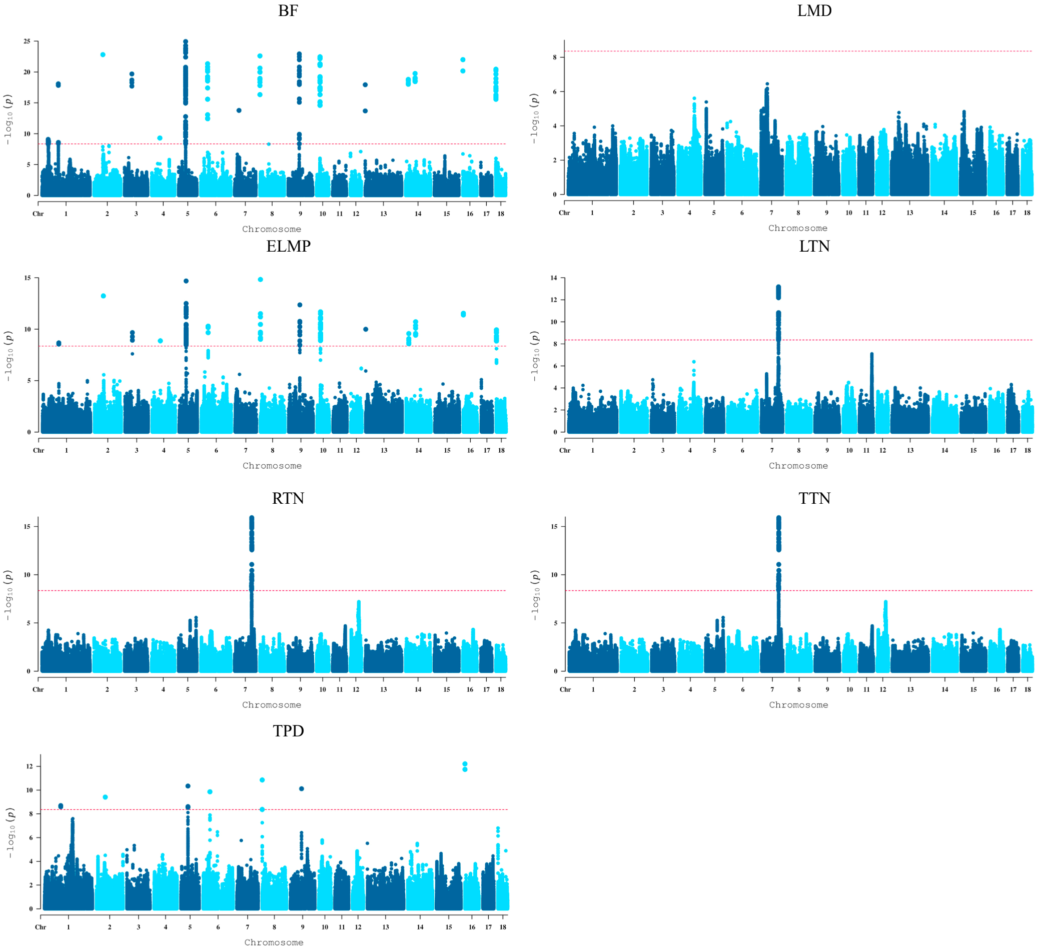

Supplementary Fig. 15. The Manhattan plots for backfat thickness (BF), loin muscle depth (LMD), estimated lean meat percentage (ELMP), left teat number (LTN), right teat number (RTN), total teat number (TTN), and time spent eating per day (TPD) trait in the Duroc pig dataset. The *P*-values were estimated using the mixed linear model of the rMVP software (v1.0.8). The red line represents the threshold of significance, the X axis is the genomic coordinates of markers, and the Y axis is the negative logarithm of the association *P*-value for each SNP.

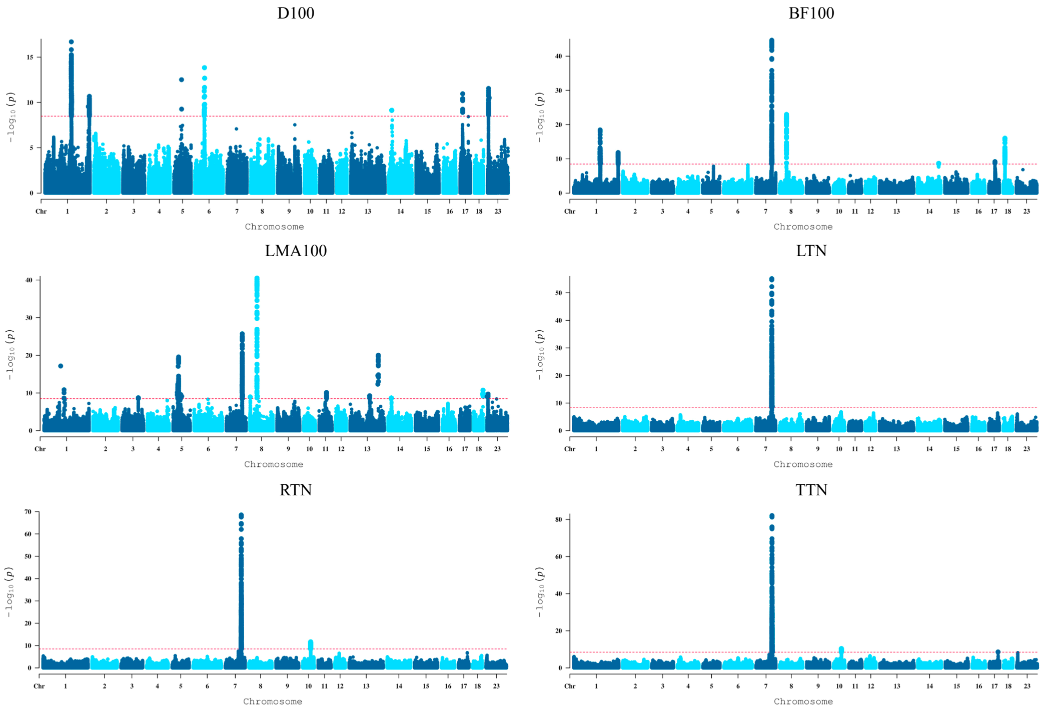

Supplementary Fig. 16. The Manhattan plots for age adjusted to 100 kg (D100), backfat thickness adjusted to 100 kg (BF100), loin muscle area adjusted to 100 kg (LMA100), left teat number (LTN), right teat number (RTN), and total teat number (TTN) trait in the Yorkshire pig dataset. The *P*-values were estimated using the mixed linear model of the rMVP software (v1.0.8). The red line represents the threshold of significance, the X axis is the genomic coordinates of markers, and the Y axis is the negative logarithm of the association *P*-value for each SNP.

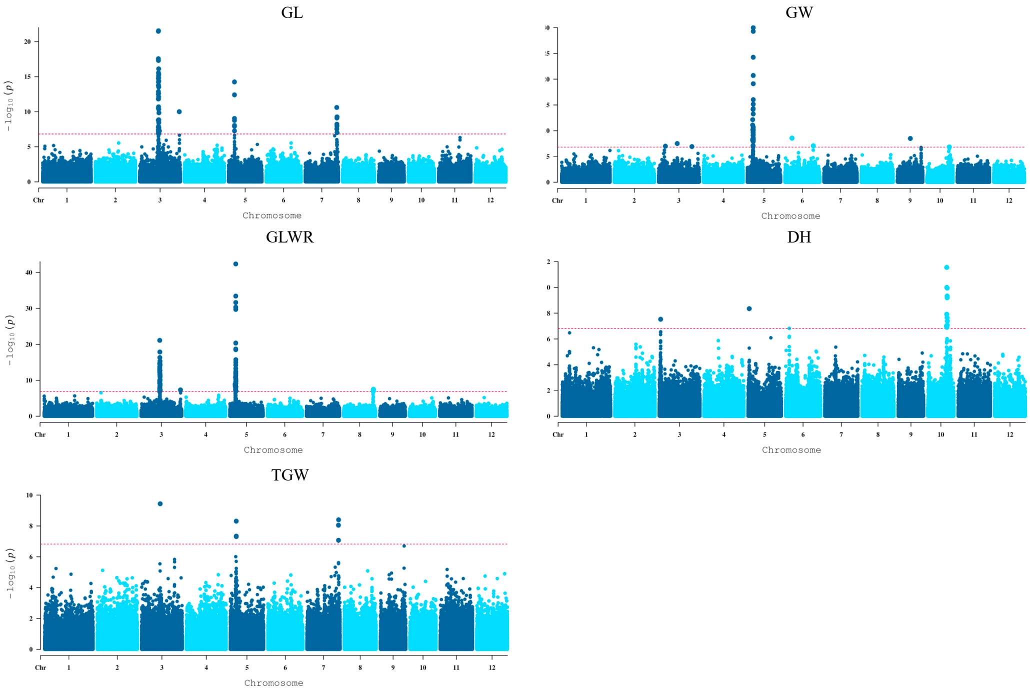

Supplementary Fig. 17. The Manhattan plots for grain length (GL), grain width (GW), grain length-to-width ratio (GLWR), days to heading (DH), and thousand grain weight (TGW) trait in the rice dataset. The *P*-values were estimated using the mixed linear model of the rMVP software (v1.0.8). The red line represents the threshold of significance, the X axis is the genomic coordinates of markers, and the Y axis is the negative logarithm of the association *P*-value for each SNP

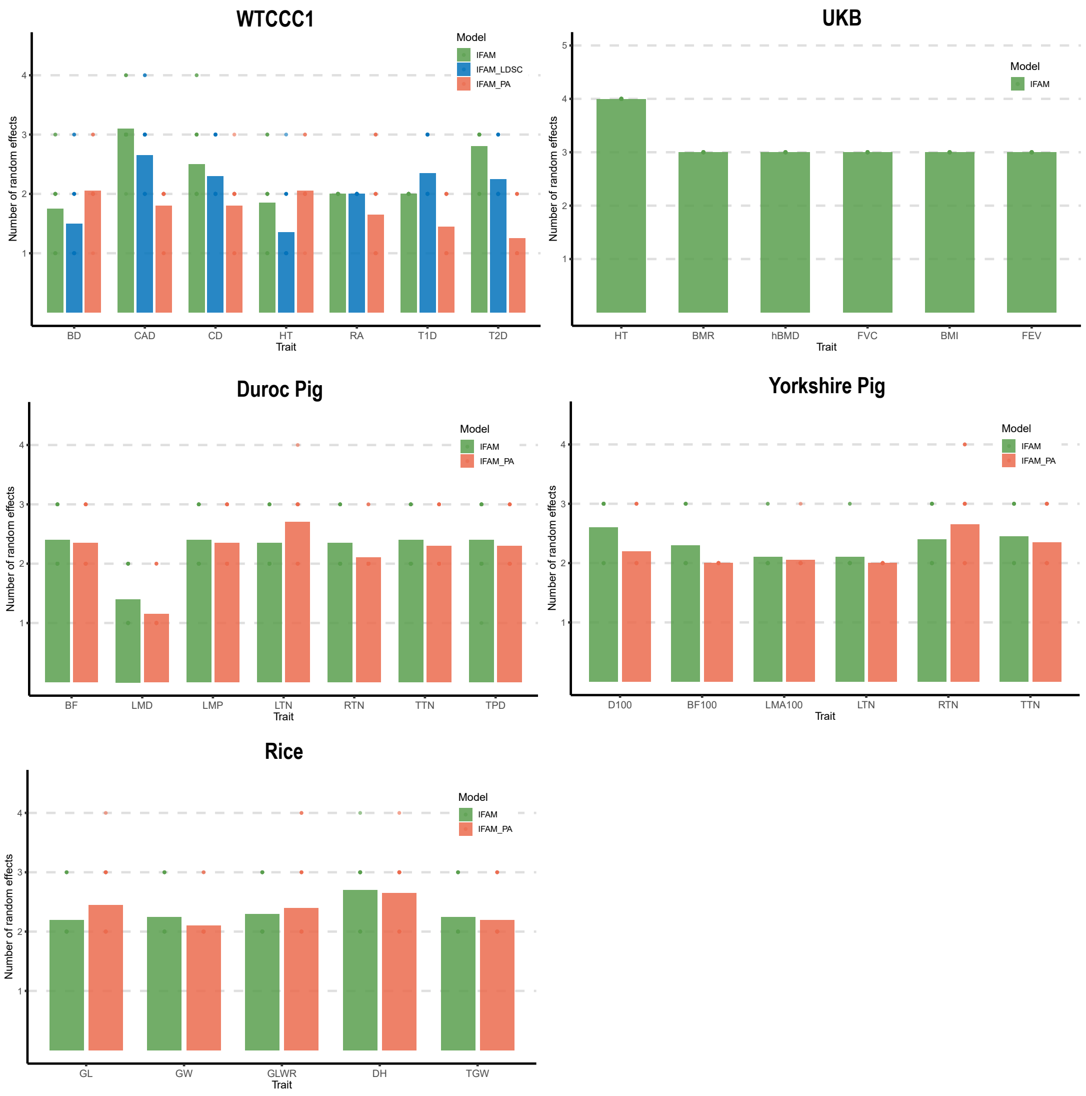

Supplementary Fig. 18. The number of random effects of the IFAM model under five datasets. The IFAM represents running the IFAM model using trait-specific significant SNPs generated from GWAS and the functional annotations derived from the RegulomeDB database for human datasets, those from the IFmut database for pig datasets, and those from the RGAP database for rice datasets. The IFAM_PA represents running the IFAM model using pseudo annotation information, which is selected randomly from the genome based on the scale of true annotations from the respective database. The IFAM_LDSC represents running the IFAM model using trait-specific significant SNPs generated from GWAS and the functional annotations derived from the website of the linkage disequilibrium score regression (LDSC) model in the WTCCC1 dataset. For the UK Biobank (UKB) dataset, we performed 10 times 80%/20% random splits for each trait and 20 times 80%/20% random splits for other datasets.

Supplementary Table 1. Examples of the importance assessment of functional annotation information in the WTCCC1 dataset and the criterion of assessment is the variance component. The functional annotation information was the ranking scores of SNP from the RegulomeDB database. The average information restricted maximum likelihood (AI-REML) algorithm was chosen to estimate the variance component. The standard errors of the estimates are shown in parentheses.

Category	BD[1]	CAD	CD	HT	RA	T1D	T2D	Mean
Rank 1	3.21E-06 (0.0048)	2.90E-06 (0.0049)	4.82E-06 (0.0052)	3.04E-06 (0.0050)	5.45E-06 (0.0050)	8.62E-06 (0.0051)	2.89E-06 (0.0049)	4.42E-06
Rank 2	2.38E-06 (0.0065)	1.01E-06 (0.0064)	2.21E-06 (0.0065)	1.33E-06 (0.0066)	2.42E-06 (0.0066)	9.96E-07 (0.0058)	9.55E-07 (0.0066)	1.61E-06
Rank 3	4.24E-07 (0.0094)	1.59E-06 (0.0096)	8.96E-07 (0.0092)	5.28E-07 (0.0093)	1.07E-06 (0.0094)	1.01E-06 (0.0084)	8.38E-07 (0.0093)	9.07E-07
Rank 4	5.96E-07 (0.0132)	8.48E-07 (0.0134)	1.51E-06 (0.0134)	6.72E-07 (0.0132)	1.04E-06 (0.0127)	9.29E-07 (0.0122)	1.74E-06 (0.0136)	1.05E-06
Rank 5	1.34E-06 (0.0179)	6.98E-07 (0.0181)	6.45E-07 (0.0172)	3.53E-07 (0.0178)	--	2.04E-07 (0.0163)	3.76E-07 (0.0175)	6.03E-07
Rank 6	4.32E-07 (0.0115)	1.77E-07 (0.0113)	--	4.94E-07 (0.0118)	1.27E-06 (0.0113)	--	--	5.94E-07
Rank 7	4.53E-07 (0.0138)	--[2]	7.56E-08 (0.0134)	5.57E-07 (0.0139)	7.96E-07 (0.0140)	8.21E-07 (0.0130)	2.87E-07 (0.0136)	4.98E-07

[1] BD: bipolar disorder; CAD: coronary artery disease; CD: Crohn's disease; HT: hypertension; RA: rheumatoid arthritis; T1D: type 1 diabetes; T2D: type 2 diabetes;

[2] "--": a missing value which is anomalous

Supplementary Table 2. The importance assessment of functional annotations from the RegulomeDB database in the WTCCC1 dataset and the criterion of assessment is the variance component. The average information restricted maximum likelihood (AI-REML) algorithm was chosen to estimate the variance component. The standard errors of the estimates are shown in parentheses.

Type of annotation	BD[1]	CAD	CD	HT	RA	T1D	T2D	Mean
eQTL	5.76E-07 (0.0053)	6.30E-07 (0.0052)	3.13E-06 (0.0054)	1.19E-06 (0.0054)	5.04E-06 (0.0052)	6.50E-06 (0.0052)	1.95E-06 (0.0053)	2.72E-06
TF binding	8.49E-07 (0.0258)	8.87E-07 (0.0259)	1.02E-06 (0.0222)	4.75E-07 (0.0256)	8.24E-07 (0.0217)	5.67E-07 (0.0210)	5.77E-07 (0.0226)	7.42E-07
matched TF motif	1.14E-06 (0.0043)	6.66E-07 (0.0044)	7.31E-06 (0.0046)	1.26E-07 (0.0043)	7.58E-06 (0.0047)	7.40E-06 (0.0046)	5.28E-06 (0.0045)	4.21E-06
motif	5.60E-07 (0.0227)	2.87E-07 (0.0224)	--	5.53E-07 (0.0230)	6.04E-07 (0.0186)	--	3.03E-08 (0.0201)	2.91E-07
matched DNase footprint	3.67E-07 (0.0027)	2.38E-07 (0.0027)	3.09E-06 (0.0030)	1.52E-06 (0.0026)	1.24E-05 (0.0037)	1.02E-05 (0.0036)	--	3.98E-06
DNase footprint	1.03E-06 (0.0097)	1.15E-07 (0.0094)	1.09E-06 (0.0085)	3.80E-07 (0.0094)	1.44E-06 (0.0082)	1.54E-06 (0.0082)	1.62E-06 (0.0087)	1.03E-06
DNase peak	9.91E-07 (0.0323)	7.31E-07 (0.0326)	7.34E-07 (0.0277)	6.48E-07 (0.0327)	--	6.24E-07 (0.0265)	9.52E-07 (0.0286)	6.68E-07

[1] BD: bipolar disorder; CAD: coronary artery disease; CD: Crohn’s disease; HT: hypertension; RA: rheumatoid arthritis; T1D: type 1 diabetes; T2D: type 2 diabetes;

[2] “--”: a missing value which is anomalous

Supplementary Table 3. Prediction performance of different models in the WTCCC1 dataset. The IFAM represents running the IFAM model using trait-specific significant SNPs generated from GWAS and functional annotations derived from the RegulomeDB database. The IFAM_LDSC represents running the IFAM model using trait-specific significant SNPs generated from GWAS and functional annotations derived from the website of the linkage disequilibrium score regression (LDSC) model. The Multi2R represents a genomic prediction model consisting of two random effects, one is composed of trait-specific significant SNPs and the other is composed of remaining SNPs. The MultiBLUP represents a genomic prediction model that contained 8 random effects, each of which is constructed using 7 functional annotations from the RegulomeDB database and trait-specific significant SNPs. The MultiBLUP_LDSC represents a genomic prediction model that contained 75 random effects, each of which is constructed using 74 functional annotations from the LDSC model and trait-specific significant SNPs. The BayesR represents running the BayesR model with default parameters, where the MCMC chain length was set to 50,000. The BayesRC represents running the BayesRC model with default parameters using the same functional annotations as the IFAM. The IFAM_PA represents running the IFAM model using pseudo annotation information, which is selected randomly from the genome based on the scale of true annotations from the RegulomeDB database. The accuracy of genomic prediction was assessed by the area under the curve (AUC) statistic using 20 repetitions. Statistical significance between IFAM and competing models was assessed via the corrected resampled two-sided t-test. The mean AUC values and standard errors of each trait are shown.

Trait[1]	IFAM	IFAM_LDSC	GBLUP	Multi2R	MultiBLUP	MultiBLUP_LDSC	Adaptive MultiBLUP	BayesR	BayesRC	IFAM_PA
BD	0.6466 (0.0030)	0.6465 (0.0029)	0.6474 (0.0029)	0.6472 (0.0029)	0.6463 (0.0037)	0.6185 (0.0035)**	0.6530 (0.0092)	0.6528 (0.0037)	0.5261 (0.0043)**	0.6455 (0.0033)
CAD	0.6053 (0.0027)	0.6021 (0.0026)*	0.5845 (0.0028)**	0.6024 (0.0026)*	0.6054 (0.0034)	0.5921 (0.0034)*	0.5924 (0.0108)	0.5975 (0.0027)*	0.5178 (0.0042)**	0.5837 (0.0029)**
CD	0.6758 (0.0039)	0.6756 (0.0035)	0.6322 (0.0032)**	0.6750 (0.0036)	0.6785 (0.0029)	0.6530 (0.0033)*	0.6102 (0.0127)*	0.6708 (0.0024)	0.5371 (0.0047)**	0.6319 (0.0034)**
HT	0.5951 (0.0031)	0.5945 (0.0030)	0.5947 (0.0031)	0.5942 (0.0030)	0.5985 (0.0031)	0.5929 (0.0024)	0.6151 (0.0072)	0.5932 (0.0034)	0.5160 (0.0031)**	0.5923 (0.0032)
RA	0.7215 (0.0031)	0.7214 (0.0031)	0.6113 (0.0032)**	0.7215 (0.0031)	0.7142 (0.0036)	0.6608 (0.0035)**	0.7198 (0.0030)	0.7152 (0.0034)	0.5291 (0.0041)**	0.6100 (0.0032)**
T1D	0.8699 (0.0024)	0.8699 (0.0024)	0.6542 (0.0037)**	0.8699 (0.0024)	0.8633 (0.0025)*	0.7516 (0.0028)**	0.8616 (0.0020)**	0.8618 (0.0019)*	0.5510 (0.0043)**	0.6549 (0.0038)**
T2D	0.6156 (0.0028)	0.6143 (0.0027)	0.5968 (0.0024)**	0.6153 (0.0027)	0.6138 (0.0034)	0.6035 (0.0033)	0.6088 (0.0087)	0.6179 (0.0031)	0.5256 (0.0036)**	0.5959 (0.0024)**
Mean	0.6757	0.6749	0.6173	0.6751	0.6743	0.6389	0.6658	0.6727	0.5290	0.6163

[1] BD: bipolar disorder; CAD: coronary artery disease; CD: Crohn's disease; HT: hypertension; RA: rheumatoid arthritis; T1D: type 1 diabetes; T2D: type 2 diabetes;

* represents a significant difference ($P < 0.05$) in the prediction accuracy between the competing model and IFAM. When the competing model is IFAM_LDSC, CAD: t(19)=2.3571, P=0.0293, 95% CI [-0.0029, 0.0095]; When the competing model is Multi2R, CAD: t(19)=2.5070, P=0.0214, 95% CI [-0.0035, 0.0095]; When the competing model is MultiBLUP, T1D: t(19)=2.3290,

P=0.0310, 95% CI [0.0007, 0.0125]; When the competing model is MultiBLUP_LDSC, CAD: t(19)=2.3819, P=0.0278, 95% CI [0.0052, 0.0213], CD: t(19)=2.2838, P=0.0341, 95% CI [0.0126, 0.0328]; When the competing model is Adaptive MultiBLUP, CD: t(19)=2.3491, P=0.0298, 95% CI [0.0376, 0.0935]; When the competing model is BayesR, CAD: t(19)=1.8830, P=0.0751, 95% CI [0.0026, 0.0132], T1D: t(19)=1.9257, P=0.0692, 95% CI [0.0037, 0.0126];

** represents an extremely significant difference ($P < 0.01$) in the prediction accuracy between the competing model and IFAM. When the competing model is GBLUP, CAD: t(19)=3.6881, P=0.0016, 95% CI [0.0076, 0.0342], CD: t(19)=5.8631, P<1E-04, 95% CI [0.0080, 0.0791], RA: t(19)=16.6953, P<1E-04, 95% CI [-0.1045, 0.3248], T1D: t(19)=18.8057, P<1E-04, 95% CI [-0.2473, 0.6788], T2D: t(19)=4.0118, P=0.0007, 95% CI [0.0055, 0.0321]; When the competing model is MultiBLUP_LDSC, BD: t(19)=5.4007, P<1E-04, 95% CI [0.0037, 0.0524], RA: t(19)=9.6451, P<1E-04, 95% CI [-0.0127, 0.1341], T1D: t(19)=19.2080, P<1E-04, 95% CI [-0.1436, 0.3801]; When the competing model is Adaptive MultiBLUP, T1D: t(19)=3.0786, P=0.0068, 95% CI [0.0010, 0.0155]; When the competing model is BayesRC, BD: t(19)=9.0377, P<1E-04, 95% CI [-0.0071, 0.2481], CAD: t(19)=8.0177, P<1E-04, 95% CI [0.0034, 0.1718], CD: t(19)=11.3218, P<1E-04, 95% CI [-0.0455, 0.3228], HT: t(19)=10.2649, P<1E-04, 95% CI [-0.0181, 0.1763], RA: t(19)=15.8807, P<1E-04, 95% CI [-0.1582, 0.5429], T1D: t(19)=25.2058, P<1E-04, 95% CI [-0.5968, 1.2345], T2D: t(19)=7.1273, P<1E-04, 95% CI [0.0139, 0.1660]; When the competing model is IFAM_PA, CAD: t(19)=3.8038, P=0.0012, 95% CI [0.0077, 0.0356], CD: t(19)=5.5014, P<1E-04, 95% CI [0.0101, 0.0776], RA: t(19)=17.0667, P<1E-04, 95% CI [-0.1106, 0.3336], T1D: t(19)=18.5165, P<1E-04, 95% CI [-0.2394, 0.6693], T2D: t(19)=4.3249, P=0.0003, 95% CI [0.0049, 0.0343]

Supplementary Table 4. Prediction bias of different models in the WTCCC1 dataset.

The explanation of results is the same as that in the Supplementary Table 3.

Trait[1]	IFAM	IFAM_LDSC	GBLUP	Multi2R	MultiBLUP	MultiBLUP_LDSC	Adaptive MultiBLUP	BayesR	BayesRC	IFAM_PA
BD	1.0306 (0.0283)	1.0136 (0.0312)	1.0231 (0.0295)	1.0207 (0.0297)	0.9678 (0.0356)	0.5005 (0.0165)	0.8945 (0.0700)	1.0012 (0.0315)	0.0253 (0.0035)	1.0267 (0.0308)
CAD	0.8947 (0.0395)	0.9493 (0.0316)	0.9414 (0.0432)	0.9599 (0.0336)	0.8406 (0.0375)	0.3818 (0.0125)	0.4430 (0.0792)	0.9424 (0.0357)	0.0115 (0.0043)	0.8983 (0.0382)
CD	0.9401 (0.0218)	0.9492 (0.0211)	1.0119 (0.0324)	0.9505 (0.0218)	0.7996 (0.0166)	0.5995 (0.0122)	0.3889 (0.0730)	0.9877 (0.0221)	0.0337 (0.0033)	1.0164 (0.0325)
HT	0.9908 (0.0459)	0.9899 (0.0455)	1.0011 (0.0463)	0.9926 (0.0463)	0.8609 (0.0348)	0.3836 (0.0092)	0.8532 (0.0541)	0.9872 (0.0529)	0.0141 (0.0029)	0.9165 (0.0511)
RA	0.9844 (0.0193)	0.9844 (0.0193)	0.9657 (0.0362)	0.9844 (0.0193)	0.8720 (0.0224)	0.6112 (0.0120)	1.0024 (0.0198)	0.9993 (0.0257)	0.0284 (0.0035)	0.9678 (0.0367)
T1D	0.9894 (0.0095)	0.9894 (0.0095)	1.0612 (0.0335)	0.9894 (0.0095)	1.0191 (0.0102)	0.8576 (0.0108)	0.9943 (0.0084)	1.0002 (0.0090)	0.0464 (0.0036)	1.0654 (0.0338)
T2D	0.9831 (0.0325)	0.9982 (0.0318)	0.9846 (0.0320)	1.0032 (0.0328)	0.7454 (0.0334)	0.4331 (0.0147)	0.7142 (0.0871)	1.0011 (0.0365)	0.0222 (0.0035)	0.9723 (0.0362)
Mean	0.9733	0.9820	0.9984	0.9858	0.8722	0.5382	0.7558	0.9884	0.0259	0.9805

[1] BD: bipolar disorder; CAD: coronary artery disease; CD: Crohn's disease; HT: hypertension; RA: rheumatoid arthritis; T1D: type 1 diabetes; T2D: type 2 diabetes

Supplementary Table 5. Prediction performance of different scenarios of BayesRC in the WTCCC1 dataset using functional annotation from the RegulomeDB database. The BayesRC represents running the BayesRC using the default parameters, where the MCMC chain length (Numit) was set to 50,000, the burnin steps were set to 20,000, and the functional annotations are the same as the IFAM, which are the trait-specific significant SNPs generated from GWAS and the functional annotations derived from the RegulomeDB database. The BayesRC_opt1 represents running the BayesRC using the alternative parameters, where the MCMC chain length (Numit) was set to 100,000, the burnin steps were set to 40,000, and the functional annotations are the same as the BayesRC. The BayesRC_opt2 represents running the BayesRC using the alternative parameters, where the MCMC chain length (Numit) was set to 50,000, the burnin steps were set to 20,000, and the functional annotations are eQTL and matched TF motif. Statistical significance between IFAM and competing models was assessed via the corrected resampled two-sided t-test.

Trait[1]	BayesRC<br>Numit=50K	BayesRC_opt1<br>Numit=100K	BayesRC_opt2<br>Two annotations
BD	0.5261(0.0043)	0.5261(0.0041)	0.5583(0.0042)
CAD	0.5178(0.0042)	0.5189(0.0036)	0.5378(0.0048)
CD	0.5371(0.0047)	0.5384(0.0046)	0.5821(0.0043)**
HT	0.5160(0.0031)	0.5166(0.0033)	0.5438(0.0037)*
RA	0.5291(0.0041)	0.5301(0.0040)	0.6581(0.0036)**
T1D	0.5510(0.0043)	0.5536(0.0044)	0.7828(0.0024)**
T2D	0.5256(0.0036)	0.5247(0.0037)	0.5582(0.0037)*
Mean	0.5290	0.5298	0.6030

[1] BD: bipolar disorder; CAD: coronary artery disease; CD: Crohn's disease; HT: hypertension; RA: rheumatoid arthritis; T1D: type 1 diabetes; T2D: type 2 diabetes;

[*] represents a significant difference ($P < 0.05$) in the prediction accuracy between the competing scenario and BayesRC. When the competing model is BayesRC_opt2, HT: t(19)=-2.1925, P=0.0410, 95% CI [-0.0378, -0.0176], T2D: t(19)=-2.5567, P=0.0193, 95% CI [-0.0457, -0.0193];

[**] represents an extremely significant difference ($P < 0.01$) in the prediction accuracy between the competing scenario and BayesRC. When the competing model is BayesRC_opt2, CD: t(19)=-3.1467, P=0.0053, 95% CI [-0.0662, -0.0238], RA: t(19)=-8.8165, P<1E-04, 95% CI [-0.2620, 0.0041], T1D: t(19)=-20.3722, P<1E-04, 95% CI [-0.7709, 0.3073]

Supplementary Table 6. Prediction performance of different models in the UKB dataset. The IFAM represents running the IFAM model the trait-specific significant SNPs generated from GWAS and functional annotations derived from the RegulomeDB database. The Multi2R represents a genomic prediction model consisting of two random effects, one is composed of trait-specific significant SNPs and the other is composed of remaining SNPs. The MultiBLUP represents a genomic prediction model that contained 8 random effects, each of which is constructed using 7 functional annotations from the RegulomeDB database and trait-specific significant SNPs. The BayesR represents running the BayesR model with default parameters, where the MCMC chain length was set to 10,000. The accuracy of genomic prediction was assessed by the correlation between estimated genetic values and phenotypic values using 10 repetitions. Statistical significance between IFAM and competing models was assessed via the corrected resampled two-sided t-test. The mean correlation values and standard errors of each trait are shown.

**Trait[1]**	**IFAM**	**GBLUP**	**Multi2R**	**MultiBLUP**	**BayesR**
HT	0.5943(0.0012)	0.5629(0.0008)**	0.5785(0.0022)*	0.1532(0.0714)**	0.6031(0.0008)**
BMR	0.4064(0.0007)	0.3930(0.0009)**	0.4022(0.0008)**	0.3574(0.0272)	0.4016(0.0007)**
hBMD	0.3904(0.0013)	0.3293(0.0012)**	0.3832(0.0013)**	0.1724(0.0205)**	0.4061(0.0013)**
FVC	0.2983(0.0025)	0.2854(0.0023)**	0.2966(0.0025)**	0.3013(0.0025)**	0.2990(0.0022)
BMI	0.3448(0.0012)	0.3407(0.0010)**	0.3444(0.0012)*	0.3415(0.0029)	0.3389(0.0009)**
FEV	0.3029(0.0010)	0.2903(0.0011)**	0.3013(0.0010)**	0.3054(0.0011)**	0.3031(0.0009)
Mean	0.3895	0.3669	0.3844	0.2719	0.3920

[1] HT: height; BMR: basal metabolic rate; hBMD: heel bone mineral density T-score; FVC: forced vital capacity; FEV: forced expiratory volume in 1s; BMI: body mass index;

* represents a significant difference ($P < 0.05$) in the prediction accuracy between the competing model and IFAM. When the competing model is Multi2R, HT: t(9)=2.7668, P=0.0219, 95% CI [0.0072, 0.0244], BMI: t(9)=3.1466, P=0.0118, 95% CI [-0.0033, 0.0042];

** represents an extremely significant difference ($P < 0.01$) in the prediction accuracy between the competing model and IFAM. When the competing model is GBLUP, HT: t(9)=31.4739, P<1E-04, 95% CI [-0.1322, 0.1950], BMR: t(9)=29.6557, P<1E-04, 95% CI [-0.0551, 0.0819], hBMD: t(9)=41.8189, P<1E-04, 95% CI [-0.3564, 0.4785], FVC: t(9)=7.0477, P<1E-04, 95% CI [-0.0091,

0.0348], BMI: t(9)=4.8788, P=0.0009, 95% CI [-0.0021, 0.0103], FEV: t(9)=34.8021, P<1E-04, 95% CI [-0.0672, 0.0925]; When the competing model is Multi2R, BMR: t(9)=10.4348, P<1E-04, 95% CI [-0.0064, 0.0148], hBMD: t(9)=9.9420, P<1E-04, 95% CI [-0.0100, 0.0244], FVC: t(9)=7.0477, P<1E-04, 95% CI [-0.0155, 0.0188], FEV: t(9)=10.9458, P<1E-04, 95% CI [-0.0096, 0.0128]; When the competing model is MultiBLUP, HT: t(9)=3.3470, P=0.0086, 95% CI [0.1505, 0.7316], hBMD: t(9)=5.5431, P=0.0004, 95% CI [0.0069, 0.4292], FVC: t(9)=-7.9893, P<1E-04, 95% CI [-0.0230, 0.0168], FEV: t(9)=-8.5897, P<1E-04, 95% CI [-0.0120, 0.0070]; When the competing model is BayesR, HT: t(9)=-7.3822, P<1E-04, 95% CI [-0.0215, 0.0040], BMR: t(9)=4.5916, P=0.0013, 95% CI [0.0000, 0.0096], hBMD: t(9)=-22.7799, P<1E-04, 95% CI [-0.0808, 0.0493], BMI: t(9)=5.8346, P=0.0002, 95% CI [-0.0023, 0.0141]

Supplementary Table 7. Prediction bias of different models in the UKB dataset. The explanation of results is the same as that in the Supplementary Table 6.

Trait[1]	IFAM	GBLUP	Multi2R	MultiBLUP	BayesR
HT	0.9188(0.0042)	1.0297(0.0023)	1.0650(0.0048)	0.2125(0.1332)	0.9731(0.0018)
BMR	1.0101(0.0041)	1.0635(0.0037)	1.0700(0.0027)	0.8655(0.0954)	0.9484(0.0024)
hBMD	0.9386(0.0058)	1.0832(0.0057)	1.0574(0.0060)	0.2291(0.0485)	0.9955(0.0048)
FVC	1.0170(0.0060)	1.0361(0.0045)	1.0313(0.0048)	1.0204(0.0051)	0.9553(0.0031)
BMI	1.0388(0.0055)	1.0388(0.0047)	1.0447(0.0048)	1.0153(0.0171)	0.9397(0.0043)
FEV	0.9999(0.0061)	1.0233(0.0040)	1.0238(0.0035)	1.0104(0.0032)	0.9460(0.0029)
Mean	0.9864	1.0457	1.0487	0.7255	0.9597

[1] HT: height; BMR: basal metabolic rate; hBMD: heel bone mineral density T-score; FVC: forced vital capacity; FEV: forced expiratory volume in 1s; BMI: body mass index

Supplementary Table 8. The estimates of genetic variance component were computed by GBLUP, IFAM, and a genomic prediction model that contains multiple random effects (MultiBLUP) using the UK Biobank dataset. The MultiBLUP model contained 8 random effects, each of which was constructed using 7 functional annotations from the RegulomeDB database and significant SNPs of trait identified through GWAS. The standard errors of the estimates are shown in parentheses.

Trait[1]	HT	BMR	hBMD	FVC	BMI	FEV
GBLUP	0.4630(0.0005)	0.2635(0.0006)	0.2420(0.0006)	0.1779(0.0003)	0.2246(0.0004)	0.1910(0.0004)
IFAM[2]	0.6108(0.0043)	0.2728(0.0024)	0.3118(0.0026)	0.1665(0.0020)	0.2058(0.0006)	0.1830(0.0028)
MultiBLUP[3]	0.3911(0.0147)	0.2366(0.0005)	0.2140(0.0012)	0.1656(0.0005)	0.2058(0.0002)	0.1779(0.0006)
MultiBLUP-1	-0.0414(0.0087)	-0.0134(0.0004)	-0.0223(0.0004)	-0.0031(0.0005)	-0.0085(0.0005)	-0.0051(0.0005)
MultiBLUP-2	0.2087(0.0195)	0.0722(0.0020)	0.1649(0.0021)	0.0688(0.0017)	0.0365(0.0015)	0.0724(0.0012)
MultiBLUP-3	0.0023(0.0007)	0.0057(0.0003)	0.0072(0.0007)	-0.0001(0.0005)	0.0000(0.0003)	0.0003(0.0003)
MultiBLUP-4	-0.0446(0.0039)	0.0232(0.0015)	-0.0294(0.0019)	0.0008(0.0015)	0.0504(0.0014)	0.0065(0.0011)
MultiBLUP-5	-0.0014(0.0006)	-0.0030(0.0002)	-0.0038(0.0004)	-0.0004(0.0003)	-0.0017(0.0002)	0.0007(0.0002)
MultiBLUP-6	0.0401(0.0030)	0.0170(0.0007)	-0.0008(0.0011)	0.0119(0.0007)	0.0098(0.0004)	0.0162(0.0008)
MultiBLUP-7	0.0842(0.0097)	0.0783(0.0021)	0.0085(0.0029)	0.0629(0.0019)	0.0908(0.0019)	0.0607(0.0013)
MultiBLUP-8	0.1783(0.0009)	0.0566(0.0005)	0.0896(0.0006)	0.0249(0.0003)	0.0284(0.0004)	0.0263(0.0003)

[1] HT: height; BMR: basal metabolic rate; hBMD: heel bone mineral density T-score; FVC: forced vital capacity; FEV: forced expiratory volume in 1s; BMI: body mass index;

[2] IFAM: the sum of genetic variance component estimates of optimized random effect in the IFAM model;

[3] MultiBLUP: the sum of genetic variance component estimates of many random effects in the MultiBLUP model

Supplementary Table 9. Prediction performance of different models in the Duroc pig dataset. The IFAM represents running the IFAM model using trait-specific significant SNPs generated from GWAS and functional annotations derived from the IFmut database. The Multi2R represents a genomic prediction model consisting of two random effects, one is composed of trait-specific significant SNPs and the other is composed of remaining SNPs. The MultiBLUP represents a genomic prediction model that contained 11 random effects, each of which is constructed using 10 functional annotations from the IFmut database and trait-specific significant SNPs. The BayesR represents running the BayesR model with the parameters, where the MCMC chain length was set to 10,000. The IFAM_PA represents running the IFAM model using pseudo annotation information, which is selected randomly from the genome based on the scale of true annotations from the IFmut database. The accuracy of genomic prediction was assessed by the correlation between estimated genetic values and phenotypic values using 20 repetitions. Statistical significance between IFAM and competing models was assessed via the corrected resampled two-sided t-test. The mean correlation values and standard errors of each trait are shown.

Trait[1]	IFAM	GBLUP	Multi2R	MultiBLUP	Adaptive MultiBLUP	BayesR	IFAM_PA
BF	0.4303 (0.0076)	0.3988 (0.0084)**	0.4304 (0.0076)	0.3779 (0.0079)**	0.1987 (0.0337)**	0.0014 (0.0096)**	0.3864 (0.0076)**
LMD	0.3771 (0.0050)	0.376 3(0.0051)	0.3763 (0.0051)	0.3386 (0.0063)**	0.2201 (0.0266)*	0.0378 (0.0096)**	0.3755 (0.0053)
ELMP	0.4883 (0.0075)	0.4392 (0.0075)*	0.4485 (0.0075)	0.4095 (0.0074)**	0.1955 (0.0256)**	0.0088 (0.0109)**	0.4348 (0.0070)*
LTN	0.3420 (0.0051)	0.3210 (0.0070)	0.3431 (0.0052)	0.2535 (0.0084)**	0.3333 (0.0105)	0.0018 (0.0082)**	0.2977 (0.0083)*
RTN	0.2905 (0.0066)	0.2638 (0.0077)**	0.2894 (0.0066)	0.2307 (0.0066)**	0.2724 (0.0086)	0.0059 (0.0100)**	0.2549 (0.0082)**
TTN	0.4167 (0.0069)	0.3900 (0.0072)**	0.4164 (0.0072)	0.3641 (0.0069)**	0.3973 (0.0106)	0.0119 (0.0091)**	0.3852 (0.0077)**
TPD	0.4688 (0.0068)	0.4628 (0.0069)	0.4695 (0.0067)	0.4169 (0.0065)**	0.2006 (0.0311)**	0.4756 (0.0067)	0.4556 (0.0077)
Mean	0.3962	0.3788	0.3962	0.3416	0.2597	0.0776	0.3700

[1] BF: backfat thickness; LMD: loin muscle depth; ELMP: estimated lean meat percentage; LTN: left teat number; RTN: right teat number; TTN: total teat number; TPD: time spent eating per day;
* represents a significant difference ($P < 0.05$) in the prediction accuracy between the competing model and IFAM. When the competing model is GBLUP, ELMP: t(19)=2.6205, P=0.0168, 95% CI [-0.0130, 0.0311]; When the competing model is Adaptive MultiBLUP, LMD: t(19)=2.5667,

P=0.0189, 95% CI [0.0905, 0.2236]; When the competing model is IFAM_PA, ELMP: t(19)=2.1905, P=0.0412, 95% CI [-0.0025, 0.0295], LTN: t(19)=2.4683, P=0.0232, 95% CI [0.0234, 0.0651];

** represents an extremely significant difference ($P < 0.01$) in the prediction accuracy between the competing model and IFAM. When the competing model is GBLUP, BF: t(19)=3.0713, P=0.0063, 95% CI [0.0048, 0.0582], RTN: t(19)=4.3431, P=0.0004, 95% CI [-0.0068, 0.0602], TTN: t(19)=4.3087, P=0.0004, 95% CI [-0.0324, 0.0594]; When the competing model is MultiBLUP, BF: t(19)=3.7436, P=0.0014, 95% CI [0.0161, 0.0888], LMD: t(19)=2.9496, P=0.0082, 95% CI [0.0175, 0.0596], ELMP: t(19)=3.2379, P=0.0043, 95% CI [0.0109, 0.0666], LTN: t(19)=4.9380, P<1E-04, 95% CI [0.0285, 0.1484], RTN: t(19)=5.0896, P<1E-04, 95% CI [0.0119, 0.1078], TTN: t(19)=5.7762, P<1E-04, 95% CI [0.0002, 0.1051], TPD: t(19)=4.3265, P=0.0004, 95% CI [0.0137, 0.0900]; When the competing model is Adaptive MultiBLUP, BF: t(19)=2.9812, P=0.0077, 95% CI [0.1254, 0.3379], ELMP: t(19)=4.2704, P=0.0004, 95% CI [0.1069, 0.3986], TPD: t(19)=4.3265, P=0.0045, 95% CI [0.1472, 0.3891]; When the competing model is BayesR, BF: t(19)=14.0720, P<1E-04, 95% CI [-0.2651, 1.1230], LMD: t(19)=12.6432, P<1E-04, 95% CI [-0.1557, 0.8344], ELMP: t(19)=15.6013, P<1E-04, 95% CI [-0.3501, 1.2290], LTN: t(19)=15.3056, P<1E-04, 95% CI [-0.2582, 0.9384], RTN: t(19)=9.4384, P<1E-04, 95% CI [-0.0296, 0.5989], TTN: t(19)=13.0613, P<1E-04, 95% CI [-0.2029, 1.0127]; When the competing model is IFAM_PA, BF: t(19)=4.1277, P=0.0006, 95% CI [0.0066, 0.0813], RTN: t(19)=4.6786, P=0.0002, 95% CI [-0.0037, 0.0750], TTN: t(19)=3.5286, P=0.0022, 95% CI [0.0030, 0.0600]

Supplementary Table 10. Prediction bias of different models in the Duroc pig dataset. The explanation of results is the same as that in the Supplementary Table 9.

Trait[1]	IFAM	GBLUP	Multi2R	MultiBLUP	Adaptive MultiBLUP	BayesR	IFAM_PA
BF	1.0104 (0.0235)	1.0340 (0.0285)	1.0128 (0.0232)	0.5406 (0.0126)	0.2976 (0.0616)	0.0000 (0.0002)	0.7651 (0.0275)
LMD	1.0017 (0.0237)	1.0019 (0.0237)	1.0019 (0.0237)	0.4932 (0.0118)	0.4427 (0.0820)	0.0038 (0.0011)	0.9656 (0.0335)
ELMP	1.0377 (0.0280)	1.0544 (0.0277)	1.0450 (0.0271)	0.5762 (0.0130)	0.2608 (0.0579)	0.0001 (0.0001)	0.9075 (0.0330)
LTN	0.9911 (0.0199)	1.0031 (0.0285)	0.9948 (0.0204)	0.3566 (0.0129)	0.9777 (0.0406)	0.0000 (0.0000)	0.6451 (0.0483)
RTN	0.9594 (0.0264)	0.9877 (0.0368)	0.9602 (0.0268)	0.3414 (0.0097)	0.9241 (0.0423)	0.0000 (0.0000)	0.7017 (0.0340)
TTN	0.9781 (0.0214)	0.9732 (0.0239)	0.9792 (0.0221)	0.5136 (0.0110)	0.9380 (0.0450)	0.0001 (0.0001)	0.7988 (0.0356)
TPD	0.9682 (0.0236)	0.9964 (0.0233)	0.9892 (0.0219)	0.5765 (0.0111)	0.2719 (0.0740)	0.9764 (0.0211)	0.9104 (0.0377)
Mean	0.9924	1.0072	0.9976	0.4854	0.5875	0.1401	0.8135

[1] BF: backfat thickness; LMD: loin muscle depth; ELMP: estimated lean meat percentage; LTN: left teat number; RTN: right teat number; TTN: total teat number; TPD: time spent eating per day

Supplementary Table 11. Prediction performance of different models in the Yorkshire pig dataset. The IFAM represents running the IFAM model using trait-specific significant SNPs generated from GWAS and functional annotations derived from the IFmut database. The Multi2R represents a genomic prediction model consisting of two random effects, one is composed of trait-specific significant SNPs and the other is composed of remaining SNPs. The MultiBLUP represents a genomic prediction model that contained 11 random effects, each of which is constructed using 10 functional annotations from the IFAM database and trait-specific significant SNPs. The BayesR represents running the BayesR model with the parameters, where the MCMC chain length was set to 10,000. The IFAM_PA represents running the IFAM model using pseudo annotation information, which is selected randomly from the genome based on the scale of true annotations from the IFmut database. The accuracy of genomic prediction was assessed by the correlation between estimated genetic values and phenotypic values using 20 repetitions. Statistical significance between IFAM and competing models was assessed via the corrected resampled two-sided t-test. The mean correlation values and standard errors of each trait are shown.

Trait[1]	IFAM	GBLUP	Multi2R	MultiBLUP	BayesR	IFAM_PA
D100	0.4249 (0.0033)	0.4117 (0.0034)**	0.4250 (0.0033)	0.4051 (0.0031)**	0.4269 (0.0034)	0.4121 (0.0031)**
BF100	0.6232 (0.0018)	0.6115 (0.0017)**	0.6230 (0.0017)	0.6150 (0.0019)**	0.6035 (0.0023)**	0.6115 (0.0017)**
LMA100	0.4736 (0.0030)	0.4506 (0.0028)**	0.4736 (0.0030)	0.4526 (0.0029)**	0.4804 (0.0031)*	0.4505 (0.0029)**
LTN	0.3826 (0.0029)	0.3655 (0.0031)**	0.3824 (0.0029)	0.3506 (0.0033)**	0.0785 (0.0052)**	0.3659 (0.0031)**
RTN	0.4040 (0.0044)	0.3766 (0.0047)**	0.4033 (0.0044)	0.3597 (0.0045)**	0.0864 (0.0059)**	0.3676 (0.0058)**
TTN	0.4966 (0.0037)	0.4788 (0.0040)**	0.4954 (0.0038)	0.4738 (0.0035)**	0.2122 (0.0069)**	0.4755 (0.0041)**
Mean	0.4675	0.4491	0.4671	0.4428	0.3146	0.4472

[1] D100: age adjusted 100 kg; BF100: backfat thickness adjusted to 100 kg; LMA100: loin muscle area adjusted to 100 kg; LTN: left teat number; RTN: right teat number; TTN: total teat number;

* represents a significant difference ($P < 0.05$) in the prediction accuracy between the competing model and IFAM. When the competing model is BayesR, LMA100: t(19)=-2.6904, P=0.0145, 95% CI [-0.0152, 0.0016];

** represents an extremely significant difference ($P < 0.01$) in the prediction accuracy between the competing model and IFAM. When the competing model is GBLUP, D100: t(19)=8.8893, P<1E-04, 95% CI [-0.0191, 0.0456], BF100: t(19)=8.4474, P<1E-04, 95% CI [-0.0066, 0.0301], LMA100:

t(19)=8.1845, P<1E-04, 95% CI [-0.0088, 0.0547], LTN: t(19)=7.4382, P<1E-04, 95% CI [-0.0094, 0.0435], RTN: t(19)=6.6741, P<1E-04, 95% CI [-0.0089, 0.0638], TTN: t(19)=6.4301, P<1E-04, 95% CI [-0.0099, 0.0455]; When the competing model is MultiBLUP, D100: t(19)=6.1180, P<1E-04, 95% CI [-0.0038, 0.0434], BF100: t(19)=4.5501, P=0.0002, 95% CI [-0.0010, 0.0174], LMA100: t(19)=5.7867, P<1E-04, 95% CI [-0.0008, 0.0429], LTN: t(19)=6.8631, P<1E-04, 95% CI [-0.0005, 0.0645], RTN: t(19)=8.2980, P<1E-04, 95% CI [-0.0110, 0.0997], TTN: t(19)=3.7715, P=0.0013, 95% CI [0.0062, 0.0394]; When the competing model is BayesR, BF100: t(19)=5.0645, P<1E-04, 95% CI [0.0045, 0.0349], LTN: t(19)=22.8625, P<1E-04, 95% CI [-0.4904, 1.0986], RTN: t(19)=23.0829, P<1E-04, 95% CI [-0.5230, 1.1583], TTN: t(19)=17.8018, P<1E-04, 95% CI [-0.2971, 0.8660]; When the competing model is IFAM_PA, D100: t(19)=5.2198, P<1E-04, 95% CI [-0.0053, 0.0311], BF100: t(19)=8.3753, P<1E-04, 95% CI [-0.0065, 0.0299], LMA100: t(19)=8.1303, P<1E-04, 95% CI [-0.0086, 0.0549], LTN: t(19)=7.0144, P<1E-04, 95% CI [-0.0080, 0.0414], RTN: t(19)=5.7289, P<1E-04, 95% CI [-0.0011, 0.0739], TTN: t(19)=5.6279, P<1E-04, 95% CI [-0.0045, 0.0467]

Supplementary Table 12. Prediction bias of different models in the Yorkshire pig dataset. The explanation of results is the same as that in the Supplementary Table 11.

Trait[1]	IFAM	GBLUP	Multi2R	MultiBLUP	BayesR	IFAM_PA
D100	0.9928 (0.0092)	0.9998 (0.0092)	0.9963 (0.0087)	0.6896 (0.0057)	6.6558 (2.0986)	0.9196 (0.0168)
BF100	0.9988 (0.0047)	1.0023 (0.0051)	0.9990 (0.0047)	0.8688 (0.0058)	0.1112 (0.3692)	1.0020 (0.0052)
LMA100	0.9971 (0.0076)	1.0061 (0.0080)	0.9973 (0.0076)	0.7078 (0.0071)	0.8790 (0.7024)	0.9933 (0.0131)
LTN	0.9596 (0.0130)	0.9506 (0.0143)	0.9595 (0.0131)	0.5964 (0.0092)	0.2880 (0.1831)	0.8854 (0.0151)
RTN	0.0102 (0.0158)	0.9993 (0.0173)	1.0100 (0.0157)	0.6121 (0.0100)	0.0133 (0.1495)	0.8048 (0.0342)
TTN	0.9790 (0.0109)	0.9778 (0.0120)	0.9776 (0.0111)	0.7461 (0.0096)	0.4093 (0.3545)	0.9005 (0.0195)
Mean	0.9896	0.9893	0.9899	0.7036	0.3702	0.9176

[1] D100: age adjusted 100 kg; BF100: backfat thickness adjusted to 100 kg; LMA100: loin muscle area adjusted to 100 kg; LTN: left teat number; RTN: right teat number; TTN: total teat number

Supplementary Table 13. The computational efficiency of different models based on the Yorkshire pig dataset. All results were computed on a Linux server with 32 CPUs and 960 GB of memory and the computational times (h) were recorded. For the Adaptive MultiBLUP, the procedure was killed during the variance component estimation for D100, BF100, and LMA100 traits.

Trait[1]	IFAM	GBLUP	Adaptive MultiBLUP	BayesR
D100	0.90	0.35	1.67	>24
BF100	0.90	0.36	1.79	>24
LMA100	0.77	0.31	1.68	>24
LTN	0.61	0.25	2.16	>24
RTN	0.65	0.27	1.60	>24
TTN	0.58	0.24	1.60	>24

[1] D100: age adjusted 100 kg; BF100: backfat thickness adjusted to 100 kg; LMA100: loin muscle area adjusted to 100 kg; LTN: left teat number; RTN: right teat number; TTN: total teat number

Supplementary Table 14. Prediction performance of different models in the rice dataset. The IFAM represents running the IFAM model using trait-specific significant SNPs generated from GWAS and functional annotations derived from the RGAP database. The Multi2R represents a genomic prediction model consisting of two random effects, one is composed of trait-specific significant SNPs and the other is composed of remaining SNPs. The MultiBLUP represents a genomic prediction model that contained 9 random effects, each of which is constructed using 8 functional annotations from the RGAP database and trait-specific significant SNPs. The BayesR represents running the BayesR model with default parameters, where the MCMC chain length was set to 50,000. The IFAM_PA represents running the IFAM model using pseudo annotation information, which is selected randomly from the genome based on the scale of true annotations from the RGAP database. The accuracy of genomic prediction was assessed by the correlation between estimated genetic values and phenotypic values using 20 repetitions. Statistical significance between IFAM and competing models was assessed via the corrected resampled two-sided t-test. The mean correlation values and standard errors of each trait are shown.

Trait[1]	IFAM	GBLUP	Multi2R	MultiBLUP	Adaptive MultiBLUP	BayesR	BayesRC	IFAM_PA
GL	0.4874<br>(0.0088)	0.4165<br>(0.0113)[**]	0.4876<br>(0.0087)	0.4573<br>(0.0091)	0.4620<br>(0.0103)[*]	0.5169<br>(0.0107)[*]	0.0358<br>(0.0105)[**]	0.4134<br>(0.0119)[**]
GW	0.5682<br>(0.0075)	0.5168<br>(0.0087)[**]	0.5687<br>(0.0077)	0.5459<br>(0.0078)[*]	0.5301<br>(0.0082)[**]	0.5588<br>(0.0086)	-0.1972<br>(0.1005)[**]	0.5150<br>(0.0088)[**]
GLWR	0.6264<br>(0.0085)	0.5301<br>(0.0098)[**]	0.6269<br>(0.00085)	0.5903<br>(0.0097)[**]	0.5707<br>(0.0094)[**]	0.6388<br>(0.0100)	0.0332<br>(0.0142)[**]	0.5270<br>(0.0098)[**]
DH	0.4643<br>(0.0049)	0.4467<br>(0.0070)	0.4661<br>(0.0050)	0.4301<br>(0.0049)[**]	0.4636<br>(0.0077)	0.4653<br>(0.0066)	0.2216<br>(0.0068)[**]	0.4411<br>(0.0070)[*]
TGW	0.4972<br>(0.0105)	0.4750<br>(0.0095)	0.4972<br>(0.0106)	0.4968<br>(0.0097)	0.4810<br>(0.0108)	0.5051<br>(0.0100)	0.1396<br>(0.0102)[**]	0.4738<br>(0.0095)[*]
Mean	0.5287	0.4770	0.5293	0.5041	0.5029	0.5370	0.0466	0.4741

[1] GL: grain length; GW: grain width; GLWR: grain length-to-width ratio; DH: days to heading; TGW: thousand grain weight;

[*] represents a significant difference ($P < 0.05$) in the prediction accuracy between the competing model and IFAM. When the competing model is MultiBLUP, GW: t(19)=2.5533, P=0.0194, 95% CI [0.0019, 0.0426]; When the competing model is Adaptive MultiBLUP, GL: t(19)=2.1092, P=0.0484, 95% CI [0.0046, 0.0462]; When the competing model is BayesR, GL: t(19)=-2.2158,

P=0.0391, 95% CI [-0.0521, -0.0069]; When the competing model is IFAM_PA, DH: t(19)=2.4506, P=0.0241, 95% CI [0.0072, 0.0393], TGW: t(19)=2.2268, P=0.0383, 95% CI [0.0005, 0.0462];
** represents an extremely significant difference ($P < 0.01$) in the prediction accuracy between the competing model and IFAM. When the competing model is GBLUP, GL: t(19)=3.7420, P=0.0014, 95% CI [0.0229, 0.1190], GW: t(19)=4.2933, P=0.0004, 95% CI [0.0088, 0.0939], GLWR: t(19)=8.1094, P<1E-04, 95% CI [-0.0187, 0.2111]; When the competing model is MultiBLUP, GLWR: t(19)=4.1259, P=0.0006, 95% CI [-0.0047, 0.0769], DH: t(19)=5.7457, P<1E-04, 95% CI [-0.0014, 0.0700]; When the competing model is Adaptive MultiBLUP, GW: t(19)=3.4418, P=0.0027, 95% CI [0.0075, 0.0687], GLWR: t(19)=4.1280, P=0.0006, 95% CI [0.0108, 0.1005]; When the competing model is BayesRC, GL: t(19)=13.7956, P<1E-04, 95% CI [-0.2661, 1.1694], GW: t(19)=21.5814, P<1E-04, 95% CI [-0.8624, 2.0399], GLWR: t(19)=19.5827, P<1E-04, 95% CI [-0.7415, 1.9277], DH: t(19)=12.8945, P<1E-04, 95% CI [-0.1196, 0.6050], TGW: t(19)=11.0325, P<1E-04, 95% CI [-0.1032, 0.8182]; When the competing model is IFAM_PA, GL: t(19)=3.8311, P=0.0011, 95% CI [0.0231, 0.1250], GW: t(19)=4.2754, P=0.0004, 95% CI [0.0100, 0.0963], GLWR: t(19)=8.2480, P<1E-04, 95% CI [-0.0197, 0.2185]

Supplementary Table 15. Prediction bias of the different models in the rice dataset. The explanation of results is the same as that in the Supplementary Table 14.

Trait[1]	IFAM	GBLUP	Multi2R	MultiBLUP	Adaptive MultiBLUP	BayesR	IFAM_PA
GL	0.9629 (0.0291)	0.9930 (0.0426)	0.9658 (0.0283)	0.7507 (0.0210)	0.9781 (0.0323)	0.9476 (0.0285)	0.9731 (0.0438)
GW	0.9990 (0.0246)	1.0582 (0.0309)	1.0091 (0.0269)	0.8264 (0.0182)	1.0336 (0.0292)	0.9617 (0.0258)	1.0489 (0.0318)
GLWR	0.9659 (0.0216)	0.9586 (0.0267)	0.9683 (0.0217)	0.8317 (0.0190)	0.9667 (0.0273)	0.9544 (0.0216)	0.9526 (0.0275)
DH	0.8998 (0.0175)	0.9471 (0.0225)	0.9333 (0.0188)	0.6815 (0.0127)	0.9181 (0.0216)	0.8636 (0.0204)	0.8964 (0.0267)
TGW	0.9592 (0.0250)	0.9814 (0.0264)	0.9599 (0.0250)	0.8171 (0.0174)	0.9415 (0.0241)	0.9215 (0.0241)	0.9995 (0.0278)
Mean	0.9574	0.9877	0.9673	0.7815	0.9694	0.9298	0.9741

[1] GL: grain length; GW: grain width; GLWR: grain length-to-width ratio; DH: days to heading; TGW: thousand grain weight

Supplementary Table 16. Prediction performance of different scenarios of Adaptive MultiBLUP in the Duroc pig dataset. The Adaptive MultiBLUP represents running the Adaptive MultiBLUP using the default parameters set, where the chunks of size were set to 75,000 base pairs; The Adaptive MultiBLUP opt1 represents running the Adaptive MultiBLUP with the chunks size of 50,000 base pairs; The Adaptive MultiBLUP opt2 represents running the Adaptive MultiBLUP with the chunks size of 500,000 base pairs; The Adaptive MultiBLUP opt3 represents running the Adaptive MultiBLUP with the chunks size of 1,000,000 base pairs. Statistical significance between IFAM and competing models was assessed via the corrected resampled two-sided t-test.

Trait[1]	Adaptive MultiBLUP 75K	Adaptive MultiBLUP opt1 50K	Adaptive MultiBLUP opt2 500K	Adaptive MultiBLUP opt3 1000 K
BF	0.1987(0.0337)	0.1972(0.0307)	0.1468(0.0336)	0.1521(0.0261)
LMD	0.2201(0.0266)	0.2184(0.0295)	0.1688(0.0271)	0.1384(0.0216) [3]
LMP	0.1955(0.0256)	0.1893(0.0379)	0.1145(0.0252)	0.0513(0.0235) [3]
LTN	0.3333(0.0105)	0.3397(0.0060)	0.2807(0.0247)	0.2154(0.0259)
RTN	0.2724(0.0086)	0.2795(0.0077)	0.1502(0.0251)*	0.2232(0.0165)
TTN	0.3973(0.0106)	0.3970(0.0115)	0.2675(0.0306)	0.1611(0.0313)[3,**]
TPD	0.2006(0.0311)	0.2409(0.0335)	0.0699(0.0246)	/ [2]
Mean	0.2597	0.2660	0.1712	0.1637

[1] BF: backfat thickness; LMD: loin muscle depth; ELMP: estimated lean meat percentage; LTN: left teat number; RTN: right teat number; TTN: total teat number; TPD: time spent eating per day;

[2] represents a missing value caused by the abnormal estimation of variance components;

[3] represents some repetitions that did not yield results caused by the abnormal estimation of variance components;

* represents a significant difference ($P < 0.05$) in the prediction accuracy between the competing scenario and Adaptive MultiBLUP. When the competing model is Adaptive MultiBLUP opt2 500 K, RTN: t(19)=2.2797, P=0.0344, 95% CI [0.0695, 0.1749];

** represents an extremely significant difference ($P < 0.01$) in the prediction accuracy between the competing scenario and Adaptive MultiBLUP. When the competing model is Adaptive MultiBLUP opt3 1000 K, TTN: t(19)=3.1311, P=0.0055, 95% CI [0.1206, 0.3568]

Supplementary Table 17. The estimates of genetic variance component were computed by GBLUP, IFAM, and a genomic prediction model that contains multiple random effects (MultiBLUP) using the WTCCC1 dataset. The MultiBLUP model contained 8 random effects, each of which was constructed using 7 functional annotations from the RegulomeDB database and significant SNPs of trait identified through GWAS. The standard errors of the estimates are shown in parentheses.

**Trait**[1]	**BD**	**CAD**	**CD**	**HT**	**RA**	**T1D**	**T2D**
GBLUP	0.1720 (0.0016)	0.1162 (0.0018)	0.1521 (0.0016)	0.1181 (0.0022)	0.1405 (0.0017)	0.1721 (0.0019)	0.1200 (0.0016)
IFAM[2]	0.1637 (0.0053)	0.1168 (0.0017)	0.1419 (0.0015)	0.1187 (0.0022)	0.0993 (0.0016)	0.2069 (0.0022)	0.1204 (0.0015)
MultiBLUP[3]	0.1689 (0.0017)	0.1253 (0.0024)	0.1636 (0.0016)	0.1213 (0.0019)	0.1326 (0.0045)	0.1392 (0.0020)	0.1436 (0.0020)
MultiBLUP-1	0.0069 (0.0010)	0.0076 (0.0009)	0.0197 (0.0006)	0.0105 (0.0011)	0.0098 (0.0011)	0.0100 (0.0005)	0.0118 (0.0007)
MultiBLUP-2	0.0426 (0.0029)	0.0398 (0.0033)	0.0540 (0.0023)	0.0303 (0.0037)	0.0423 (0.0018)	0.0210 (0.0015)	0.0299 (0.0021)
MultiBLUP-3	0.0054 (0.0011)	0.0013 (0.0006)	0.0160 (0.0008)	0.0020 (0.0006)	0.0110 (0.0010)	0.0053 (0.0009)	0.0114 (0.0008)
MultiBLUP-4	0.0296 (0.0037)	0.0240 (0.0030)	0.0001 (0.0001)	0.0291 (0.0036)	0.0140 (0.0022)	0.0019 (0.0008)	0.0033 (0.0012)
MultiBLUP-5	0.0010 (0.0002)	0.0033 (0.0005)	0.0044 (0.0005)	0.0028 (0.0004)	0.0026 (0.0008)	0.0018 (0.0005)	0.0018 (0.0004)
MultiBLUP-6	0.0145 (0.0016)	0.0088 (0.0014)	0.0095 (0.0011)	0.0063 (0.0013)	0.0141 (0.0014)	0.0131 (0.0012)	0.0201 (0.0012)
MultiBLUP-7	0.0685 (0.0046)	0.0388 (0.0041)	0.0413 (0.0028)	0.0397 (0.0046)	0.0076 (0.0022)	0.0210 (0.0022)	0.0622 (0.0018)
MultiBLUP-8	0.0011 (0.0001)	0.0016 (0.0002)	0.0186 (0.0007)	0.0006 (0.0001)	0.0312 (0.0005)	0.0650 (0.0035)	0.0031 (0.0006)

[1] BD: bipolar disorder; CAD: coronary artery disease; CD: Crohn's disease; HT: hypertension; RA: rheumatoid arthritis; T1D: type 1 diabetes; T2D: type 2 diabetes;

[2] IFAM: the sum of genetic variance component estimates of optimized random effects in the IFAM model;

[3] MultiBLUP: the sum of genetic variance component estimates of many random effects in the MultiBLUP model

Supplementary Table 18. The estimates of genetic variance component were computed by GBLUP, IFAM, and a genomic prediction model that contains multiple random effects (MultiBLUP) using the Duroc pig dataset. The MultiBLUP model contained 11 random effects, each of which was constructed using 10 functional annotations from the IFmut database and significant SNPs of trait identified through GWAS. The "/" represents an empty value because there are no significant SNPs for this trait. The standard errors of the estimates are shown in parentheses.

Trait[1]	BF	LMD	LMP	LTN	RTN	TTN	TPD
GBLUP	1.1403(0.0138)	4.4596(0.0599)	0.8092(0.0096)	0.0881(0.0013)	0.0706(0.0012)	0.3449(0.0037)	35.9214(0.3766)
IFAM[2]	1.4888(0.0323)	4.4497(0.0588)	0.8750(0.0139)	0.0871(0.0014)	0.0701(0.0012)	0.3337(0.0039)	49.1368(3.1658)
MultiMR[3]	4.0533(0.0261)	13.9162(0.0629)	2.3241(0.0107)	0.4294(0.0028)	0.4203(0.0038)	1.1228(0.0103)	94.1294(0.4546)
MultiMR-1	0.2990(0.0352)	1.9693(0.1571)	0.4201(0.0200)	0.0321(0.0027)	0.0633(0.0032)	0.1937(0.0069)	19.6595(1.0803)
MultiMR-2	0.0763(0.0282)	1.1669(0.0936)	0.3017(0.0174)	0.0104(0.0023)	0.0051(0.0018)	0.0324(0.0075)	6.1616(0.4902)
MultiMR-3	0.3492(0.0249)	2.1687(0.0794)	0.1646(0.0114)	0.0335(0.0018)	0.0356(0.0023)	0.0543(0.0049)	4.5692(0.4419)
MultiMR-4	0.2598(0.0103)	1.0069(0.0498)	0.1954(0.0037)	0.0404(0.0009)	0.0410(0.0011)	0.1069(0.0025)	7.6109(0.2263)
MultiMR-5	0.3095(0.0136)	0.5845(0.0742)	0.1470(0.0071)	0.0289(0.0010)	0.0186(0.0013)	0.0477(0.0042)	7.6772(0.2996)
MultiMR-6	0.2897(0.0327)	1.9002(0.0959)	0.0261(0.0111)	0.0067(0.0020)	0.0624(0.0044)	0.0199(0.0057)	5.2134(0.7744)
MultiMR-7	0.0949(0.0250)	1.5931(0.1143)	0.1907(0.0187)	0.0464(0.0034)	0.0204(0.0037)	0.1114(0.0095)	4.2053(0.6225)
MultiMR-8	0.3809(0.0420)	0.2335(0.0745)	0.1143(0.0221)	0.0486(0.0049)	0.0203(0.0036)	0.0949(0.0126)	0.6743(0.4642)
MultiMR-9	0.5246(0.0270)	0.7135(0.0965)	0.2086(0.0163)	0.0421(0.0034)	0.0397(0.0035)	0.0895(0.0077)	12.0317(0.8617)
MultiMR-10	0.9780(0.0472)	2.5797(0.1292)	0.3543(0.0143)	0.1123(0.0068)	0.0804(0.0036)	0.2769(0.0133)	15.9546(0.6293)
MultiMR-11	0.4913(0.0109)	/	0.2014(0.0035)	0.0280(0.0021)	0.0334(0.0015)	0.0954(0.0031)	10.3716(0.2943)

[1] BF: backfat thickness; LMD: loin muscle depth; ELMP: estimated lean meat percentage; LTN: left teat number; RTN: right teat number; TTN: total teat number; TPD: time spent eating per day;

[2] IFAM: the sum of genetic variance component estimates of optimized random effect in the IFAM model;

[3] MultiBLUP: the sum of genetic variance component estimates of many random effects in the MultiBLUP model

Supplementary Table 19. The estimates of genetic variance component were computed by GBLUP, IFAM, and a genomic prediction model that contains multiple random effects (MultiBLUP) using the Yorkshire pig dataset. The MultiBLUP model contained 11 random effects, each of which was constructed using 10 functional annotations from the IFmut database and significant SNPs of trait identified through GWAS. The standard errors of the estimates are shown in parentheses.

Trait[1]	D100	BF100	LMA100	LTN	RTN	TTN
GBLUP	27.0381(0.1220)	2.5222(0.0089)	5.9849(0.0270)	0.1051(0.0008)	0.1086(0.0008)	0.4419(0.0021)
IFAM[2]	38.1065(0.6502)	3.0511(0.0234)	9.2193(0.1997)	0.0961(0.0007)	0.3896(0.0023)	0.0994(0.0006)
MultiMR[3]	89.5741(0.7264)	4.3580(0.0292)	16.8152(0.0956)	0.3689(0.0029)	0.3648(0.0053)	0.9215(0.0183)
MultiMR-1	5.6016(0.6434)	0.7812(0.0242)	2.2266(0.0755)	0.0375(0.0026)	0.0731(0.0030)	0.1554(0.0063)
MultiMR-2	8.0726(0.3742)	0.2407(0.0224)	1.5191(0.0414)	0.0330(0.0016)	0.0156(0.0020)	0.0605(0.0064)
MultiMR-3	9.0526(0.2346)	0.3693(0.0146)	1.7863(0.0234)	0.0353(0.0011)	0.0338(0.0014)	0.0812(0.0033)
MultiMR-4	4.2896(0.1920)	0.2453(0.0068)	0.9468(0.0294)	0.0184(0.0010)	0.0235(0.0009)	0.0501(0.0035)
MultiMR-5	5.4360(0.1882)	0.2118(0.0095)	1.1170(0.0264)	0.0327(0.0005)	0.0218(0.0014)	0.0682(0.0032)
MultiMR-6	10.9129(0.5066)	0.0200(0.0101)	1.7351(0.0577)	0.0009(0.0007)	0.0150(0.0030)	0.0065(0.0037)
MultiMR-7	3.9616(0.5562)	0.2149(0.0217)	0.5117(0.0772)	0.0153(0.0022)	0.0497(0.0017)	0.1018(0.0041)
MultiMR-8	0.8907(0.4384)	0.1946(0.0353)	0.0174(0.0123)	0.0496(0.0029)	0.0066(0.0030)	0.0787(0.0103)
MultiMR-9	6.6884(0.3750)	0.3484(0.0229)	1.9704(0.0619)	0.0434(0.0018)	0.0218(0.0021)	0.0497(0.0068)
MultiMR-10	21.5669(0.9198)	1.0409(0.0331)	2.6792(0.0417)	0.0687(0.0024)	0.0769(0.0021)	0.1861(0.0071)
MultiMR-11	13.1012(0.3105)	0.6910(0.0135)	2.3056(0.0456)	0.0342(0.0009)	0.0270(0.0012)	0.0832(0.0022)

[1] D100: age adjusted 100 kg; BF100: backfat thickness adjusted to 100 kg; LMA100: loin muscle area adjusted to 100 kg; LTN: left teat number; RTN: right teat number; TTN: total teat number;

[2] IFAM: the sum of genetic variance component estimates of optimized random effect in the IFAM model;

[3] MultiBLUP: the sum of genetic variance component estimates of many random effects in the MultiBLUP model

Supplementary Table 20. The estimates of genetic variance component were computed by GBLUP, IFAM, and a genomic prediction model that contains multiple random effects (MultiBLUP) using the rice dataset. The MultiBLUP model contained 9 random effects, each of which was constructed using 8 functional annotations from the RGAP database and significant SNPs of trait identified through GWAS. The standard errors of the estimates are shown in parentheses.

**Trait**[1]	**GL**	**GW**	**GLWR**	**DH**	**TGW**
GBLUP	0.5968(0.0103)	0.0842(0.0011)	0.1798(0.0023)	86.8594(1.1003)	11.0367(0.1482)
IFAM[2]	0.5657(0.0126)	0.0812(0.0015)	0.1522(0.0022)	89.9832(1.2288)	10.9121(0.1625)
MultiMR[3]	0.6884(0.0030)	0.1111(0.0007)	0.1838(0.0008)	103.1152(0.3440)	11.5204(0.1214)
MultiMR-1	0.0673(0.0015)	0.0122(0.0002)	0.0196(0.0002)	10.8378(0.0903)	1.1325(0.0212)
MultiMR-2	0.0588(0.0042)	0.0157(0.0015)	0.0258(0.0016)	20.8150(1.1247)	1.1804(0.1321)
MultiMR-3	0.0105(0.0037)	0.0162(0.0022)	0.0067(0.0017)	14.3460(0.9085)	0.5671(0.1622)
MultiMR-4	0.1084(0.0082)	0.0105(0.0016)	0.0322(0.0006)	14.2484(0.8168)	1.8285(0.1418)
MultiMR-5	0.0342(0.0102)	0.0125(0.0021)	0.0022(0.0009)	7.1763(1.0250)	0.4956(0.1355)
MultiMR-6	0.0744(0.0016)	0.0115(0.0003)	0.0211(0.0001)	11.3117(0.1070)	1.3051(0.0176)
MultiMR-7	0.1711(0.0070)	0.0070(0.0025)	0.0322(0.0027)	0.6267(0.3875)	2.3688(0.2187)
MultiMR-8	0.0815(0.0015)	0.0119(0.0004)	0.0225(0.0002)	12.1572(0.1862)	1.3244(0.0252)
MultiMR-9	0.0821(0.0016)	0.0137(0.0003)	0.0216(0.0002)	11.5962(0.0581)	1.3180(0.0179)

[1] GL: grain length; GW: grain width; GLWR: grain length-to-width ratio; DH: days to heading; TGW: thousand grain weight;

[2] IFAM: the sum of genetic variance component estimates of optimized random effect in the IFAM model;

[3] MultiBLUP: the sum of genetic variance component estimates of many random effects in the MultiBLUP model

Supplementary Table 21. The data information for the WTCCC1 dataset. This dataset had 7 disease traits, and quality control was carried out using PLINK software (v1.90).

Trait	Ori data	Data after quality control
Bipolar disorder (BD)	458,868 SNPs, 4,806 samples, 1,868 cases	373,369 SNPs, 4,806 samples, 1,868 cases
Coronary artery disease (CAD)	458,868 SNPs, 4,864 samples, 1,926 cases	372,541 SNPs, 4,864 samples, 1,926 cases
Crohn's disease (CD)	458,868 SNPs, 4,686 samples, 1,748 cases	374,113 SNPs, 4,686 samples, 1,748 cases
Hypertension (HT)	458,868 SNPs, 4,890 samples, 1,952 cases	373,338 SNPs, 4,890 samples, 1,952 cases
Rheumatoid arthritis (RA)	458,868 SNPs, 4,798 samples, 1,860 cases	373,056 SNPs, 4,798 samples, 1,860 cases
Type 1 diabetes (T1D)	458,868 SNPs, 4,901 samples, 1,963 cases	372,964 SNPs, 4,901 samples, 1,963 cases
Type 2 diabetes (T2D)	458,868 SNPs, 4,862 samples, 1,924 cases	373,149 SNPs, 4,862 samples, 1,924 cases

Supplementary Table 22. The functional annotations for human from the RegulomeDB database, which have 7 types[10].

Number	Type
1	eQTL
2	TF binding
3	matched TF motif
4	motif
5	matched DNase footprint
6	DNase footprint
7	DNase peak

Supplementary Table 23. The RegulomeDB variant classification scheme. There are 7 ranks. The genomic coverage of this annotation was detailed in Supplementary Fig. 12.

Category	SNPs	Description
Rank 1	75,189	Linked to expression of gene parget and likely to affect binding<br>eQTL + TF binding + matched TF motif + any motif + matched DNase footprint + DNase footprint + DNase peak
Rank 2	449,374	Likely to affect binding<br>TF binding + matched TF motif + any motif + matched DNase footprint + DNase footprint + DNase peak
Rank 3	847,825	Less likely to affect binding<br>TF binding + any motif + matched TF motif + DNase peak
Rank 4	1,831,986	Minimal binding evidence<br>TF binding + DNase peak
Rank 5	4,880,805	TF binding / DNase peak
Rank 6	2,469,270	Motif hit
Rank 7	2,720,574	Other SNPs on the genome

Supplementary Table 24. The genomic functional annotations for human from the website of the LD Scores Regression (LDSC) model include 74 types, and see the study about the stratified LDSC model for more details[11].

Numbe	Type	Number	Type
1	Ancient_Sequence_Age_Human_Enhancer	38	H3K4me3_Trynka.extend.500
2	Ancient_Sequence_Age_Human_Enhancer.extend.500	39	H3K9ac_peaks_Trynka
3	Ancient_Sequence_Age_Human_Promoter	40	H3K9ac_Trynka
4	Ancient_Sequence_Age_Human_Promoter.extend.500	41	H3K9ac_Trynka.extend.500
5	BivFlnk	42	Human_Enhancer_Villar
6	BivFlnk.extend.500	43	Human_Enhancer_Villar.extend.500
7	Coding_UCSC	44	Human_Promoter_Villar_ExAC
8	Coding_UCSC.extend.500	45	Human_Promoter_Villar_ExAC.extend.500
9	Conserved_LindbladToh	46	Human_Promoter_Villar
10	Conserved_LindbladToh.extend.500	47	Human_Promoter_Villar.extend.500
11	Conserved_Mammal_phastCons46way	48	Intron_UCSC
12	Conserved_Mammal_phastCons46way.extend.500	49	Intron_UCSC.extend.500
13	Conserved_Primate_phastCons46way	50	Promoter_UCSC
14	Conserved_Primate_phastCons46way.extend.500	51	Promoter_UCSC.extend.500
15	Conserved_Vertebrate_phastCons46way	52	PromoterFlanking_Hoffman
16	Conserved_Vertebrate_phastCons46way.extend.500	53	PromoterFlanking_Hoffman.extend.500
17	CTCF_Hoffman	54	Repressed_Hoffman
18	CTCF_Hoffman.extend.500	55	Repressed_Hoffman.extend.500
19	DGF_ENCODE	56	Repressed_Hoffman.extend.500
20	DGF_ENCODE.extend.500	57	SuperEnhancer_Hnisz
21	DHS_Trynka	58	SuperEnhancer_Hnisz.extend.500
22	DHS_Trynka.extend.500	59	TFBS_ENCODE
23	Enhancer_Andersson	60	TFBS_ENCODE.extend.500
24	Enhancer_Andersson.extend.500	61	Transcribed_Hoffman
25	Enhancer_Hoffman	62	Transcribed_Hoffman.extend.500
26	Enhancer_Hoffman.extend.500	63	TSS_Hoffman
27	FetalDHS_Trynka	64	TSS_Hoffman.extend.500
28	FetalDHS_Trynka.extend.500	65	UTR_3_UCSC
29	H3K27ac_Hnisz	66	UTR_3_UCSC.extend.500
30	H3K27ac_Hnisz.extend.500	67	UTR_5_UCSC
31	H3K27ac_PGC2	68	UTR_5_UCSC.extend.500
32	H3K27ac_PGC2.extend.500	69	Vahedi_Tcell_SE_500bp
33	H3K4me1_peaks_Trynka	70	Vahedi_Tcell_SE
34	H3K4me1_Trynka	71	Vahedi_Tcell_TE_500bp
35	H3K4me1_Trynka.extend.500	72	Vahedi_Tcell_TE
36	H3K4me3_peaks_Trynka	73	WeakEnhancer_Hoffman
37	H3K4me3_Trynka	74	WeakEnhancer_Hoffman.extend.500

Supplementary Table 25. The functional annotations for pig from the IFmut database. There were 10 types[12].

Number	Type
1	open chromatin region
2	nucleosome free region
3	footprint
4	matched_motif
5	intersect_motif
6	any_motif
7	active_promoter_narrowPeak
8	enhancer_narrowPeak
9	active_promoter
10	enhancer

Supplementary Table 26. The annotations for rice from the Rice Genome Annotation Project (RGAP) database. There were 8 types[13].

Number	Type[1]
1	TE
2	expressed
3	representative
4	CDS
5	exon
6	5’ UTR
7	gene
8	3’ UTR

[1] TE: the site is related to transposon or retro-transposon elements; expressed: the site has expression support; representative: the site is the representative; CDS: Coding sequence

Supplementary Table 27. Prediction performance of different scenarios of BayesR in the WTCCC1 dataset. The BayesR represents running the BayesR using the default parameters, where the MCMC chain length (Numit) was set to 50,000, and the burnin steps were set to 20,000. The BayesR_opt1 represents running the BayesR using the alternative parameters, where the MCMC chain length (Numit) was set to 10,000, and the burnin steps were set to 4,000. BayesR_opt2 represents running the BayesR using the alternative parameters, where the MCMC chain length (Numit) was set to 100,000, and the burnin steps were set to 40,000.

**Trait**	**BayesR Numit=50K**	**BayesR_opt1 Numit=10K**	**BayesR_opt2 Numit=100K**
BD	0.6528(0.0037)	0.6503(0.0037)	0.6540(0.0037)
CAD	0.5975(0.0027)	0.5952(0.0027)	0.5978(0.0029)
CD	0.6708(0.0024)	0.6705(0.0026)	0.6710(0.0025)
HT	0.5932(0.0034)	0.5927(0.0033)	0.5940(0.0036)
RA	0.7152(0.0034)	0.7137(0.0034)	0.7156(0.0034)
T1D	0.8618(0.0019)	0.8612(0.0020)	0.8620(0.0020)
T2D	0.6179(0.0031)	0.6164(0.0034)	0.6187(0.0026)
Mean	0.6727	0.6714	0.6733

[1] BD: bipolar disorder; CAD: coronary artery disease; CD: Crohn’s disease; HT: hypertension; RA: rheumatoid arthritis; T1D: type 1 diabetes; T2D: type 2 diabetes

Supplementary Table 28. Examples of the importance assessment of functional annotation information in the UK Biobank dataset. The functional annotation information was the ranking scores from the RegulomeDB database. The Haseman–Elston (HE) regression algorithm was chosen to estimate the variance component and heritability. The standard errors of the estimates are shown in parentheses.

Category	HT[1]	BMR	hBMD	FVC	BMI	FEV	Mean
Rank 1	4.90E-06 (0.0010)	1.78E-06 (0.0006)	2.15E-06 (0.0009)	1.53E-06 (0.0006)	1.55E-06 (0.0007)	1.58E-06 (0.0006)	2.25E-06
Rank 2	2.58E-06 (0.0016)	1.17E-06 (0.0013)	6.67E-07 (0.0017)	8.54E-07 (0.0013)	8.12E-07 (0.0010)	8.60E-07 (0.0012)	1.16E-06
Rank 3	1.77E-06 (0.0021)	6.65E-07 (0.0016)	1.37E-06 (0.0025)	5.42E-07 (0.0016)	3.69E-07 (0.0015)	5.06E-07 (0.0017)	8.70E-07
Rank 4	3.14E-06 (0.0026)	1.68E-06 (0.0025)	1.32E-06 (0.0030)	1.03E-06 (0.0023)	8.94E-07 (0.0024)	1.06E-06 (0.0023)	1.52E-06
Rank 5	7.38E-07 (0.0028)	5.83E-07 (0.0029)	9.18E-07 (0.0043)	4.34E-07 (0.0028)	7.45E-07 (0.0029)	5.21E-07 (0.0028)	6.56E-07
Rank 6	8.05E-08 (0.0019)	3.60E-07 (0.0020)	--[2]	1.15E-07 (0.0019)	5.65E-07 (0.0018)	1.20E-07 (0.0020)	1.53E-07
Rank 7	3.79E-08 (0.0021)	2.60E-07 (0.0024)	--	1.66E-07 (0.0020)	3.88E-07 (0.0020)	2.23E-07 (0.0019)	1.78E-07

[1] HT: height; BMR: basal metabolic rate; hBMD: heel bone mineral density T-score; FVC: forced vital capacity; FEV: forced expiratory volume in 1s; BMI: body mass index;

[2] "--": a missing value which is anomalous

Supplementary Table 29. Examples of the importance assessment of functional annotation information in the WTCCC1 dataset and the criterion of assessment is the heritability. The functional annotation information was the ranking scores from the RegulomeDB database. The average information restricted maximum likelihood (AI-REML) algorithm was chosen to estimate the heritability. The standard errors of the estimates are shown in parentheses.

Category	BD[1]	CAD	CD	HT	RA	T1D	T2D	Mean
Rank 1	1.36E-05 (0.0202)	1.21E-05 (0.0201)	2.09E-05 (0.0221)	1.27E-05 (0.0205)	2.33E-05 (0.0209)	3.81E-06 (0.0216)	1.22E-05 (0.0205)	1.90E-05
Rank 2	1.01E-05 (0.0274)	4.22E-06 (0.0269)	9.58E-06 (0.0282)	5.54E-06 (0.0274)	1.03E-05 (0.0279)	4.41E-06 (0.0258)	4.03E-06 (0.0277)	6.89E-06
Rank 3	1.80E-06 (0.0398)	6.66E-06 (0.0399)	3.89E-06 (0.0396)	2.20E-06 (0.0388)	4.55E-06 (0.0398)	4.46E-06 (0.0372)	3.54E-06 (0.0393)	3.87E-06
Rank 4	2.52E-06 (0.0558)	3.55E-06 (0.0559)	6.54E-06 (0.0576)	2.80E-06 (0.0551)	4.45E-06 (0.0542)	4.11E-06 (0.0537)	7.36E-06 (0.0568)	4.48E-06
Rank 5	5.66E-06 (0.0749)	2.92E-06 (0.0757)	2.80E-06 (0.0746)	1.47E-06 (0.0741)	--	9.05E-07 (0.0723)	1.59E-06 (0.0738)	2.19E-06
Rank 6	1.83E-06 (0.0487)	7.43E-07 (0.0475)	--	2.06E-06 (0.0490)	5.43E-06 (0.0481)	--	--	1.44E-06
Rank 7	1.92E-06 (0.0582)	--[2]	3.28E-07 (0.0583)	2.32E-06 (0.0578)	3.40E-06 (0.0596)	3.64E-06 (0.0573)	1.21E-06 (0.0572)	1.83E-06

[1] BD: bipolar disorder; CAD: coronary artery disease; CD: Crohn’s disease; HT: hypertension; RA: rheumatoid arthritis; T1D: type 1 diabetes; T2D: type 2 diabetes;

[2] “--”: a missing value which is anomalous

Supplementary Table 30. The importance assessment of functional annotations from the RegulomeDB database in the WTCCC1 dataset and the criterion of assessment is the heritability. The average information restricted maximum likelihood (AI-REML) algorithm was chosen to estimate the heritability. The standard errors of the estimates are shown in parentheses.

Type of annotation	BD[1]	CAD	CD	HT	RA	T1D	T2D	Mean
eQTL	2.44E-06 (0.0223)	2.63E-06 (0.0216)	1.44E-05 (0.0246)	4.94E-06 (0.0225)	2.23E-05 (0.0226)	2.92E-05 (0.0226)	8.78E-06 (0.0234)	1.21E-05
TF binding	3.59E-06 (0.1087)	3.71E-06 (0.1079)	4.67E-06 (0.1019)	1.98E-06 (0.1067)	3.64E-06 (0.0959)	2.54E-06 (0.0941)	2.59E-06 (0.1012)	3.25E-06
matched TF motif	4.82E-06 (0.0183)	2.78E-06 (0.0183)	3.36E-05 (0.0209)	5.25E-07 (0.0179)	3.35E-05 (0.0206)	3.32E-05 (0.0204)	2.37E-05 (0.0199)	1.89E-05
motif	2.37E-06 (0.0959)	1.20E-06 (0.0934)	--	2.31E-06 (0.0959)	2.67E-06 (0.0823)	--	1.36E-07 (0.0901)	1.24E-06
matched DNase footprint	1.55E-06 (0.0113)	9.93E-07 (0.0111)	1.42E-05 (0.0139)	6.34E-06 (0.0110)	5.50E-05 (0.0161)	4.57E-05 (0.0158)	--	1.77E-05
DNase footprint	4.35E-06 (0.0409)	4.82E-07 (0.0391)	5.00E-06 (0.0388)	1.59E-06 (0.0392)	6.35E-06 (0.0362)	6.89E-06 (0.0367)	7.26E-06 (0.0388)	4.56E-06
DNase peak	4.19E-06 (0.1362)	3.05E-06 (0.1357)	3.37E-06 (0.1272)	2.70E-06 (0.1364)	--	2.80E-06 (0.1188)	4.28E-06 (0.1282)	2.91E-06

[1] BD: bipolar disorder; CAD: coronary artery disease; CD: Crohn's disease; HT: hypertension; RA: rheumatoid arthritis; T1D: type 1 diabetes; T2D: type 2 diabetes;

[2] "--": a missing value which is anomalous

Supplementary Table 31. The number and proportion of SNPs with distinct functional annotations for 7 traits in the WTCCC1 dataset. The annotation process utilized 8 types of functional annotations from the RegulomeDB database along with significant SNPs identified through genome-wide association studies (GWAS) (Supplementary Table 36). The numbers outside the parentheses represent the number of annotated SNPs and the numbers inside the parentheses represent the proportion of annotated SNPs across the genome.

**Type of annotation**	**Bipolar disorder (BD)**	**Coronary artery disease (CAD)**	**Crohn's disease (CD)**	**Hypertension (HT)**	**Rheumatoid arthritis (RA)**	**Type 1 diabetes (T1D)**	**Type 2 diabetes (T2D)**
Significant SNPs	1 (0.00%)	50 (0.01%)	178 (0.05%)	23 (0.01%)	586 (0.16%)	825 (0.22%)	40 (0.01%)
eQTL	27,918 (7.48%)	27,914 (7.49%)	27,935 (7.47%)	27,872 (7.47%)	27,855 (7.47%)	27,652 (7.41%)	27,786 (7.45%)
TF binding	138,825 (37.18%)	138,306 (37.13%)	139,239 (37.22%)	138,878 (37.20%)	138,820 (37.21%)	138,706 (37.19%)	138,797 (37.20%)
matched TF motif	2,627 (0.70%)	2,631 (0.71%)	2,628 (0.70%)	2,647 (0.71%)	2,620 (0.70%)	2,635 (0.71%)	2,647 (0.71%)
motif	131,832 (35.31%)	131,544 (35.31%)	132,320 (35.37%)	131,745 (35.29%)	131,730 (35.31%)	131,690 (35.31%)	131,701 (35.29%)
matched DNase footprint	974 (0.26%)	981 (0.26%)	979 (0.26%)	987 (0.26%)	972 (0.26%)	974 (0.26%)	984 (0.26%)
DNase footprint	19,882 (5.33%)	19,776 (5.31%)	19,938 (5.33%)	19,869 (5.32%)	19,837 (5.32%)	19,846 (5.32%)	19,874 (5.33%)
DNase peak	225,259 (60.33%)	224,668 (60.31%)	225,832 (60.36%)	225,308 (60.35%)	225,179 (60.36%)	225,068 (60.35%)	225,205 (60.35%)

Supplementary Table 32. The average number and proportion of SNPs with distinct functional annotations in the UK Biobank (UKB) dataset. The annotation process utilized 8 types of functional annotations from the RegulomeDB database along with significant SNPs identified through genome-wide association studies (GWAS) (Supplementary Table 37). The numbers outside the parentheses represent the number of annotated SNPs, and the numbers inside the parentheses represent the proportion of annotated SNPs across the genome.

Type of annotation	UKB
Significant SNPs	6,013(0.55%)
eQTL	56,262(5.14%)
TF binding	439,789(40.17%)
matched TF motif	9,324(0.85%)
motif	415,621(37.96%)
matched DNase footprint	3,990(0.36%)
DNase footprint	71,470(6.53%)
DNase peak	692,277(63.23%)

Supplementary Table 33. The average number and proportion of SNPs with distinct functional annotations in the Duroc pig and Yorkshire pig datasets. The annotation process utilized 11 types of functional annotations from the IFmut database along with significant SNPs identified through genome-wide association studies (GWAS) (Supplementary Table 38 and Supplementary Table 39). The numbers outside the parentheses represent the number of annotated SNPs, and the numbers inside the parentheses represent the proportion of annotated SNPs across the genome.

**Type of annotation**	**Duroc**	**Yorkshire**
Significant SNPs	3,295(0.03%)	28,382(0.18%)
Open Chromatin Region	571,288(5.03%)	699,069(4.52%)
Nucleosome Free Region	160,332(1.41%)	189,292(1.22%)
footprint	33,515(0.30%)	38,272(0.25%)
matched_motif	1,660(0.01%)	1,584(0.01%)
intersect_motif	4,547(0.04%)	4,077(0.03%)
any_motif	199,328(1.76%)	229,737(1.49%)
active_promoter_narrowPeak	604,332(5.33%)	735,864(4.76%)
enhancer_narrowPeak	1,965,145(17.32%)	2,507,204(16.22%)
active_promoter	277,724(2.45%)	330,332(2.14%)
enhancer	277,724(2.45%)	1,587,620(10.27%)

Supplementary Table 34. The average number and proportion of SNPs with distinct functional annotations in the rice dataset. The annotation process utilized 9 types of annotations from the RGAP database along with significant SNPs identified through genome-wide association studies (GWAS) (Supplementary Table 40). The numbers outside the parentheses represent the number of annotated SNPs, and the numbers inside the parentheses represent the proportion of annotated SNPs across the genome.

Type of annotation	Rice
Significant SNPs	1,224(0.37%)
TE	27,240(8.17%)
expressed	95,619(28.68%)
representative	100,990(30.29%)
CDS	40,686(12.20%)
exon	51,701(15.50%)
5’ UTR	3,997(1.20%)
gene	101,772(30.52%)
3’ UTR	42,196(12.65%)

Supplementary Table 35. The number and proportion of SNPs with distinct functional annotations for 7 traits in the WTCCC1 dataset. The annotation process utilized 7 ranking scores of functional annotations from the RegulomeDB database. The numbers outside the parentheses represent the number of annotated SNPs, and the numbers inside the parentheses represent the proportion of annotated SNPs across the genome.

Type of annotation	Bipolar disorder (BD)	Coronary artery disease (CAD)	Crohn's disease (CD)	Hypertension (HT)	Rheumatoid arthritis (RA)	Type 1 diabetes (T1D)	Type 2 diabetes (T2D)
Rank 1	22,184 (5.94%)	22,170 (5.95%)	22,202 (5.93%)	22,145 (5.93%)	22,148 (5.94%)	22,001 (5.90%)	22,086 (5.92%)
Rank 2	10,611 (2.84%)	10,569 (2.84%)	10,673 (2.85%)	10,627 (2.85%)	10,595 (2.84%)	10,616 (2.85%)	10,603 (2.84%)
Rank 3	23,652 (6.33%)	23,532 (6.32%)	23,772 (6.35%)	23,570 (6.31%)	23,640 (6.34%)	23,627 (6.33%)	23,571 (6.32%)
Rank 4	61,545 (16.48%)	61,315 (16.46%)	61,738 (16.50%)	61,684 (16.52%)	61,643 (16.52%)	61,557 (16.50%)	61,645 (16.52%)
Rank 5	138,812 (37.18%)	138,502 (37.18%)	139,008 (37.16%)	138,802 (37.18%)	138,657 (37.17%)	138,758 (37.20%)	138,827 (37.20%)
Rank 6	41,345 (11.07%)	41,299 (11.09%)	41,439 (11.08%)	41,292 (11.06%)	41,292 (11.07%)	41,303 (11.07%)	41,310 (11.07%)
Rank 7	75,220 (20.15%)	75,154 (20.17%)	75,281 (20.12%)	75,218 (20.15%)	75,081 (20.13%)	75,102 (20.14%)	75,107 (20.13%)

Supplementary Table 36. The significant SNPs for seven disease traits in the WTCCC1 dataset. The general linear model was used to carry out the GWAS analysis (Supplementary Fig. 13). The numbers before the slash (/) represent the significant SNPs that exceed the threshold line, and the numbers after the slash represent all markers that are located within a 50kb interval upstream and downstream of the significant SNPs.

Trait[1] / Repetition	BD	CAD	CD	HT	RA	T1D	T2D
1	--/--	13/46	35/175	--/--	107/561	168/853	9/30
2	--/--	15/47	38/223	--/--	127/648	168/827	10/32
3	--/--	13/46	32/171	1/23	114/741	176/811	12/33
4	--/--	9/43	25/132	--/--	120/538	166/834	13/69
5	--/--	12/46	33/215	--/--	121/636	169/820	10/32
6	--/--	10/46	25/158	--/--	104/577	161/814	10/32
7	--/--	13/46	27/167	--/--	116/606	176/803	7/100
8	--/--	18/47	38/199	--/--	101/448	160/869	10/32
9	--/--	7/41	20/133	--/--	115/603	167/834	5/23
10	--/--	15/47	26/154	--/--	100/524	143/726	12/33
11	--/--	15/47	34/171	--/--	104/590	182/839	10/32
12	--/--	12/46	47/194	--/--	114/487	156/812	15/94
13	1/1	14/46	27/207	--/--	123/604	161/809	9/32
14	--/--	11/46	27/154	--/--	126/674	169/817	10/32
15	--/--	14/46	22/204	--/--	115/584	169/786	8/31
16	--/--	12/137	36/181	--/--	100/509	165/858	10/32
17	--/--	7/41	28/158	--/--	118/596	164/862	10/32
18	--/--	10/45	25/154	--/--	123/715	176/818	10/32
19	--/--	8/43	36/273	--/--	115/557	173/803	9/32
20	--/--	13/46	25/130	--/--	101/524	170/895	10/32

[1] BD: bipolar disorder; CAD: coronary artery disease; CD: Crohn's disease; HT: hypertension; RA: rheumatoid arthritis; T1D: type 1 diabetes; T2D: type 2 diabetes

Supplementary Table 37. The significant SNPs for six traits in the UKBiobank dataset. The linear regression model was used to carry out the GWAS analysis (Supplementary Fig. 14).

Trait[1] / Repetition	HT	BMR	hBMD	FVC	BMI	FEV
1	18,302	5,787	3,488	2,376	3,287	2,883
2	18,240	5,759	3,515	2,621	3,186	3,094
3	18,583	5,528	3,488	2,545	3,122	2,864
4	18,201	5,481	3,393	2,722	3,198	2,938
5	18,234	5,749	3,619	2,496	3,122	2,904
6	18,261	5,734	3,516	2,481	3,302	2,835
7	18,250	5,738	3,540	2,515	3,238	2,740
8	18,116	5,523	3,470	2,587	3,127	2,834
9	18,151	5,803	3,536	2,533	3,042	2,747
10	18,459	5,578	3,486	2,791	3,217	2,877

[1] HT: height; BMR: basal metabolic rate; hBMD: heel bone mineral density T-score; FVC: forced vital capacity; FEV: forced expiratory volume in 1s; BMI: body mass index

Supplementary Table 38. The significant SNPs for seven agricultural economic traits in the Duroc pig dataset. The mixed linear model was used to carry out the GWAS analysis (Supplementary Fig. 15). The numbers before the slash (/) represent the significant SNPs that exceed the threshold line, and the numbers after the slash represent all markers that are located within a 100kb interval upstream and downstream of the significant SNPs.

Repetition \ Trait[1]	BF	LMD	ELMP	LTN	RTN	TTN	TPD
1	204/6,709	--/--	73/5,528	258/936	501/1,507	879/5,164	12/1,647
2	200/7,637	--/--	102/4,958	258/936	513/2,129	590/3,061	8/1,507
3	202/6,703	--/--	69/4,975	254/936	519/2,890	667/3,067	5/1,266
4	198/7,075	--/--	43/3,974	679/2,952	499/1,507	694/3,297	9/1,509
5	207/6,703	--/--	46/3,982	537/2,154	503/1,915	603/2,837	12/1,647
6	206/6,703	--/--	95/5,988	506/1,785	510/2,544	686/3,067	5/1,266
7	205/6,893	--/--	18/2,354	504/1,569	516/2,154	684/3,065	6/1,287
8	226/7,080	--/--	75/3,993	257/936	517/2,383	585/3,067	7/1,503
9	200/6,703	--/--	116/6,111	257/936	500/1,507	692/4,001	5/1,013
10	208/8,119	--/--	23/2,007	500/1,507	510/2,546	581/2,998	9/1,613
11	202/6,710	--/--	83/5,942	534/1,920	512/2,761	661/3,067	2/622
12	218/8,588	--/--	55/3,959	258/936	511/2,153	684/3,069	7/1,272
13	193/6,703	--/--	33/3,974	517/1,920	504/1,737	664/3,015	3/622
14	204/6,703	--/--	133/6,156	663/2,203	512/1,922	610/2,413	4/1,025
15	205/6,703	--/--	11/2,715	257/936	517/2,993	676/3,063	8/1,465
16	196/7,830	--/--	22/2,624	257/936	509/1,922	680/3,067	14/1,600
17	219/7,646	--/--	44/3,097	500/1,455	511/2,138	706/3,076	9/1,552
18	215/7,650	--/--	72/4,682	654/1,961	566/3,008	687/3,072	2/622
19	269/8,112	--/--	97/5,166	606/1,960	512/1,922	516/2,361	16/1,649
20	210/7,075	--/--	59/4,654	509/1,838	535/3,255	684/3,069	--/--

[1] BF: backfat thickness; LMD: loin muscle depth; ELMP: estimated lean meat percentage; LTN: left teat number; RTN: right teat number; TTN: total teat number; TPD: time spent eating per day

Supplementary Table 39. The significant SNPs for seven agricultural economic traits in the Yorkshire pig dataset. The mixed linear model was used to carry out the GWAS analysis (Supplementary Fig. 16). The numbers before the slash (/) represent the significant SNPs that exceed the threshold line, and the numbers after the slash represent all markers that are located within a 100kb interval upstream and downstream of the significant SNPs.

Repetition \ Trait[1]	D100	BF100	LMA100	LTN	RTN	TTN
1	464/26,737	3,262/44,412	1,838/43,211	2,954/12,881	3,709/21,048	3,485/14,784
2	1,195/31,565	2,857/38,988	1,725/35,578	3,167/13,789	4,439/20,881	5,230/25,867
3	1,254/35,616	3,540/46,844	1,892/54,336	3,068/13,342	5,080/21,834	3,943/22,398
4	855/32,228	3,401/35,042	1,829/43,560	2,932/12,614	4,193/21,229	4,357/22,591
5	730/24,977	4,006/43,791	1,803/39,369	3,225/12,793	3,924/21,785	3,823/18,293
6	1,685/40,961	3,842/40,713	1,797/52,378	3,005/13,745	4,552/21,736	3,718/20,064
7	556/31,407	3,498/43,611	1,689/37,307	2,900/12,837	4,239/20,881	3,780/19,149
8	766/33,755	3,087/42,481	1,622/40,892	2,912/12,597	3,301/17,679	4,197/24,497
9	564/27,846	3,919/39,798	1,745/38,430	3,052/14,040	4,053/21,759	4,430/21,484
10	1,129/23,711	4,178/41,108	1,653/33,839	2,845/16,384	4,632/26,298	3,953/23,223
11	1,745/28,439	3,715/44,209	1,773/40,194	2,801/13,012	3,808/18,396	3,877/26,535
12	450/30,269	4,305/39,534	1,722/34,187	3,098/14,536	3,242/22,660	4,213/32,719
13	520/26,718	3,088/37,385	1,706/44,997	3,036/13,170	4,263/21,468	4,235/27,853
14	1,087/35,043	3,795/38,753	1,871/49,608	2,814/12,837	4,312/21,480	4,256/22,863
15	1,294/36,623	3,418/32,276	1,793/33,875	2,526/11,862	4,087/18,319	3,825/26,456
16	1,614/38,114	3,840/40,729	1,874/36,381	2,918/12,865	4,077/21,089	4,871/25,869
17	1,355/21,054	4,565/45,958	1,770/38,485	2,855/13,354	4,556/23,429	3,744/15,364
18	1,390/36,222	2,485/31,758	1,649/47,747	2,775/12,507	3,204/13,520	3,714/26,160
19	1,542/29,940	4,020/40,225	1,747/44,872	3,176/12,260	4,481/22,623	4,289/20,782
20	973/33,998	4,207/40,610	1,669/36,641	2,937/12,865	3,175/20,160	4,049/27,027

[1] D100: age adjusted 100 kg; BF100: backfat thickness adjusted to 100 kg; LMA100: loin muscle area adjusted to 100 kg; LTN: left teat number; RTN: right teat number; TTN: total teat number

Supplementary Table 40. The significant SNPs for five yield-related traits in the rice dataset. The mixed linear model was used to carry out the GWAS analysis (Supplementary Fig. 17). The numbers before the slash (/) represent the significant SNPs that exceed the threshold line, and the numbers after the slash represent all markers that are located within a 100kb interval upstream and downstream of the significant SNPs.

Trait[1] / Repetition	GL	GW	GLWR	DH	TGW
1	41/1,410	39/1,362	78/1,814	16/1,364	5/597
2	55/1,811	33/1,589	1,605/1,605	9/819	5/786
3	51/1,564	33/739	1,772/1,772	8/732	6/811
4	56/2,081	47/2,007	1,657/1,657	11/873	2/326
5	51/1,454	40/1,164	1,698/1,698	11/627	5/506
6	53/2,296	30/926	1,570/1,570	6/719	7/860
7	47/2,202	32/1,374	1,566/1,566	10/736	7/690
8	49/1,824	47/2,735	1,204/1,204	8/747	5/754
9	50/2,023	37/1,333	1,995/1,995	7/446	5/811
10	50/1,780	41/1,597	1,695/1,695	9/771	4/429
11	39/1,343	29/1,822	1,256/1,256	7/606	5/689
12	59/1,988	38/1,460	1,484/1,484	10/893	2/413
13	40/1,624	35/976	1,757/1,757	9/786	6/690
14	44/1,691	40/1,281	1,605/1,605	10/909	2/444
15	53/1,797	32/1,216	1,471/1,471	6/334	6/579
16	57/1,932	40/1,318	1,383/1,383	7/446	9/972
17	47/1,551	32/725	1,228/1,228	10/10	3/444
18	59/1,780	25/534	1,796/1,796	9/860	7/860
19	38/1,617	44/2,245	1,685/1,685	6/615	3/506
20	50/2,821	31/1,113	1,254/1,254	9/751	3/596

[1] GL: grain length; GW: grain width; GLWR: grain length-to-width ratio; DH: days to heading; TGW: thousand grain weight

Supplementary References

1. Goddard ME, Hayes BJ. Mapping genes for complex traits in domestic animals and their use in breeding programmes. *Nat. Rev. Genet.* **10**, 381-391 (2009).

2. Georges M, Charlier C, Hayes B. Harnessing genomic information for livestock improvement. *Nat. Rev. Genet.* **20**, 135-156 (2019).

3. Piepho HP. Ridge regression and extensions for genomewide selection in maize. *Crop Sci.* **49**, 1165-1176 (2009).

4. Henderson CR. Best linear unbiased estimation and prediction under a selection model. *Biometrics* **31**, 423-447 (1975).

5. VanRaden PM. Efficient methods to compute genomic predictions. *J. Dairy. Sci.* **91**, 4414-4423 (2008).

6. Moser G, Lee SH, Hayes BJ, Goddard ME, Wray NR, Visscher PM. Simultaneous discovery, estimation and prediction analysis of complex traits using a bayesian mixture model. *PLOS Genet.* **11**, 1-21 (2015).

7. MacLeod IM, Bowman PJ, Vander Jagt CJ, Haile-Mariam M, Kemper KE, Chamberlain AJ, Schrooten C, Hayes BJ, Goddard ME. Exploiting biological priors and sequence variants enhances QTL discovery and genomic prediction of complex traits. *BMC Genomics* **17**, 1-21 (2016).

8. Mollandin F, Gilbert H, Croiseau P, Rau A. Accounting for overlapping annotations in genomic prediction models of complex traits. *BMC Bioinformatics* **23**, 1-22 (2022).

9. Speed D, Balding DJ. MultiBLUP: improved SNP-based prediction for complex traits. *Genome Res.* **24**, 1550-1557 (2014).

10. Boyle AP, Hong EL, Hariharan M, Cheng Y, Schaub MA, Kasowski M, Karczewski KJ, Park J, Hitz BC, Weng S, Cherry JM, Snyder M. Annotation of functional variation in personal genomes using RegulomeDB. *Genome Res.* **22**, 1790-1797 (2012).

11. Finucane HK, Bulik-Sullivan B, Gusev A, Trynka G, Reshef Y, Loh P-R, Anttila V, Xu H, Zang C, Farh K, Ripke S, Day FR, Purcell S, Stahl E, Lindstrom S, Perry JRB, Okada Y, Raychaudhuri S, Daly MJ, Patterson N, Neale BM, Price AL, ReproGen C, Schizophrenia Working Group of the Psychiatric Genomics

C, The RC. Partitioning heritability by functional annotation using genome-wide association summary statistics. *Nat. Genet.* **47**, 1228-1235 (2015).

12. Ma R, Kuang R, Zhang J, Sun J, Xu Y, Zhou X, Han Z, Hu M, Wang D, Fu Y, Zhang Y, Li X, Zhu M, Zhao S, Xiang T, Shi M, Zhao Y. Annotation and assessment of functional variants in livestock through epigenomic data. *J. Genet. Genomics.* **52**, 1588-1599 (2025).

13. Hamilton John P, Li C, Buell CR. The rice genome annotation project: an updated database for mining the rice genome. *Nucleic Acids Res.* **53**, 1614-1622 (2024).
