# Supplementary material for: Improving genomic prediction accuracy of complex traits by integrating massive types of functional annotation information
Source: Nat Commun. 2026 Apr 24;17:5686. doi: 10.1038/s41467-026-72470-0 (PMC13319817; doi:10.1038/s41467-026-72470-0)
Supplement: Supplementary file 2 — Description of Additional Supplementary Files [file 41467_2026_72470_MOESM2_ESM.docx]

**File Name: Supplementary Data 1**

**Description:** The number and proportion of SNPs with distinct functional annotations for 7 traits in the WTCCC1 dataset. The annotation process utilized 74 types of functional annotations from the website of the LD Scores Regression (LDSC) model along with significant SNPs identified through genome-wide association studies. The numbers outside the parentheses represent the number of annotated SNPs and the numbers inside the parentheses represent the proportion of annotated SNPs across the genome.

**File Name: Supplementary Data 2**

**Description:** The estimates of genetic variance component were computed by GBLUP, IFAM, and a genomic prediction model that contains multiple random effects (MultiBLUP) using the WTCCC1 dataset. The MultiBLUP model contained 75 random effects, each of which was constructed using 74 functional annotations from the LD Scores Regression (LDSC) model and significant SNPs of trait identified through genome-wide association studies. The standard errors of the estimates are shown in parentheses.

**File Name: Supplementary Data 3**

**Description:** The average number and proportion of SNPs with distinct functional annotations in the UK Biobank dataset. The annotation process utilized 74 types of functional annotations from the website of the LD Scores Regression (LDSC) model along with significant SNPs identified through genome-wide association studies. The numbers outside the parentheses represent the number of annotated SNPs, and the numbers inside the parentheses represent the proportion of annotated SNPs across the genome.
