# Supplementary material for: Improving genomic prediction accuracy of complex traits by integrating massive types of functional annotation information
Source: Nat Commun. 2026 Apr 24;17:5686. doi: 10.1038/s41467-026-72470-0 (PMC13319817; doi:10.1038/s41467-026-72470-0)
Supplement: Supplementary file 6 — Reporting Summary [file 41467_2026_72470_MOESM6_ESM.pdf]

nature portfolio

Corresponding author(s): Lilin Yin, Shuhong Zhao, and Xiaolei Liu

Last updated by author(s): Mar 24, 2026

Reporting Summary

Nature Portfolio wishes to improve the reproducibility of the work that we publish. This form provides structure for consistency and transparency in reporting. For further information on Nature Portfolio policies, see our Editorial Policies and the Editorial Policy Checklist.

Statistics

For all statistical analyses, confirm that the following items are present in the figure legend, table legend, main text, or Methods section.

n/a	Confirmed	
☐	☒	The exact sample size (*n*) for each experimental group/condition, given as a discrete number and unit of measurement
☐	☒	A statement on whether measurements were taken from distinct samples or whether the same sample was measured repeatedly
☐	☒	The statistical test(s) used AND whether they are one- or two-sided<br>*Only common tests should be described solely by name; describe more complex techniques in the Methods section.*
☐	☒	A description of all covariates tested
☐	☒	A description of any assumptions or corrections, such as tests of normality and adjustment for multiple comparisons
☐	☒	A full description of the statistical parameters including central tendency (e.g. means) or other basic estimates (e.g. regression coefficient) AND variation (e.g. standard deviation) or associated estimates of uncertainty (e.g. confidence intervals)
☐	☒	For null hypothesis testing, the test statistic (e.g. *F*, *t*, *r*) with confidence intervals, effect sizes, degrees of freedom and *P* value noted<br>*Give P values as exact values whenever suitable.*
☐	☒	For Bayesian analysis, information on the choice of priors and Markov chain Monte Carlo settings
☐	☒	For hierarchical and complex designs, identification of the appropriate level for tests and full reporting of outcomes
☐	☒	Estimates of effect sizes (e.g. Cohen's *d*, Pearson's *r*), indicating how they were calculated

Our web collection on statistics for biologists contains articles on many of the points above.

Software and code

Policy information about availability of computer code

Data collection	The data are from public sources, as described in the Methods and Data Availability section.
Data analysis	The IFAM software code is available at: https://github.com/xiaolei-lab/IFAM, under the MIT license. Codes for the version of IFAM used in this paper are also deposited at Zenodo (https://doi.org/10.5281/zenodo.18802709).

For manuscripts utilizing custom algorithms or software that are central to the research but not yet described in published literature, software must be made available to editors and reviewers. We strongly encourage code deposition in a community repository (e.g. GitHub). See the Nature Portfolio guidelines for submitting code & software for further information.

Data

Policy information about availability of data

All manuscripts must include a data availability statement. This statement should provide the following information, where applicable:

- Accession codes, unique identifiers, or web links for publicly available datasets
- A description of any restrictions on data availability
- For clinical datasets or third party data, please ensure that the statement adheres to our policy

All data used in this study are publicly available. Individual-level genotype and phenotype data for height, basal metabolic rate, heel bone mineral density T-score, forced vital capacity, body mass index, and forced expiratory volume in 1s from the UK Biobank (2017 release) were obtained under Application Number 97563

(https://www.ukbiobank.ac.uk/enable-your-research/apply-for-access). The human case-control data were obtained from the WTCCC1 study, specifically covering seven diseases: bipolar disorder, coronary artery disease, Crohn's disease, hypertension, rheumatoid arthritis, type 1 diabetes, and type 2 diabetes; access to these data is managed by the Wellcome Trust Case Control Consortium (http://www.wtccc.org.uk/). The Duroc pig dataset (low coverage whole-genome sequencing and agricultural economic traits) is available via GigaDB at http://dx.doi.org/10.5524/100894. The Yorkshire pig genotype and six production traits52 generated in this study have been deposited in Figshare (https://doi.org/10.6084/m9.figshare.30985417). The 3,000 Rice Genomes Project SNP arrays and phenotype records are accessible at https://iric.irri.org/projects/3000-rice-genomes-project. Humans functional annotations were obtained from the RegulomeDB database (https://www.regulomedb.org/regulome-search) and the LD Scores Regression model (https://alkesgroup.broadinstitute.org/LDSCORE/baselineLD_v2.2_bedfiles.tgz/). Pig functional annotations are available from the IFmut database (http://www.ifmutants.com:8212/#/download). Rice genomic annotations were obtained from the Rice Genome Annotation Project Database (https://rice.uga.edu/pub/data/Eukaryotic_Projects/o_sativa/annotation_dbs/pseudomolecules/version_7.0/all.dir/). Source data are provided with this paper.

Research involving human participants, their data, or biological material

Policy information about studies with human participants or human data. See also policy information about sex, gender (identity/presentation), and sexual orientation and race, ethnicity and racism.

Reporting on sex and gender	All data we access from previous studies that detail the sex and gender. The data and the agreement numbers have been acknowledged in the manuscript.
Reporting on race, ethnicity, or other socially relevant groupings	All data we access from previous studies that detail the race, ethnicity, or other socially relevant groupings. The data and the agreement numbers have been acknowledged in the manuscript.
Population characteristics	All data we access from previous studies that detail the population characteristics. The data and the agreement numbers have been acknowledged in the manuscript.
Recruitment	All data we access from previous studies that detail the data recruitment. The data and the agreement numbers have been acknowledged in the manuscript.
Ethics oversight	All research was conducted in accordance with the relevant guidelines and the criteria set by the Declaration of Helsinki. This study involved the secondary analysis of de-identified, publicly available data from the UK Biobank and the WTCCC. The UK Biobank received ethical approval from the North West Haydock Research Ethics Committee (reference no. 21/NW/0157), and all participants provided informed consent. The WTCCC project was approved by the South East Multicenter Research Ethics Committee (reference no. 05/Q0106/74), and all participants provided written informed consent. The Animal Ethics Committee of Huazhong Agricultural University granted approval for the experimental designs and procedures (approval no: [HZAUSW20260016]). Rigorous measures were undertaken to assure the well-being and humane handling of the Yorkshire pigs engaged in this study. The gathering of specimens and data was executed in conformity with the pertinent regulations and directives on animal welfare and protection.

Note that full information on the approval of the study protocol must also be provided in the manuscript.

Field-specific reporting

Please select the one below that is the best fit for your research. If you are not sure, read the appropriate sections before making your selection.

☒ Life sciences ☐ Behavioural & social sciences ☐ Ecological, evolutionary & environmental sciences

For a reference copy of the document with all sections, see nature.com/documents/nr-reporting-summary-flat.pdf

Life sciences study design

All studies must disclose on these points even when the disclosure is negative.

Sample size	The WTCCC1 dataset consists of seven binary disease traits, namely bipolar disorder (N=4,806), coronary artery disease (N=4,864), Crohn's disease (N=4,686), hypertension (N=4,890), rheumatoid arthritis (N=4,798), type 1 diabetes (N=4,901), and type 2 diabetes (N=4,862);<br>The UK Biobank included six representative traits with diverse genetic architectures (from less to highly polygenic), namely height (N=318,324), basal metabolic rate (N=313,495), heel bone mineral density T-score (N=181,028), forced vital capacity (N=291,054), body mass index (N=317,989), and forced expiratory volume in 1s (N=291,054);<br>The Duroc pig dataset comprised the seven available traits, namely backfat thickness (N=2,771), loin muscle depth (N=2,789), estimated lean meat percentage (N=2,782), left teat number (N=2,796), right teat number (N=2,796), total teat number (N=2,796), and time spent eating per day (N=2,600);<br>The Yorkshire pig dataset included six traits closely related to production performance, namely the age adjusted to 100 kg (N=16,732), backfat thickness adjusted to 100 kg (N=16,673), eye muscle area adjusted to 100 kg (N=15,852), left teat number (N=13,726), right teat number (N=13,726), and total teat number (N=13,726);<br>The rice dataset comprised the five representative yield-related traits, namely grain length (N=2,011), grain width (N=2,011), grain length-to-width ratio (N=2,011), days to heading (N=2,718), and thousand grain weight (N=1,787).The detailed information was described in the Methods section.
Data exclusions	The genotypic data from the WTCCC1 dataset encompassed approximately 450,000 SNPs per individual. Genotypic data quality control was conducted as described previously. Briefly, SNPs were excluded using PLINK software (v1.90) based on the following criteria: minor allele frequency (MAF) < 0.01, genotype call rate < 0.95, and P-value < 0.05 from the Hardy-Weinberg equilibrium test. The detailed information on the remaining data was shown in the Supplementary Table 21;

The UK Biobank data processing followed the procedure described in the study by Lloyd-Jones et al.. Briefly, the HapMap3 SNPs, genotyped in 2504 participants of the 1000 Genomes Project, were extracted from the UKB dataset, the SNPs with MAF > 0.01, probability of departure from Hardy-Weinberg equilibrium < 10^(-6), and missingness < 0.05 were retained, which resulted in 1,094,840 SNPs. Genotypic principal components were used to infer ancestry and subpopulation structure, and a subset of 348,501 unrelated (absolute genomic relationship matrix off-diagonal < 0.05) individuals of Europe was selected for analysis;

The Duroc pig dataset comprised genotypic and phenotypic information from 2,797 boars with 11,348,460 SNPs. Quality control was applied to the imputed genotypic data by filtering out SNPs with MAF < 0.01, genotype call rate < 0.90, and individual call rate < 0.90 using Plink software (v1.90). A total of 11,348,241 SNPs were retained for all 2,796 pigs;

A total of 16,783 Yorkshire pigs were recruited from a commercial breeding farm and all pigs were raised under standardized husbandry conditions. Tail tissue samples were collected from each pig during the routine tail-docking procedure for piglets. This non-invasive integration into standard farm management practices minimized additional stress to the animals. Immediately after collection, the tail tissues were preserved in 75% ethanol and stored at -20°C to maintain DNA integrity. All preserved samples were transported to Yingzi Gene Technology Co., Ltd. (Wuhan, China) for genomic analysis. Genomic DNA was extracted from the tail tissues using standard protocols. High-throughput genotyping was subsequently performed using the pig 80K functional variants genotyping array. The genotype data with 187,000 SNPs were imputed using the reference panel and the imputation tool from the AGIDB website (http://animalbreedinglab.hzau.edu.cn/AGIDB/home/home.php), and variants with imputation accuracy R2 > 0.7 were retained. Following the same quality control protocols applied to the Duroc pig dataset, a total of 15,457,739 SNPs were reserved for analysis;

The rice data included 3,024 rice accessions. Quality control was applied to the genotype data by filtering out SNPs with MAF < 0.01, genotype call rate < 0.90, and individual call rate < 0.90 using Plink software (v1.90), and a total of 333,454 SNPs were retained.

Detailed quality control procedure for the analyzed data can be found in the Methods section.

Replication

Computational Validation: For the genomic prediction models (IFAM and benchmarks), we employed 10 times 80%/20% random splits for UK Biobank dataset and 20 times 80%/20% random splits for other datasets using different random seeds to ensure the stability and reproducibility of the results.

Independent Datasets: The model's performance was independently verified across five distinct datasets (UK Biobank, WTCCC1, Duroc pig, Yorkshire pig, and rice), treating each population as an independent biological replication of the method's efficacy.

Statistical Consistency: All reported metrics (e.g., Pearson’s correlation) represent the mean values derived from these independent runs, with the variations provided as standard errors.

The breadth of scenarios and real data analyses are sufficient, we believe, evidence for reviewers to assess these conclusions.

Randomization

All randomization information, if available, were described in the original paper for the used data sets. The other methods used for comparison were run with default parameters and random seeds, as described in the original paper for the used datasets.

Blinding

Blinding in population data collection is not concern as no treatment is being investigated.

Reporting for specific materials, systems and methods

We require information from authors about some types of materials, experimental systems and methods used in many studies. Here, indicate whether each material, system or method listed is relevant to your study. If you are not sure if a list item applies to your research, read the appropriate section before selecting a response.

Materials & experimental systems

n/a	Involved in the study
☒	☐ Antibodies
☒	☐ Eukaryotic cell lines
☒	☐ Palaeontology and archaeology
☒	☐ Animals and other organisms
☒	☐ Clinical data
☒	☐ Dual use research of concern
☒	☐ Plants

Methods

n/a	Involved in the study
☒	☐ ChIP-seq
☒	☐ Flow cytometry
☒	☐ MRI-based neuroimaging

Plants

Seed stocks	*Report on the source of all seed stocks or other plant material used. If applicable, state the seed stock centre and catalogue number. If plant specimens were collected from the field, describe the collection location, date and sampling procedures.*
Novel plant genotypes	*Describe the methods by which all novel plant genotypes were produced. This includes those generated by transgenic approaches, gene editing, chemical/radiation-based mutagenesis and hybridization. For transgenic lines, describe the transformation method, the number of independent lines analyzed and the generation upon which experiments were performed. For gene-edited lines, describe the editor used, the endogenous sequence targeted for editing, the targeting guide RNA sequence (if applicable) and how the editor was applied.*
Authentication	*Describe any authentication procedures for each seed stock used or novel genotype generated. Describe any experiments used to assess the effect of a mutation and, where applicable, how potential secondary effects (e.g. second site T-DNA insertions, mosiacism, off-target gene editing) were examined.*
