# Supplementary material for: Improving genomic prediction accuracy of complex traits by integrating massive types of functional annotation information
Source: Nat Commun. 2026 Apr 24;17:5686. doi: 10.1038/s41467-026-72470-0 (PMC13319817; doi:10.1038/s41467-026-72470-0)
Supplement: Supplementary file 7 — Transparent Peer Review file [file 41467_2026_72470_MOESM7_ESM.pdf]

nature portfolio

Peer Review File

Improving genomic prediction accuracy of complex traits by integrating massive types of functional annotation information

Corresponding Author: Professor Xiaolei Liu

This file contains all reviewer reports in order by version, followed by all author rebuttals in order by version.

Version 0:

Reviewer comments:

Reviewer #1

(Remarks to the Author)
Remarks to the Author:
Tang et al. proposed a new adaptive model, IFAM (Integrating Functional Annotations by Multi-random effect model), aiming to achieve accurate and efficient genomic prediction. IFAM extends the genomic best linear unbiased prediction (GBLUP) model into a multi-random effect form to accommodate multiple types of functional annotations. Compared with the baseline GBLUP model, IFAM achieved significant improvements in prediction accuracy across 20 traits from different datasets. However, the current study still has some shortcomings, mainly in the following aspects:

1. The description of the cross-validation strategy is inconsistent and not rigorous. In the Methods section, it is stated that the WTCCC1 and pig datasets used "20 repetitions of 80%/20% random splits," and the UKB dataset used "10 repetitions of 80%/20% random splits". However, in the Results section and figure captions, this is described as "20/10-fold cross-validation". It should be noted that repeated random splitting is not equivalent to standard k-fold cross-validation: the former may lead to overlapping validation sets and some samples never being included in validation. Referring to it as "k-fold" may mislead readers and hinder comparability with studies using the standard k-fold method.

2. The paper uses both AUC (qualitative trait) and Pearson r (quantitative trait) as accuracy metrics, but in the abstract/results it reports "average improvement" for each dataset and in one place gives "an average improvement of 6.86% across 20 traits". Since AUC and r differ in scale and upper bounds, directly averaging or comparing their percentage improvements is not statistically valid and may be misleading.

3. The pseudo-annotation control design may be affected by confounders such as MAF and LD distance. The paper only states "random selection of the same category and quantity", but does not clarify whether these distributions were matched. Without such matching, the conclusion that real annotations outperform pseudo-annotations may be partly driven by distributional differences. It is recommended to add MAF/LD-matched controls to strengthen causal interpretability.

4. The baseline setup is unbalanced, which affects the fair assessment of the method's innovation. The WTCCC1 dataset was compared with BayesR, BayesRC, and MultiBLUP, but the UKB dataset lacks comparisons with mainstream methods such as SBayesRC, LDpred-funct, and AnnoPred.

5. The authors mention in the introduction and discussion the applicability of the IFAM method to plant and animal breeding. However, the current study only validates the method on human and animal datasets, with no plant data included. Given that plant genomic prediction is more complex (e.g., polyploidy, high heterozygosity, and genotype–environment interactions), it is recommended that the authors add validation on plant datasets.

6. In the discussion, the authors mention comparing the IFAM model with the enhanced version of BayesRC, namely BayesRCO. However, the results do not present any findings related to BayesRCO. It is recommended that the authors provide further clarification, or explicitly include BayesRCO results in the Results section to ensure consistency.

7. In the IFAM model, it is not clear whether performance necessarily improves as the number of annotation types increases.

Since functional annotations are categorizations of SNP features rather than SNPs themselves, and SNPs are often assigned to multiple categories, such overlaps could affect variance component estimation and model stability. Substantial overlap among annotations might also increase the risk of overfitting. It is recommended that the authors address these points in the Methods or Discussion section.

8. The authors report the computational efficiency of the IFAM model under different numbers of random effects, but do not compare it with that of other models. It is recommended to include such comparisons to more clearly demonstrate the advantages of the proposed method.

9. The LDAK software is mentioned without providing the relevant reference; it is recommended that the authors add the appropriate citation.

10. In Figure 6, the legend label "IFAM_KMAL" should be corrected to "IFAM_KAML".

11. The sentence "All authors reviewed and approval the final manuscript" should be revised to "approved".

(Remarks on code availability)

Reviewer #2

(Remarks to the Author)
Review report

The manuscript proposes a model (IFAM) to integrating functional annotations for genomic prediction. The IFAM is a typical linear mixed model with multiple genetic components. Instead of treating each annotation type as one genetic component, IFAM merges the annotation types with similar variance of SNP effect to be one genetic component in the model. The model together with regular GBLUP and some other models were used for genomic prediction by integrating functional annotation information. The results showed that functional annotation information can improve accuracy of genomic prediction. Merging annotation types with similar variance to be a genetic component in the model is a good strategy. In addition, the results from the analysis on the contributions of different annotation categories to genetic variation of complex traits indicate the importance of functional annotation information. As the whole, it is a good study and provides valuable method and information for the improvement of genomic prediction.

The functional genomic annotation information used in this study were obtained from the website of the LD Scores Regression (LDSC) model, RegulomeDB database, and IFmut database (pig), and the data in the analysis were obtained from Welcome Trust Case-Control Consortium (WTCCC), UK Biobank, and a pig dataset (source not provided). These data appear suitable for this study. It will be helpful to describe how to choose the six traits from UK Biobank and the seven traits from the pig dataset in this study.
Various models are used for genomic prediction based on the above data sets. In general, the analysis methods are reasonable, but the descriptions of some scenarios need to be more detailed. See detailed comments below.
The results are relevant. However, the scenarios/models/datasets on which the results are based are not clearly described, so that it is a bit difficult to distinguish the approaches and compare the results. See detailed comments below.
The discussion connects well with the results. The conclusions are well supported by the results. However, the discussion is somewhat superficial and needs to be strengthened. For example, it would be helpful to discuss the strategy for merging functional annotation types (which may be different in different situations), the results that BayesRC which use annotation prior performs worse than BayesR which do not use annotation prior, and so on.
The following are the detailed comments. Due to the lack of line numbers in the manuscript, it may not be clear where the comments correspond to the text.

Introduction

Page 2: “Genomic prediction, also known as genomic selection”. It is not really correct. Genomic selection relies on genomic prediction but includes other relevant things within breeding programs.
Page 2: “challenges persist …”. Dependency on LD is not the only challenge. Even when using sequencing data that includes all variants in the genome, challenges remain. The same comment on “bypass the LD issues” in page 3. It is also not clear to what the authors refer with “functional annotations”. Also, “Therefore” does not connect the two sentences, since one still does not know which traits the QTNs affect, even with functional annotations.
Page 4. “the groups of genetic markers with similar contributions to phenotypic variance were automatically merged into one single random effect during variance assessment”. Grouping single genetic markers or grouping functional annotation types?
Compared statistical methods can be mentioned here. Some methods are listed in the introduction but they are not the ones used for comparison. What are their drawbacks, how IFAM differs from them, and what it solves in practice. Why we need IFAM?

Methods and data

Page 21: There are a large number of traits with phenotypic measurements in the UK Biobank, and there may be also a lot of traits in the pig dataset. How you choose some of these traits in this study?
Page 21, WTCCC1 dataset: "a p-value < 0.05 from the Hardy-Weinberg equilibrium test". Why p<0.05 instead of 10^-6 as used in UK Biobank dataset? Furthermore, the control:case ratio in the dataset was approximately 3:2, which is not a random sample (the ratio of disease is much higher than the true incidence), so the Hardy-Weinberg equilibrium test is invalid. How many SNPs were left out of 450K?
Page 22, "unrelated individuals of European ancestry was selected….". Unrelated how?
Page 22, UK Biobank dataset: "filtered based on …". Use either the removed criteria or the retained criteria, but not a mixture.
Page 22, Pig dataset: "low-coverage". How low? Explain why quality control criteria for MAF and call rate are different from those used in the WTCCC1 dataset and UK Biobank dataset.
Page 24, multiple random effect model, and elsewhere: "Mu is a vector of genetic effects". Using other letter (e.g., u) to represent random effect, because mu usually represents overall mean or intercept in a model.
Page 24: "additive marker covariates matrix". What does "additive" mean here?
Page 24: "sigma_gk^2 is the estimated genetic variance of ***Gk***". It should be "the genetic variance accounted for by SNPs in the kth annotation set.
Page 25: "with approximate variance component (within 10X)". 1) Need to point out 'similar variance per-SNP'. 2) Explain why it is 10x, is it optimal?
Page 25: "potentially addressing the convergence problem". The automated step of merging random components should be detailed. How do you estimate variance for each annotation type before merging them? How to avoid convergence issue, if a multiple random genetic effect model that includes all annotation types is used to estimate the variance of each annotation type simultaneously? The same comment on Page 26, "…iterative convergence…". The equation is not self-explanatory.
Page 25: "We first ordered the variances of multiple random effects". Variance of the annotation type or variance per-SNP in the annotation type? Similar to above.
Page 26, Evaluating functional annotations: "we used the variance ……contribution of functional annotations". 1) By definition, variance of SNP effect is sigma_a^2/sum of 2pq. 2) Are the SNPs used in estimating variance components and genomic predictions also pruned? Should they be kept consistent?
Page 26: "To conduct this analysis, we used a multiple random effects GBLUP model". all annotation types simultaneously? Maximum number of components? Problem with convergence?
Page 27: "ranking scores of SNPs from the RegulomeDB database". But RegulomeDB database has already grouped SNPs by ranking score.
Page 27, Cross-validation and prediction accuracy: It is mentioned that For UKB dataset, GWAS were carried out using Linear regression model. For WTCCC and pig data, are the GWAS results used in genomic prediction from GLM model or MLM model?
Page 27: "The predictive ability using Pearson's correlation". How about the bias of prediction?

Results

Results may be restructured. The data sets were already discussed partially in the first two subsections (Annotated results of three datasets, The importance of functional annotations), but then there are separate sections for each.
Page 5: "we found that the top five annotation covering …". Need to mention that the results are based on WTCCC data. Have you inspected the distribution of the LDSC annotation types in UKB data?
Page 6, Fig 1: "model by incorporating trait specific multiple random effects". Include also random effects of annotation types from the annotation datasets?
Page 6, Fig 1: A discussion or explanation on the big difference in these proportions between WTCCC1 and UKB datasets.
Page 7: "we accessed the difference". "accessed" should be "assessed". The mistake appears many places throughout the manuscript.
Page 7: "heritability of each category decreased gradually". This is a trend, not for all points. For example, Rank 4 has a larger variance than Rank 3 in 5 of 6 UKB traits.
Page 7: "Besides, the findings also indicated that eQTL information ……such as DNase footprints and motifs". Mention which table or figure shows these results.
Page 8: "different ranking scores in the WTCCC1 dataset ". Did you also do the assessment in UKB dataset?
Page 8: "The higher the ranking score, the lower was …". This is a trend, not for all points. For example, e.g., Rank 4 is higher than Rank 3, Rank 5 lower than rank 6 and 7.
Page 9 : "nearly 100 types. Present in detail, is LDSC 74 + RDB 7 + GWAS 1?
Page 9: "GRMs based on the position information…". How did you use the position information to construct GRMS?
Page 9: "the GBLUP model that contained multiple random effects". Did you estimate variance components for each annotation component simultaneously using a model containing about 100 random genetic components/GRMs? No problem with convergence?
Page 9: Finally, how many genetic components (or GRMs) are in IFAM model? Are the classes in BayesRC the same as the genetic components in IFAM?
Page 10, Fig. 3: "with two sets of functional annotations". Include also GWAS information?
Page 10, Fig. 3: BayesRC use both LDSC and RegulomeDB annotations or one of the two? What are the possible reasons that BayesRC which uses annotation prior performs worse than BayesR which does not use annotation prior.
Page 10, Fig.3: "20-fold cross-validation". It is not 20-fold cross validation. For 20-fold cross validation, the whole data should be divided into 20 subset, a subset is used as a validation set. TThis comment also applies elsewhere related to 20-fold cross validation.
Page 10, Fig. 3: It is helpful to describe how to apply annotation information in the adaptive multiblup model. Did you have the results from a regular multi-genetic component GBLUP model (e.g., GBLUP model with 7 annotation types from

RegulomeDB as 7 genetic components)?
Page 10: “the IFAM model using another set … from the RegulomeDB database”. Clarify what set is it?
Page 10: “highly consistent (Fig.3, Supplementary Table 14)”. No results from another set can be seen in Fig.3 and Supplementary Table 14. This paragraph is confusing with the next one.
Page 11: “conventional annotation information was slightly better than …”. Indicate the table/figure which show the difference in prediction accuracy between the two approaches.
Page 13, Fig. 4a: What is the base/reference for calculating the improvement in accuracy of IFAM_Rank and IFRAM_KAML?
Page 13：“functional annotation information from the RegulomeDB database”. It would be helpful to explain why some prediction model used both LDSC and RegulomeDB annotations, and the other used only RegulomeDB annotation.
Page 9-14: It is not clear what models used GWAS information.
Page 14: “using 10-fold cross-validation”. The same comment as on 20-fold cross-validation above.
Page 15: After merging annotation types, how many genetic components (GRMs) in the three IFAM models?
Page 15, Fig.6: “which included 10 types of annotations”. Include also GWAS information?
Page 9-15: For regulomeDB (maybe also IFmut), there are only 7 types of annotations, a regular multiple genetic component model may perform well. It may be worth evaluating.

Discussion

In this study, some prediction models used both LDSC and RegulomeDB annotations, and the other used only RegulomeDB annotation. It is expected that IFAM may have larger advantage when using LDSC annotation information than using RegulomeDB annotations, because there are 74 annotation types in LDSC and only 7 on RegulomeDB, the effect of merging annotation types would be more meaningful for LDSC annotations. It is worth discussing under what circumstances IFAM can lead to more improvements in genome prediction compared with other models. In addition, it is helpful to discuss the issue that BayesRC which use annotation prior performs worse than BayesR which do not use annotation prior.
Page 17: “the BayesRCO (enhanced version of BayesRC)……”. In the RESULT section, there are results from BayesRC, not BayesRCO.
Page 18: “automatically merging based on variance”. discussion on merging strategy is necessary. For example, a 10x variance is optimal? How many components in IFAM are appropriate? Merging strategy dependent on data?

Supplementary information

Page 2: “one random genetic effect (mu)”. Mu usually represents overall mean or intercept in a model, such as represented in the Bayesian model in this file.
Page 2: “b is a vector of the estimated effects of fixed covariates”. The description is not appropriate. b is the vector of fixed effects, not “estimated” and include effect for both categorical variables and continuous covariates.
Page 2: “BayesR … using the WTCCC1 dataset”. Explain why not also using the UKB dataset.
Page 3: Why the term of fixed effect is different from that in GBLUP model?
Page 3: “where the MCMC chain length was set to 10,000, and the burn-in step was set to 4,000”. The total chain length and the samples for burn-in seem very small. For both BayesR and BayesRC, did you inspect convergence, mixing and effective sample size?
Page 3: “see the study of MacLeod et al”. Provide the reference.
Page 5, Supplementary Fig.1: Need to point out that the results are based on WTCCC1 data. The colors should be consistent with those in Fig. 1 in the main manuscript. This figure does not provide more information beyond Fig.1 in the manuscript since the samples for different traits are almost identical.
Page 5, Supplementary Fig.2: Need to point out that the results are based on WTCCC1 data. Figure b does not provide more information beyond Figure a since the samples for different traits are almost identical.
Page 10: “from the IFAM model (no optimization of random effects)”. Is it a regular multiple genetic component GBLUP model?
Page 18, Supplementary Table 7: Need to point out that the results are based on WTCCC1 data.
Page 24-25, Supplementary Table 12 and 13: Provide SE of the estimates. What does “—” in Table 7 mean?
Page 26, Supplementary Table 14: ”using functional annotation information”. Using also GWAS information?
Page 27, Supplementary Table 15: Column IFAM is actually the same as IFAM_LDSC in Supplementary Table 14. The model name should be consistent across tables.
Page 26-29, Supplementary Table 14-17: It is not 20-fold cross validation (10-fold cross validation). The SE in your case is an artificial product. You can repeat the cross validation in an unlimited number of times, for example 10,000 replicates, then the SE will be close to zero.
Page 24-25, Supplementary Table 12 and 13: Provide SE of the estimates. What does “—”mean?
Page 31, Supplementary Table 19: The title is the same as Supplementary Table 12.
Page 33, Supplementary Table 21: The table provides the same information as Supplementary Fig. 8.

(Remarks on code availability)

Reviewer #3

(Remarks to the Author)
I co-reviewed this manuscript with one of the reviewers who provided the listed reports. This is part of the Nature

Communications initiative to facilitate training in peer review and to provide appropriate recognition for Early Career Researchers who co-review manuscripts.

(Remarks on code availability)

Reviewer #4

(Remarks to the Author)
The study presents a thoughtful framework that leverages functional annotations to improve genomic prediction and tests robustness across annotation sources. However, gaps in clarity regarding design and description, as well as limited benchmarking, make it difficult to assess the generality and comparative advantage of the approach.

The method provides thoughtful attempt to leverage biological annotations for prediction, and effort to test robustness by using alternative or random annotation sets.

The core modeling strategy, multiple random effects/annotation-partitioned variance components, is not new. Please clarify the incremental contribution beyond existing multi-component BLUP/Bayesian frameworks and related annotation-aware methods.

Although the paper emphasizes potential applications in both animals and plants, only one pig dataset with a relatively small sample size (a few thousand animals) is analyzed. In large populations with small effective population sizes (and therefore stronger LD), the benefits of using annotations may be minimal. A careful evaluation with larger datasets is needed.

For the human datasets, trait-specific significant SNPs from association analyses are used. Does this risk contaminating the testing dataset?

It appears that only distinct annotations are included in the analysis. Since many annotations are biologically overlapping, this could pose convergence issues. Was annotation overlap evaluated? The conclusion “It performed well for traits with both high heritability and low heritability, and it was also suitable for any type of functional annotations” seems overly broad.

The claim that “compared with Bayesian models that assume complex variances for markers, the IFAM model, which integrated biological prior knowledge, exhibited higher computational efficiency and predictive performance” requires further justification.

For the UKB dataset, why was GBLUP the only benchmark? Could the differences observed simply reflect the use of annotations?Although the comparisons demonstrate that the proposed model outperforms GBLUP, which is essentially a special case of their model, the lack of benchmarking against other advanced prediction methods weakens the persuasiveness of the results. For all benchmarks, are the reported accuracy improvements statistically significant?

One strength is the robustness test using random annotations. However, it remains unclear how much of the observed improvement is driven by GWAS SNPs versus other annotation sources. The paper repeatedly states that GWAS SNPs boost accuracy, but it does not present results using only GWAS SNPs as the annotation. Without that control, it is impossible to determine whether most of the gain comes solely from GWAS hits or whether other annotations provide meaningful additional value.

(Remarks on code availability)

Version 1:

Reviewer comments:

Reviewer #1

(Remarks to the Author)
The authors responded in a detailled way to the reviewer comments.

(Remarks on code availability)

Reviewer #2

(Remarks to the Author)
The manuscript has been improved significantly. Comments from me and the co-reviewer have been considered in the revised manuscript. We have only one comment remaining.
To validate model performance, the whole dataset was randomly split into training set and validation set, which were repeated 10 (or 20) times to produce 10 (or 20) replicates. However, a standard paired t-test is inappropriate for comparing

models based on these replicates. Because all replicates are derived from the same finite dataset, their validation sets overlap, making the replicates non-independent. This artificially inflates statistical significance as the number of replicates increases. Therefore, an alternative validation method or a corrected paired t test is required for the validation of model prediction ability.

(Remarks on code availability)

Reviewer #3

(Remarks to the Author)
I co-reviewed this manuscript with one of the reviewers who provided the listed reports. This is part of the Nature Communications initiative to facilitate training in peer review and to provide appropriate recognition for Early Career Researchers who co-review manuscripts.

(Remarks on code availability)

Reviewer #4

(Remarks to the Author)
My remaining concern is the updated analysis using two random effects (GWAS-significant SNPs vs. all remaining SNPs). In this setting, adding functional-annotation data provides minimal gains in livestock (+0.01%, +0.08%) and slightly lower accuracy in crops (−0.12%). As the authors note, this may be expected, and I appreciate the extensive benchmarking, however, I suggest stating this limitation clearly in the abstract.

(Remarks on code availability)

Version 2:

Reviewer comments:

Reviewer #2

(Remarks to the Author)
My comments have been considered and properly addressed in the revision. I have no further comments on the revised manuscript.

(Remarks on code availability)

Open Access This Peer Review File is licensed under a Creative Commons Attribution 4.0 International License, which permits use, sharing, adaptation, distribution and reproduction in any medium or format, as long as you give appropriate credit to the original author(s) and the source, provide a link to the Creative Commons license, and indicate if changes were made.
In cases where reviewers are anonymous, credit should be given to 'Anonymous Referee' and the source.
The images or other third party material in this Peer Review File are included in the article's Creative Commons license, unless indicated otherwise in a credit line to the material. If material is not included in the article's Creative Commons license and your intended use is not permitted by statutory regulation or exceeds the permitted use, you will need to obtain permission directly from the copyright holder.
To view a copy of this license, visit https://creativecommons.org/licenses/by/4.0/

NCOMMS-25-55562: Responses to Reviewers' Comments

Dear Reviewers,

Thank you for reviewing our manuscript (NCOMMS-25-55562) entitled "*IFAM: Improving genomic prediction accuracy of complex traits by integrating massive types of functional annotation information*". We highly appreciate the time and effort that you have dedicated to providing your valuable feedback on our manuscript. We are grateful for your insightful comments and professional suggestions. We have incorporated changes to reflect the suggestions and have highlighted the changes within the manuscript. Below, we provide point-by-point responses to your comments and concerns; your comments are in black regular font, while our responses are in *blue italics*.

Responses to Reviewer #1:

Remarks to the Author:
Tang et al. proposed a new adaptive model, IFAM (Integrating Functional Annotations by Multi-random effect model), aiming to achieve accurate and efficient genomic prediction. IFAM extends the genomic best linear unbiased prediction (GBLUP) model into a multi-random effect form to accommodate multiple types of functional annotations. Compared with the baseline GBLUP model, IFAM achieved significant improvements in prediction accuracy across 20 traits from different datasets. However, the current study still has some shortcomings, mainly in the following aspects.
Response: *We sincerely thank you for your positive assessment and professional suggestions.*

1. The description of the cross-validation strategy is inconsistent and not rigorous. In the Methods section, it is stated that the WTCCC1 and pig datasets used "20 repetitions of 80%/20% random splits," and the UKB dataset used "10 repetitions of 80%/20% random splits". However, in the Results section and figure captions, this is described as "20/10-fold cross-validation". It should be noted that repeated random splitting is not equivalent to standard k-fold cross-validation: the former may lead to overlapping validation sets and some samples never being included in validation. Referring to it as "k-fold" may mislead readers and hinder comparability with studies using the standard k-fold method.

Response: *Thank you for pointing out the issue. The initial description of the cross-validation strategy was indeed confusing. All the model evaluations and comparisons in our manuscript were conducted using a random splitting strategy, thus we have revised the manuscript to consistently refer to the approach as "repetitions" rather than "k-fold cross-validation" throughout the Results section and figure captions.*

2. The paper uses both AUC (qualitative trait) and Pearson r (quantitative trait) as accuracy metrics, but in the abstract/results it reports "average improvement" for each dataset and in one place gives "an average improvement of 6.86% across 20 traits". Since AUC and r differ in scale and upper bounds, directly averaging or comparing their

percentage improvements is not statistically valid and may be misleading.

Response: *Thank you for raising this critical point. We agree that directly averaging percentage improvements across different metrics, such as AUC and Pearson r, can be misleading due to their differing scales and properties. We have revised the manuscript to address this concern by presenting performance gains relative to the baseline GBLUP model individually for each dataset. IFAM achieved average improvements in prediction accuracy of 9.46% (WTCCC1), 6.15% (UKB), 4.59% (Duroc pig), 4.09% (Yorkshire pig), and 10.83% (rice). The phrase "an average improvement of 6.86% across 20 traits" has been removed.*

3. The pseudo-annotation control design may be affected by confounders such as MAF and LD distance. The paper only states "random selection of the same category and quantity", but does not clarify whether these distributions were matched. Without such matching, the conclusion that real annotations outperform pseudo-annotations may be partly driven by distributional differences. It is recommended to add MAF/LD-matched controls to strengthen causal interpretability.

Response: *Thank you for this valuable suggestion. We agree that ensuring the comparability between pseudo annotations and real annotations is critical for the results. As recommended, we have performed a thorough analysis comparing the Minor Allele Frequency (MAF) and Linkage Disequilibrium (LD) distributions between the real and pseudo annotations. The results, presented in the Supplementary Fig.12 and Fig.13, demonstrated that the MAF and LD distributions are highly similar between the two sets of annotations. Thank you again for this comment, we believe that the supplied results have significantly strengthened the causal interpretability and rigor of our control design.*

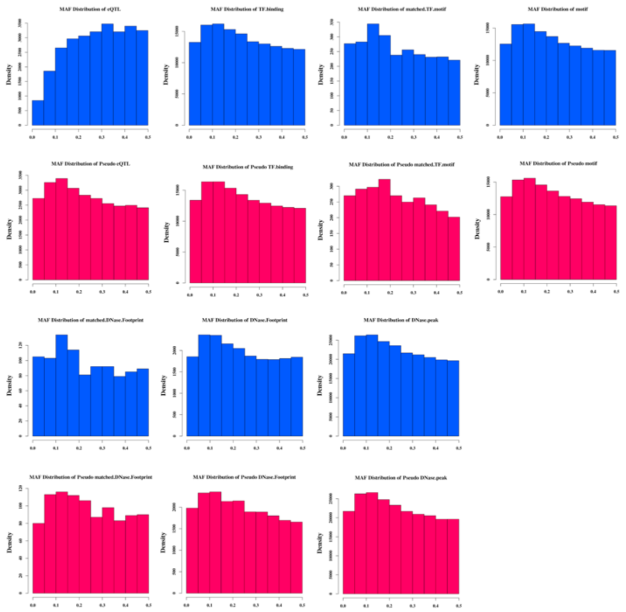

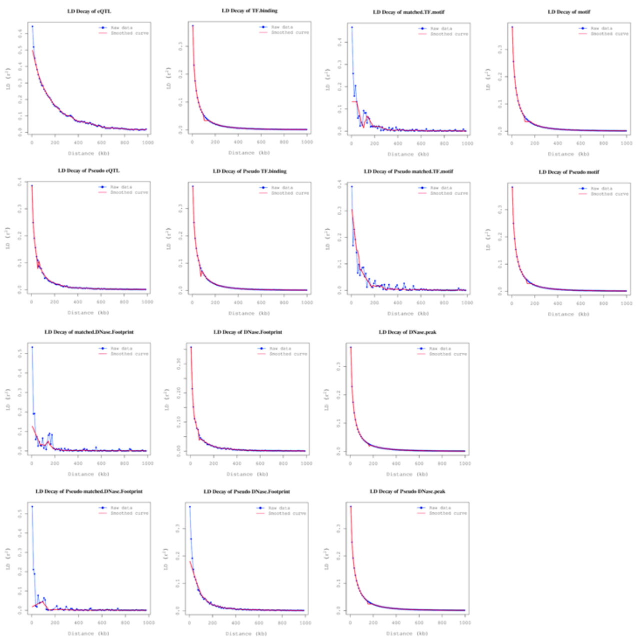

4. The baseline setup is unbalanced, which affects the fair assessment of the method's innovation. The WTCCC1 dataset was compared with BayesR, BayesRC, and MultiBLUP, but the UKB dataset lacks comparisons with mainstream methods such as SBayesRC, LDpred-funct, and AnnoPred.

Response: *Thank you for this insightful comment regarding baseline comparisons. In the UKB dataset, a more comprehensive benchmark was conducted including GBLUP, BayesR, non-auto-merged multiple random effects model, and IFAM (Supplementary Table 29~30). There are two types of models for incorporating the functional annotations in the genetic evaluation: one is the summary-level models such as SBayesRC, LDpred-funct, and AnnoPred; another is the individual-level models such as BayesRC and MultiBLUP. In this study, we focus on the individual-level model. Because these summary-level models rely on population-universal and chromosome-wide linkage disequilibrium (LD) matrices derived from common reference panels (e.g., the 1000 Genomes Project). However, such reference panels are impractical for plant or animal populations due to substantial genetic diversity across breeds or strains. Moreover, the LD structure within and among chromosomes in plants and animals is considerably more complex than that in humans. Ignoring inter-chromosomal LD and forcibly applying summary-level models to predict traits in these species would lead to suboptimal or unexpected performance. Briefly, the summary-level models (i.e., SBayesRC, LDpred-funct, AnnoPred) cannot be applied to non-human datasets (e.g., the pig and rice datasets used in our research). In order to keep consistency of the used models across different species, we only selected individual-level models to make comparisons with our proposed 'IFAM' model. In the revised manuscript, running Adaptive MultiBLUP and BayesRC was computationally infeasible at this scale (N>300,000). Therefore, GBLUP, BayesR, non-auto-merged multiple random effects model, and IFAM were run.*

5. The authors mention in the introduction and discussion the applicability of the IFAM method to plant and animal breeding. However, the current study only validates the method on human and animal datasets, with no plant data included. Given that plant genomic prediction is more complex (e.g., polyploidy, high heterozygosity, and genotype-environment interactions), it is recommended that the authors add validation on plant datasets.

Response: *Thank you for raising this important point. We agree that validation on plant datasets is crucial to fully support our claims regarding the broad applicability of the IFAM method. In response to this comment, we have included a new analysis using the rice dataset from the 3000 Rice Genome Project with annotations from the Rice Genome Annotation Project database to evaluate the performance of IFAM for predicting five yield-related traits. The results (Fig.6 and Supplementary Table 37~38), which are detailed in the revised manuscript, demonstrated that IFAM achieved an average improvement in prediction accuracy of 10.84% compared to the GBLUP model across these five traits. IFAM also consistently outperformed Adaptive MultiBLUP across all traits with a mean accuracy increase of 5.13%. Relative to BayesR, IFAM showed a slight average accuracy reduction of 1.54%; however, it demonstrated a superior prediction bias. And IFAM surpassed the non-auto-merged multiple random effects*

model by 4.88% in accuracy and delivered a more favorable bias. This new validation strengthens our conclusion that IFAM is a precise, efficient, and robust method that can also provide benefits in the context of plant breeding. We believe this addition significantly improves the manuscript and are grateful for the suggestion.

6. In the discussion, the authors mention comparing the IFAM model with the enhanced version of BayesRC, namely BayesRCO. However, the results do not present any findings related to BayesRCO. It is recommended that the authors provide further clarification, or explicitly include BayesRCO results in the Results section to ensure consistency.

Response: *Thank you for pointing out the issue. The term "BayesRCO" refers to the software we used to run the BayesRC model, which is adapted to cases where markers may belong to multiple annotations, please kindly see the Supplementary Note for further details. To eliminate any potential misunderstanding, we have revised the relevant sentence in the Discussion section of the revised manuscript to clarify this point.*

7. In the IFAM model, it is not clear whether performance necessarily improves as the number of annotation types increases. Since functional annotations are categorizations of SNP features rather than SNPs themselves, and SNPs are often assigned to multiple categories, such overlaps could affect variance component estimation and model stability. Substantial overlap among annotations might also increase the risk of overfitting. It is recommended that the authors address these points in the Methods or Discussion section.

Response: *Thank you for this valuable suggestion. We fully agree with these comments. The accurate functional annotations are key to improving genomic prediction accuracy. In the revised manuscript, we have included new analyses where IFAM was applied using functional annotations from both the RegulomeDB database and the LDSC model. The results indicated that the performance of IFAM using the annotations from LDSC was slightly lower than those from the RegulomeDB (Fig.2), probably due to the large number of annotations from LDSC and the high degree of overlap among them (Supplementary Fig.14), which would lead to suboptimal variance component estimates (Supplementary Table 15). Therefore, the performance of the IFAM model is not necessarily dependent on the quantity of annotations. To address the issue of overlap and ensure model stability, we consolidated random effects with similar variance magnitudes (10X) into a single term. This process can mitigate the impact of overlapping SNPs among annotations, simplify the model, and enhance the stability of variance component estimation.*

8. The authors report the computational efficiency of the IFAM model under different numbers of random effects, but do not compare it with that of other models. It is recommended to include such comparisons to more clearly demonstrate the advantages of the proposed method.

Response: *Thank you for this valuable suggestion. Following the recommendation, we conducted a computational efficiency comparison on the large-scale Yorkshire pig population under identical computing resources. The result (Supplementary Table 36) showed that GBLUP was the fastest (0.30 h), followed by IFAM (0.74 h) and Adaptive*

MultiBLUP (1.5 h). In contrast, BayesR required several days to complete. These results demonstrated that IFAM achieves the best predictive accuracy among the methods tested while maintaining a highly feasible computational time, effectively balancing prediction performance and efficiency.

Supplementary Table 36. *The computational efficiency of different models based on the Yorkshire pig dataset. All results were computed on a Linux server with 32 CPUs and 960 GB of memory, and the computational times (hour, h) were recorded. For the Adaptive MultiBLUP, the procedure was killed during the variance component estimation for D100, BF100, and LMA100 traits.*

Trait[1]	***IFAM***	***GBLUP***	***Adaptive MultiBLUP***	***BayesR***
D100	*0.90 h*	*0.35 h*	*1.67 h*	*>24 h*
BF100	*0.90 h*	*0.36 h*	*1.79 h*	*>24 h*
LMA100	*0.77 h*	*0.31 h*	*1.68 h*	*>24 h*
LTN	*0.61 h*	*0.25 h*	*2.16 h*	*>24 h*
RTN	*0.65 h*	*0.27 h*	*1.60 h*	*>24 h*
TTN	*0.58 h*	*0.24 h*	*1.60 h*	*>24 h*

[1] *D100: age adjusted 100 kg; BF100: backfat thickness adjusted to 100 kg; LMA100: loin muscle area adjusted to 100 kg; LTN: left teat number; RTN: right teat number; TTN: total teat number*

9. The LDAK software is mentioned without providing the relevant reference; it is recommended that the authors add the appropriate citation.

Response: *Thank you for pointing out this omission. The appropriate citation for the LDAK software has now been added to the revised manuscript.*

10. In Figure 6, the legend label "IFAM_KMAL" should be corrected to "IFAM_KAML".

Response: *Thanks for your meticulous attention to detail. In the revised manuscript, the results corresponding to the label "IFAM_KMAL" have been removed. Therefore, this specific label is no longer present in the current version of Fig.6.*

11. The sentence "All authors reviewed and approval the final manuscript" should be revised to "approved".

Response: *Thank you for pointing out the issue. We have modified this grammatical error in the revised manuscript.*

Responses to Reviewer #2:

Review report

The manuscript proposes a model (IFAM) to integrating functional annotations for genomic prediction. The IFAM is a typical linear mixed model with multiple genetic components. Instead of treating each annotation type as one genetic component, IFAM merges the annotation types with similar variance of SNP effect to be one genetic component in the model. The model together with regular GBLUP and some other models were used for genomic prediction by integrating functional annotation information. The results showed that functional annotation information can improve accuracy of genomic prediction. Merging annotation types with similar variance to be a genetic component in the model is a good strategy. In addition, the results from the analysis on the contributions of different annotation categories to genetic variation of complex traits indicate the importance of functional annotation information. As the whole, it is a good study and provides valuable method and information for the improvement of genomic prediction.

The functional genomic annotation information used in this study were obtained from the website of the LD Scores Regression (LDSC) model, RegulomeDB database, and IFmut database (pig), and the data in the analysis were obtained from Welcome Trust Case-Control Consortium (WTCCC), UK Biobank, and a pig dataset (source not provided). These data appear suitable for this study. It will be helpful to describe how to choose the six traits from UK Biobank and the seven traits from the pig dataset in this study.

Various models are used for genomic prediction based on the above data sets. In general, the analysis methods are reasonable, but the descriptions of some scenarios need to be more detailed. See detailed comments below.

The results are relevant. However, the scenarios/models/datasets on which the results are based are not clearly described, so that it is a bit difficult to distinguish the approaches and compare the results. See detailed comments below.

The discussion connects well with the results. The conclusions are well supported by the results. However, the discussion is somewhat superficial and needs to be strengthened. For example, it would be helpful to discuss the strategy for merging functional annotation types (which may be different in different situations), the results that BayesRC which use annotation prior performs worse than BayesR which do not use annotation prior, and so on.

The following are the detailed comments. Due to the lack of line numbers in the manuscript, it may not be clear where the comments correspond to the text.

Response: *We sincerely thank you for your positive assessment and professional suggestions. We have revised the manuscript according to your professional suggestions.*

Introduction

Page 2: “Genomic prediction, also known as genomic selection”. It is not really correct. Genomic selection relies on genomic prediction but includes other relevant things within breeding programs.

Response: *Thank you for pointing out the issue. We agreed that "genomic selection" encompasses a broader breeding program context beyond the statistical prediction itself. We have therefore removed the phrase "also known as genomic selection" from the revised manuscript. The text now correctly refers to the method as "genomic prediction."*

Page 2: “challenges persist …”. Dependency on LD is not the only challenge. Even when using sequencing data that includes all variants in the genome, challenges remain. The same comment on “bypass the LD issues” in page 3. It is also not clear to what the authors refer with “functional annotations”. Also, “Therefore” does not connect the two sentences, since one still does not know which traits the QTNs affect, even with functional annotations.

Response: *Thank you for these insightful comments. We have refined the relevant wording as suggested to ensure clarity and rigor.*

Genomic prediction plays an important role in plant and animal breeding, as well as in human precision medicine. The genomic prediction relies on linkage disequilibrium (LD) between single-nucleotide polymorphisms (SNPs) and their nearby quantitative trait nucleotides (QTNs, also known as causal variants) to predict objective traits. A variety of genomic prediction models have been proposed, including methods under the Best Linear Unbiased Prediction (BLUP) framework, e.g., genomic BLUP (GBLUP), Single-step genomic BLUP, and MultiBLUP; and the family of methods constituting the “Bayesian alphabet”, such as BayesA, BayesCπ, and Bayes. In particular, the MultiBLUP and BayesR are representative methods among them, the former allowed a mixture of normal distribution of SNP effects by extending the BLUP to include multiple random effects, while the latter assumed four distinct classes (zero, small, medium, and large) for SNP effects. The flexible assumptions on the distribution of SNP effects allow them to capture the genetic architecture of complex traits, making it more accurate than other methods.

Studies have revealed that the genetic map explains approximately 5%-49% of the heritability of complex traits, and the “missing heritability” is widespread in genetic evaluation. The whole-genome epigenetic map and other omics data, as a bridge between the genetic map and the phenotype, could potentially solve it to a certain extent. Incorporating omics data of related processes such as gene transcription and translation into the prediction model not only enhances the dissection of complex traits, thereby improving the accuracy of prediction, but also endows the results with stronger biological relevance. The functional annotations of the genome refer to the process of mapping genomic variants to their biological roles, and their data is more readily understood, obtained, and utilized. Therefore, integrating the functional annotations into genomic prediction models can offer a promising strategy for improving prediction accuracy and conducting omics-based genetic evaluation and breeding.

Page 4. “the groups of genetic markers with similar contributions to phenotypic variance were automatically merged into one single random effect during variance assessment”. Grouping single genetic markers or grouping functional annotation types?

Response: *Thank you for pointing out this ambiguity. The grouping and merging process applies to the functional annotation types, not to single genetic markers. We have revised the relevant sentence in the manuscript to explicitly state "the functional annotation types with similar contributions to phenotypic variance were automatically merged into one single random effect" to ensure clarity and precision.*

Compared statistical methods can be mentioned here. Some methods are listed in the introduction but they are not the ones used for comparison. What are their drawbacks, how IFAM differs from them, and what it solves in practice. Why we need IFAM?

Response: *Thank you for this valuable suggestion. We have revised the Introduction section to include a more focused comparison of statistical methods. The revised manuscript now explicitly discusses the specific methods used for comparison in our study, outlines their limitations, and more clearly articulates how IFAM differs from them and the practical problems it aims to solve. This provides a stronger rationale for the development and application of our method.*

Methods and data

Page 21: There are a large number of traits with phenotypic measurements in the UK Biobank, and there may be also a lot of traits in the pig dataset. How you choose some of these traits in this study?

Response: *Thank you for pointing out the issue. The six representative traits from the UK Biobank were selected based on several considerations: diversity in genetic architectures (ranging from less to highly polygenic) as inferred from GWAS results, computational feasibility, and comparability with prior studies, such as Lloyd-Jones et al. Regarding the Duroc pig dataset from Yang et al., all seven publicly available traits were included in our analysis. We have now clarified these selection criteria in the revised manuscript.*

Page 21, WTCCC1 dataset: “a p-value < 0.05 from the Hardy-Weinberg equilibrium test”. Why p<0.05 instead of 10^-6 as used in UK Biobank dataset? Furthermore, the control:case ratio in the dataset was approximately 3:2, which is not a random sample (the ratio of disease is much higher than the true incidence), so the Hardy-Weinberg equilibrium test is invalid. How many SNPs were left out of 450K?

Response: *Thank you for pointing out the issue. The different HWE p-value thresholds for the WTCCC1 (p < 0.05) and UK Biobank (p < 1e-6) datasets were adopted from the published studies we used as references—specifically, MultiBLUP (Speed et al.) and BayesR (Moser et al.) for WTCCC1, and SBayesR (Lloyd-Jones et al.) for UKB. We have clarified this rationale in the revised manuscript. We acknowledged that the case-control imbalance in the WTCCC1 dataset can affect the interpretation of the HWE test. This filter was applied primarily to maintain consistency and comparability with previous studies. Following the quality control, approximately 370K SNPs were*

retained for the WTCCC1 dataset (Supplementary Table 1).

Page 22, "unrelated individuals of European ancestry was selected….". Unrelated how?

Response: *Thank you for requesting this clarification. The term "unrelated" was indeed insufficiently defined. We have revised the manuscript to specify the exact criterion used: a subset of 348,501 unrelated individuals of Europe was selected, where "unrelated" is defined as having an absolute value of the off-diagonal in the Genomic Relationship Matrix (GRM) of less than 0.05 referring to the study of Lloyd-Jones et al. This description has been added to the revised manuscript.*

Page 22, UK Biobank dataset: "filtered based on …". Use either the removed criteria or the retained criteria, but not a mixture.

Response: *Thank you for pointing out the issue. We have revised the relevant description in the manuscript to clearly present the criteria used for the quality control in the UK Biobank dataset: "the SNPs with MAF > 0.01, probability of departure from Hardy-Weinberg equilibrium < 10^{-6}, and missingness < 0.05 were retained, which resulted in 1,094,840 SNPs".*

Page 22, Pig dataset: "low-coverage". How low? Explain why quality control criteria for MAF and call rate are different from those used in the WTCCC1 dataset and UK Biobank dataset.

Response: *Thank you for pointing out these important questions regarding the pig dataset. The pig dataset downloaded from the study of Yang et al., which proposed a Tn5-based highly accurate, cost- and time-efficient, low-coverage sequencing technology to obtain 11.3 million whole-genome SNPs in the Duroc pig population at a mean depth of 0.73×. The initial, more stringent MAF threshold for the pig dataset was chosen due to the smaller population size. Moreover, to ensure method consistency across all datasets in our study, we have re-analyzed the pig dataset using the same MAF criteria applied to the human datasets. The relevant description and results have been incorporated into the revised manuscript.*

Page 24, multiple random effect model, and elsewhere: "Mu is a vector of genetic effects". Using other letter (e.g., u) to represent random effect, because mu usually represents overall mean or intercept in a model.

Response: *Thank you for pointing out the issue. We agreed that using "μ" for a random effect is non-standard, as it is conventionally reserved for the overall mean. We corrected this mistake in the revised manuscript and used a unified symbol to represent the fixed effects and random effects throughout the text.*

Page 24: "additive marker covariates matrix". What does "additive" mean here?

Response: *Thank you for pointing out the issue. The term "additive" was used to specify the marker covariates matrix for constructing an additive genetic relationship matrix (GRM). Since our study does not involve dominance effects, we agreed that this specification is redundant and potentially confusing. We have therefore removed the word "additive" from the revised manuscript.*

Page 24: "sigma_gk^2 is the estimated genetic variance of $\boldsymbol{Gk}$". It should be "the genetic variance accounted for by SNPs in the kth annotation set.

Response: *Thank you for pointing out the issue. The original description was imprecise, and we have revised it in the manuscript.*

Page 25: "with approximate variance component (within 10X)". 1) Need to point out 'similar variance per-SNP'. 2) Explain why it is 10x, is it optimal?

Response: *Thank you for pointing out these critical questions. The merging was performed based on the total variance component of each annotation type, not the per-SNP explained variance. We have clarified this point in the revised manuscript.*

Rationale for the 10X threshold: *The choice of a 10X difference as the merging criterion was guided by two primary considerations. First, it effectively groups annotations with a similar order-of-magnitude contribution to the total genetic variance, thereby mitigating the impact of overlapping SNPs among annotations, simplifying the model, and enhancing the stability of variance component estimation. Second, this threshold aligns with the framework of Bayesian methods like BayesR, which categorize effect sizes (e.g., explaining 0.01%, 0.1%, and 1% of the total genetic variance) into distinct, order-of-magnitude separated classes. We found the 10X rule to be a robust and generally applicable heuristic for capturing the genetic architecture across diverse complex traits.*

Page 25: "potentially addressing the convergence problem". The automated step of merging random components should be detailed. How do you estimate variance for each annotation type before merging them? How to avoid convergence issue, if a multiple random genetic effect model that includes all annotation types is used to estimate the variance of each annotation type simultaneously? The same comment on Page 26, "…iterative convergence…". The equation is not self-explanatory.

Response: *Thank you for these detailed questions, which have allowed us to significantly improve the method description in the manuscript. The automated merging process of variance components and its rationale have been explained in detail in the revised Methods section. Briefly, the variance components for all annotation types were estimated simultaneously within a multiple random effects model using the AI-REML algorithm (or the HE regression method for large datasets).*

We acknowledged that while this estimation generally converges, instability and anomalous estimates can occur with a very high number of annotations (e.g., 74 annotations from the LDSC model), likely due to the estimates falling into a local optimal solution. However, in the IFAM model, the variance components estimated in the first round of iteration are not directly used to derive individual genetic effects. Instead, they serve as prior information for the downstream merging of multiple annotation types. This merging process reduces the number of random effects in the model, thereby facilitating more reliable and stable estimations of both variance components and individual genetic effects in the second round of iteration.

Page 25: "We first ordered the variances of multiple random effects". Variance of the annotation type or variance per-SNP in the annotation type? Similar to above.

Response: *Thank you for seeking this clarification. The merging procedure is based on the total variance component of each annotation type, not the per-SNP explained variance. This point has been explicitly stated in the revised manuscript to prevent any potential misunderstanding.*

Page 26, Evaluating functional annotations: "we used the variance ……contribution of functional annotations". 1) By definition, variance of SNP effect is sigma_a^2/sum of 2pq. 2) Are the SNPs used in estimating variance components and genomic predictions also pruned? Should they be kept consistent?

Response: *Thank you for these insightful questions regarding the method consistency.*
1. Variance Calculation: We agreed on the definition of SNP effect variance. Sorry for the incorrect descriptions, we used the average phenotype variance explained (PVE) by per-SNP instead of the variance of SNP effect. We have revised it in the manuscript.

2. Consistency of SNP Sets: The SNP sets differed: pruning was only applied to evaluate the annotation importance but was not used in the IFAM prediction stage.

When evaluating annotation importance, the SNPs within a strong LD block should contribute the same PVE, regardless of the number of SNPs contained in the block. Without LD pruning, the importance of such blocks would be underestimated. Therefore, we divided the total genetic variance attributed to each annotation by the number of pruned SNPs, which we believe provides a fairer and more accurate evaluation across different annotations. In contrast, refer to our previous study, the marker density has only a light impact on prediction accuracy. Therefore, SNPs in strong LD do not affect the final prediction accuracy during the prediction stage. Consequently, we retained the original genotype data for genomic prediction.

Page 26: "To conduct this analysis, we used a multiple random effects GBLUP model". all annotation types simultaneously? Maximum number of components? Problem with convergence?

Response: *Thank you for pointing out this pertinent question. Yes, the importance of all annotation types was evaluated simultaneously. In this study, the maximum number of components was 7, corresponding to 7 categorical rankings from the RegulomeDB database. Model convergence was achieved reliably for analyses with a moderate number of annotations, such as the 7 from RegulomeDB. However, we observed that with a large number of annotations (e.g., the 74 types from the LDSC model), the estimation of variance components could become unstable and produce anomalous estimates. Therefore, we have included a recommendation in the revised manuscript to caution against using an excessively high number of annotations to ensure robust estimation.*

Page 27: "ranking scores of SNPs from the RegulomeDB database". But RegulomeDB database has already grouped SNPs by ranking score.

Response: *Thank you for pointing out the issue. Indeed, the RegulomeDB database provides pre-defined rankings of variants (Rank 1-7). Our objective was not to re-rank these categories, but to use our proposed annotation importance evaluation strategy to independently estimate the genetic importance and phenotypic impact of SNPs within*

each pre-defined category. A strong concordance between our estimated importance and the established RegulomeDB ranking would validate the effectiveness of our strategy. We have clarified this objective in the revised manuscript.

Page 27, Cross-validation and prediction accuracy: It is mentioned that For UKB dataset, GWAS were carried out using Linear regression model. For WTCCC and pig data, are the GWAS results used in genomic prediction from GLM model or MLM model?

Response: *Thank you for seeking this clarification. The GWAS results used for genomic prediction were generated as follows: a generalized linear model (GLM) was used for the WTCCC1 dataset, and a mixed linear model (MLM) was used for the pig dataset. We have explicitly stated this information in the revised Methods section.*

Page 27: "The predictive ability using Pearson's correlation". How about the bias of prediction?

Response: *Thank you for this valuable suggestion. In response, we have included the calculation of prediction bias in our analysis. The corresponding results have been provided in the Supplementary Materials (Supplementary Tables 26, 30, 32, 35, 38), and a discussion of their implications has been added to the revised manuscript.*

Results

Results may be restructured. The data sets were already discussed partially in the first two subsections (Annotated results of three datasets, The importance of functional annotations), but then there are separate sections for each.

Response: *Thank you for this constructive feedback on the results structure. We agree that the previous organization led to some redundancy. In the revised manuscript, we have completely restructured the Results section to present a clearer and more logical flow. The findings for each dataset are now presented in dedicated, consolidated subsections, eliminating the prior overlap, and ensuring a more streamlined and focused presentation of the experimental outcomes.*

Page 5: "we found that the top five annotation covering …". Need to mention that the results are based on WTCCC data. Have you inspected the distribution of the LDSC annotation types in UKB data?

Response: *Thank you for this suggestion. We have clarified in the manuscript that the initial analysis of annotations was based on the WTCCC1 data. Furthermore, as recommended, we have included a parallel analysis inspecting the distribution of the LDSC annotation types in the UKB dataset (Supplementary Table 11).*

Page 6, Fig 1:"model by incorporating trait specific multiple random effects". Include also random effects of annotation types from the annotation datasets?

Response: *Thank you for pointing out the issue. Yes, the model incorporates multiple random effects derived from both the 7 annotation types in the RegulomeDB database and the trait-specific significant association signals. We have clarified this point in the*

revised manuscript to prevent any potential misunderstanding.

Page 6, Fig 1: A discussion or explanation on the big difference in these proportions between WTCCC1 and UKB datasets.

Response: *Thank you for highlighting this discrepancy. The difference in proportions between the WTCCC1 and UKB datasets in the original figure was primarily due to the use of inconsistent color schemes in the legend. We have revised Fig.1 (now Supplementary Fig.1) to use a consistent color scheme, which confirms that the proportions are similar between the two datasets.*

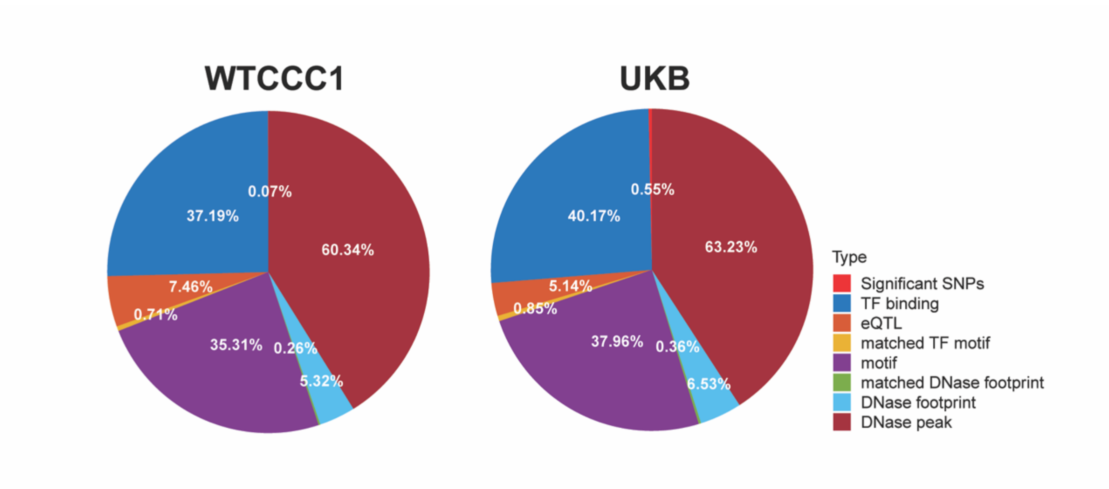

Page 7: “we accessed the difference”. “accessed” should be “assessed”. The mistake appears many places throughout the manuscript.

Response: *Thank you for pointing out the issue. The word "accessed" has been corrected to "assessed" throughout the manuscript.*

Page 7: “heritability of each category decreased gradually”. This is a trend, not for all points. For example, Rank 4 has a larger variance than Rank 3 in 5 of 6 UKB traits.

Response: *Thank you for this precise observation. We have revised the manuscript to describe the trend more accurately, stating that the heritability estimates showed an "overall decreasing trend" across annotations about ranking scores, rather than implying a strict monotonic decrease for every single data point.*

Page 7: “Besides, the findings also indicated that eQTL information ……such as DNase footprints and motifs”. Mention which table or figure shows these results.

Response: *Thank you for pointing out the issue. An assessment of the importance of annotation types from the RegulomeDB database has been added. The results, presented in the Fig.1 (right), indicated that the matched TF motif, eQTL, and matched DNase footprint annotations encompass the most crucial functional information.*

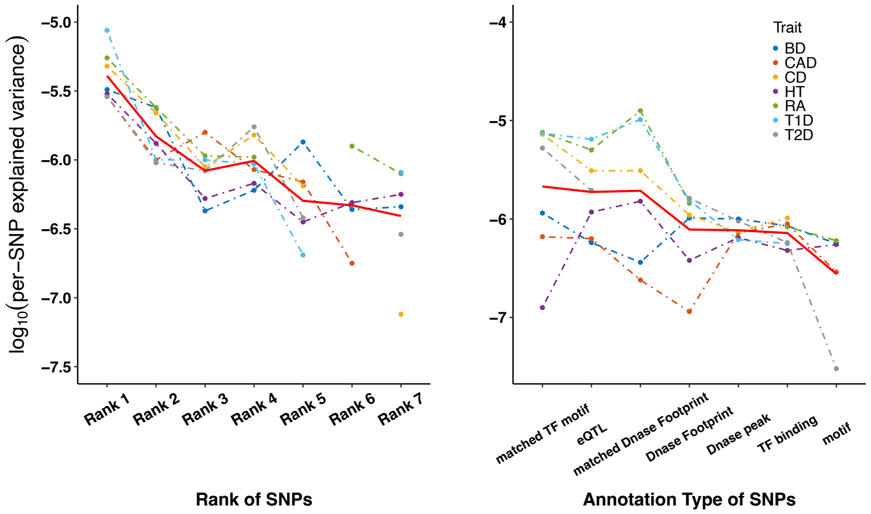

Page 8: “different ranking scores in the WTCCC1 dataset “. Did you also do the assessment in UKB dataset?

Response: *Thank you for pointing out the issue. The assessment of genomic prediction accuracy across different ranking scores from the RegulomeDB database, initially conducted on the WTCCC1 dataset, was found to be less central to the main conclusions of our study. In order to streamline the manuscript and focus on the core results, we have removed this analysis from the revised manuscript. Consequently, the UKB dataset was not used for this evaluation.*

Page 8: “The higher the ranking score, the lower was …”. This is a trend, not for all points. For example, e.g., Rank 4 is higher than Rank 3, Rank 5 lower than rank 6 and 7.

Response: *Thank you for this precise observation. We agree that the trend described was not strictly monotonic across all points. Upon re-evaluation, the genomic prediction accuracy comparison of ranking scores is not central to our conclusions. Therefore, we have removed this result from the revised manuscript to maintain focus on the key findings.*

Page 9: “nearly 100 types. Present in detail, is LDSC 74 + RDB 7 + GWAS 1?

Response: *Thank you for pointing out this ambiguity. The phrase "nearly 100 types" was an approximation and potentially misleading, and we did not simultaneously use annotations from both RegulomeDB and LDSC. We have removed this phrase from the revised manuscript to ensure precision and avoid any potential confusion.*

Page 9: “GRMs based on the position information…”. How did you use the position information to construct GRMS?

Response: *Thank you for seeking this clarification. The original description was imprecise. We have revised the manuscript to state clearly that GRMs were constructed using SNPs located within the genomic region defined by the positional information of annotations.*

Page 9: “the GBLUP model that contained multiple random effects”. Did you estimate variance components for each annotation component simultaneously using a model containing about 100 random genetic components/GRMs? No problem with convergence?

Response: *Thank you for pointing out this issue. Yes, the variance components for all annotation types were estimated simultaneously within a multiple random effects model using the AI-REML algorithm (or the HE regression method for large datasets). We acknowledged that while this estimation generally converges, instability and anomalous estimates can occur with a very high number of annotations (e.g., 74 annotations from the LDSC model), likely due to the estimates falling into a local optimal solution. However, in the IFAM model, the variance components estimated in the first round of iteration are not directly used to derive individual genetic effects. Instead, they serve as prior information for the downstream merging of multiple annotation types. This merging process reduces the number of random effects in the model, thereby facilitating more reliable and stable estimations of both variance components and individual genetic effects in the second round of iteration.*

Page 9: Finally, how many genetic components (or GRMs) are in IFAM model? Are the classes in BayesRC the same as the genetic components in IFAM?

Response: *Thank you for pointing out this issue. We have reported the final number of genetic components in the IFAM model across all traits in the Supplementary Fig.17. The number typically ranged from 2 to 3, and was consistently within 5 components. Furthermore, we confirmed that BayesRC and IFAM were applied to the same functional annotations for a comparison, a point we have explicitly stated in the revised manuscript.*

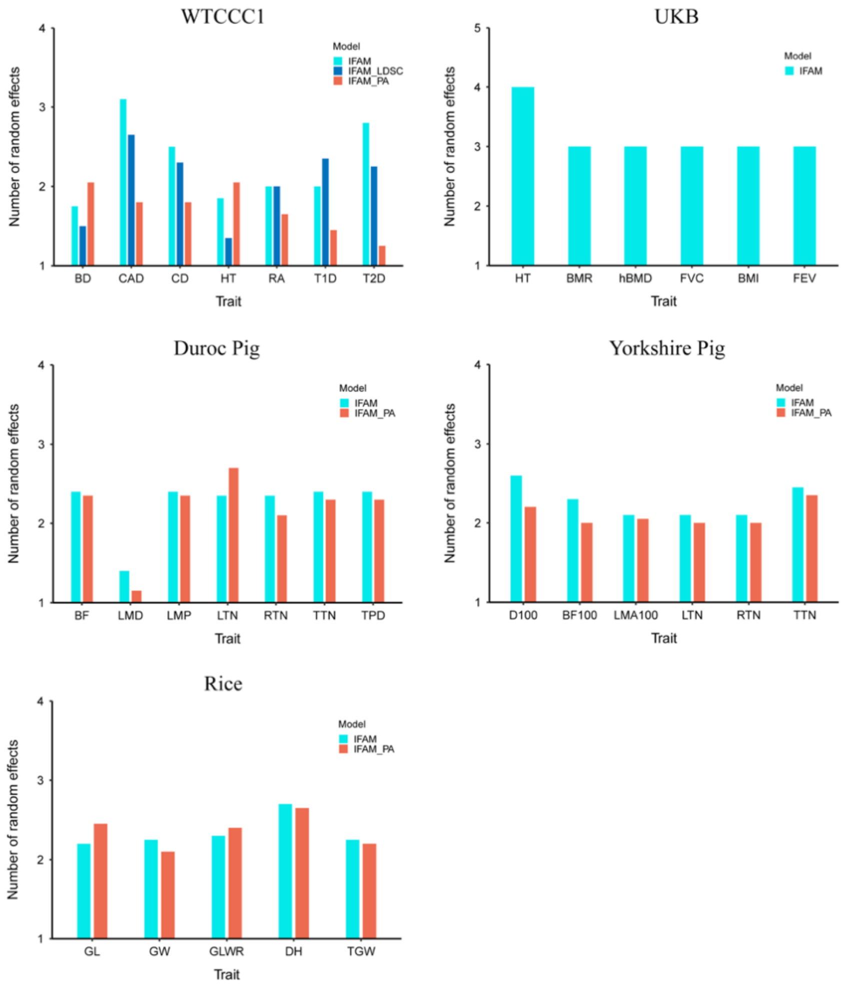

Page 10, Fig. 3: "with two sets of functional annotations". Include also GWAS information?

Response: *Thank you for pointing out this issue. Yes, the functional annotations included the trait-specific significant SNPs identified from GWAS. We have clarified this point in the revised manuscript to prevent any potential confusion.*

Page 10, Fig. 3: BayesRC use both LDSC and RegulomeDB annotations or one of the two? What are the possible reasons that BayesRC which uses annotation prior performs worse than BayesR which does not use annotation prior.

Response: *Thank you for these insightful questions.* ***Annotations for BayesRC****: In this analysis, BayesRC utilized only the annotations from the RegulomeDB database.*

***Performance of BayesRC vs. BayesR**: We attributed this primarily to annotation complexity and potential non-convergence. Notably, original and subsequent applications of BayesRC typically used only 2~3 pre-processed annotations, whereas our analysis employed raw, overlapping annotations without filtering. When we restricted the BayesRC to two annotations (eQTL and matched TF motif) based on WTCCC1 data, its accuracy improved markedly, rivaling GBLUP, particularly for T1D (Supplementary Table 28). Furthermore, while increasing MCMC iterations to 100,000 yielded minor improvements, the gains were insignificant. Given its computational intensity and lack of parallelization, further increasing iterations was impractical. These results suggested that BayesRC requires high-quality, trait-relevant annotations to perform optimally. Since previous studies indicated that BayesRC generally offers marginal gains over BayesR, a baseline method included in our comparisons, this limitation does not undermine the comparative assessment of IFAM against Bayesian approaches.*

***Supplementary Table 28.** Prediction performance of the different scenarios of BayesRC in the WTCCC1 dataset using the functional annotation from the RegulomeDB database. The "BayesRC" represents running the BayesRC using the default parameters, where the MCMC chain length (Numit) was set to 50,000, the burnin steps were set to 20,000, and the functional annotations are the same as the IFAM. The "BayesRC_opt1" represents running the BayesRC using the alternative parameters, where the MCMC chain length (Numit) was set to 100,000, the burnin steps were set to 40,000, and the functional annotations are the same as the IFAM. The "BayesRC_opt2" represents running the BayesRC using the alternative parameters, where the MCMC chain length (Numit) was set to 50,000, the burnin steps were set to 20,000, and the functional annotations are eQTL and matched TF motif.*

Trait[1]	BayesRC Numit=50K	BayesRC_opt1 Numit=100K	BayesRC_opt2 Two annotations
BD	0.5261(0.0043)	0.5261(0.0041)	0.5583(0.0042) **
CAD	0.5178(0.0042)	0.5189(0.0036)	0.5378(0.0048) **
CD	0.5371(0.0047)	0.5384(0.0046)	0.5821(0.0043) **
HT	0.5160(0.0031)	0.5166(0.0033)	0.5438(0.0037) **
RA	0.5291(0.0041)	0.5301(0.0040)	0.6581(0.0036) **
T1D	0.5510(0.0043)	0.5536(0.0044) *	0.7828(0.0024) **
T2D	0.5256(0.0036)	0.5247(0.0037)	0.5582(0.0037) **
Mean	0.5290	0.5298	0.6030

[1] BD: bipolar disorder; CAD: coronary artery disease; CD: Crohn's disease; HT: hypertension; RA: rheumatoid arthritis; T1D: type 1 diabetes; T2D: type 2 diabetes;
** represents a significant difference (P < 0.05) in the prediction accuracy between the targeted scenario and BayesRC;*
*** represents an extremely significant difference (P < 0.01) in the prediction accuracy between the targeted scenario and BayesRC*

Page 10, Fig.3: "20-fold cross-validation". It is not 20-fold cross validation. For 20-fold cross validation, the whole data should be divided into 20 subset, a subset is used as a validation set. This comment also applies elsewhere related to 20-fold cross validation.

Response: *Thank you for pointing out the issue. The initial description of the cross-validation strategy was indeed confusing. All the model evaluations and comparisons in our manuscript were conducted using a random splitting strategy, thus we have revised the manuscript to consistently refer to the approach as "repetitions" rather than "k-fold cross-validation" throughout the Results section and figure captions.*

Page 10, Fig. 3: It is helpful to describe how to apply annotation information in the adaptive multiblup model. Did you have the results from a regular multi-genetic component GBLUP model (e.g., GBLUP model with 7 annotation types from RegulomeDB as 7 genetic components)?

Response: *Thank you for pointing out the issue. The Adaptive MultiBLUP model did not utilize functional annotations. This model used local genomic relationship matrices to automatically identify and weight genomic regions that were significantly associated with the trait. A detailed description of the Adaptive MultiBLUP was provided in the Supplementary Note. As suggested, the results of a regular multi-genetic component GBLUP model, where each of the annotation types was viewed as a single random effect, have been added to the revised manuscript (Supplementary Tables 25, 29, 31, 34, 37).*

Page 10: "the IFAM model using another set … from the RegulomeDB database". Clarify what set is it?

Response: *Thank you for requesting this clarification. The "another set" of annotations refers to those from the LDSC model. We have explicitly stated this point in the revised manuscript to prevent any potential confusion.*

Page 10: "highly consistent (Fig.3, Supplementary Table 14)". No results from another set can be seen in Fig.3 and Supplementary Table 14. This paragraph is confusing with the next one.

Response: *Thank you for pointing out this lack of clarity. There are two sets of functional annotations: one is from the RegulomeDB database, and another is from the LDSC model. We have revised the caption of Fig.3 (now Fig.2) and the title of Supplementary Table 14 (now Supplementary Table 25) to explicitly state that the results for "IFAM" and "IFAM_LDSC" correspond to the IFAM model using the two distinct annotation sets, which are the RegulomeDB database and mainly from the LDSC model, respectively.*

Page 11: "conventional annotation information was slightly better than …". Indicate the table/figure which show the difference in prediction accuracy between the two approaches.

Response: *Thank you for pointing out the issue. The original description regarding this result was indeed confusing. Here, the comparison was between two different forms of annotation from the RegulomeDB database: annotation types and annotation ranking. To streamline the results and focus on the key findings, we have removed the results of IFAM based on annotation ranking from the revised manuscript. The results for the annotation types, which are the primary focus, are clearly presented in Fig.2.*

Page 13, Fig. 4a: What is the base/reference for calculating the improvement in accuracy of IFAM_Rank and IFAM_KAML?

Response: *Thank you for pointing out the issue. The "IFAM_Rank" refers to running IFAM using the ranking scores from the RegulomeDB database as annotation information. The "IFAM_KAML" involved optimizing the construction of the genetic relationship matrix in the IFAM model using the strategy from the KAML model. The base/reference is the prediction accuracy of IFAM using the annotation types from the RegulomeDB database. However, to streamline the results and enhance clarity, we have removed the results for "IFAM_Rank" and "IFAM_KAML" from the revised manuscript.*

Page 13: "functional annotation information from the RegulomeDB database". It would be helpful to explain why some prediction model used both LDSC and RegulomeDB annotations, and the other used only RegulomeDB annotation.

Response: *Thank you for requesting this clarification. We have revised the manuscript to explicitly state the annotation information used for each model and the rationale behind these choices. On the WTCCC1 dataset, IFAM was evaluated separately using annotations from the RegulomeDB database and the LDSC model to assess the impact of annotation quantity on the performance of IFAM. For the UKB dataset, due to its substantial computational demands, the analysis was conducted using only the RegulomeDB annotations to ensure feasibility.*

Page 9-14: It is not clear what models used GWAS information.

Response: *Thank you for seeking this clarification. In this study, in addition to species-specific functional annotation information, the SNPs associated with interesting traits were viewed as a type of trait-specific annotation. So, the models described as utilizing functional annotations all incorporated the trait-specific significant SNPs identified from GWAS as a part of the annotations. We have explicitly stated this in both the Methods and Results sections of the revised manuscript to ensure clarity.*

Page 14: "using 10-fold cross-validation". The same comment as on 20-fold cross-validation above.

Response: *Thank you for pointing out the issue. We have revised the manuscript to consistently refer to the approach as "repetitions" rather than "k-fold cross-validation".*

Page 15: After merging annotation types, how many genetic components (GRMs) in the three IFAM models?

Response: *Thank you for pointing out the issue. We have explicitly reported the final number of genetic components (random effects/GRMs) for all IFAM models in the revised Supplementary Fig.17. The number ranged from 2 to 3 components in most cases and was always within 5 components.*

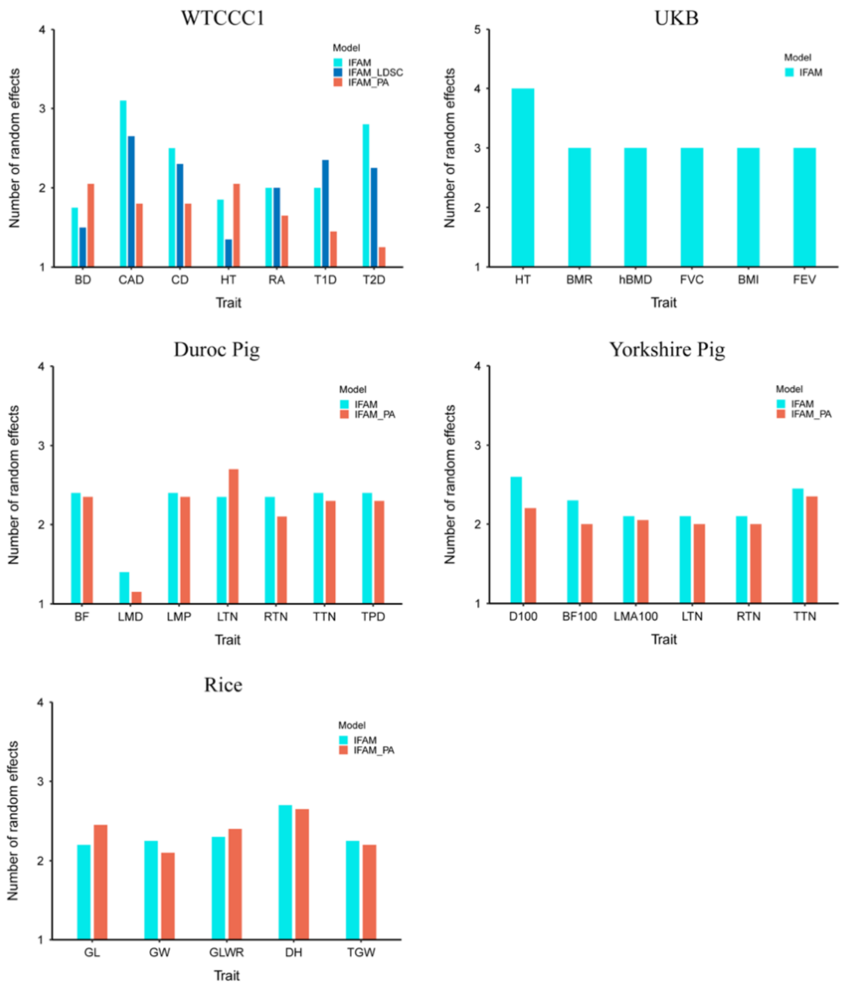

Page 15, Fig.6: "which included 10 types of annotations". Include also GWAS information?

Response: *Thank you for pointing out the issue. Yes, the annotation information incorporated the trait-specific significant SNPs identified from GWAS. We have clarified this point in the revised manuscript.*

Page 9-15: For regulomeDB (maybe also IFmut), there are only 7 types of annotations, a regular multiple genetic component model may perform well. It may be worth evaluating.

Response: *Thank you for this valuable suggestion. As recommended, we have included an evaluation of a regular multiple genetic component model using the same annotations. The results (Supplementary Table 25, 29, 31, 34, 37), detailed in the*

revised Results section, demonstrated that IFAM consistently outperformed the regular multiple genetic component model across all five datasets. Furthermore, the performance of the regular model degraded as the number of annotations increased, highlighting the specific advantage of the data-adaptive merging procedure of IFAM.

Discussion

In this study, some prediction models used both LDSC and RegulomeDB annotations, and the other used only RegulomeDB annotation. It is expected that IFAM may have larger advantage when using LDSC annotation information than using RegulomeDB annotations, because there are 74 annotation types in LDSC and only 7 on RegulomeDB, the effect of merging annotation types would be more meaningful for LDSC annotations. It is worth discussing under what circumstances IFAM can lead to more improvements in genome prediction compared with other models. In addition, it is helpful to discuss the issue that BayesRC which use annotation prior performs worse than BayesR which do not use annotation prior.

Response: *Thank you for these insightful comments, which have helped us significantly strengthen the discussion in the manuscript. We have revised the Discussion section to explicitly address the points raised. Firstly, we clarify that IFAM's performance improvement is not a simple function of annotation quantity but is more strongly dependent on the relevance of the annotations to the interesting trait. However, as the number of annotations increases, the merging strategy within IFAM becomes crucial for maintaining stability and preventing the performance degradation seen in the non-auto-merged multi-component models, thereby providing a more significant advantage with larger annotation sets like LDSC.*

Secondly, we have expanded the discussion on the unexpected underperformance of BayesRC. We elaborated on potential reasons, primarily centered on its sensitivity to the quality and complexity of annotations, and potential non-convergence. This contrast indeed underscores the necessity of developing stable and efficient methods, like IFAM, for leveraging functional annotations in genomic prediction.

Page 17: “the BayesRCO (enhanced version of BayesRC)……”. In the RESULT section, there are results from BayesRC, not BayesRCO.

Response: *Thank you for pointing out the issue. The mention of "BayesRCO" could cause confusion. Due to the BayesRC is not adapted to cases where markers may have multiple annotations, we chose the BayesRC+ approach, which was an enhanced version of BayesRC through a cumulative way to account for the contribution of multiple annotation categories and was implemented in the BayesRCO software. We have corrected this description in the revised manuscript to ensure precise and consistent terminology throughout.*

Page 18: “automatically merging based on variance”. discussion on merging strategy is necessary. For example, a 10x variance is optimal? How many components in IFAM are appropriate? Merging strategy dependent on data?

Response: *Thank you for this valuable suggestion. We have revised the Discussion*

section to include a more thorough explanation of the merging strategy. Specifically, we justify the 10X variance by relating it to the order-of-magnitude effect size categories commonly used in BayesR. We also discuss that the appropriate number of variance components is data-dependent, with our results typically converging to a stable set of 2-5 components after merging, which enhances model robustness and computational efficiency.

Supplementary information

Page 2: “one random genetic effect (mu)”. Mu usually represents overall mean or intercept in a model, such as represented in the Bayesian model in this file.

Response: *Thank you for pointing out the issue. We corrected this mistake in the revised manuscript and used a unified symbol to represent the fixed effects and random effects throughout the text.*

Page 2: “b is a vector of the estimated effects of fixed covariates”. The description is not appropriate. b is the vector of fixed effects, not “estimated” and include effect for both categorical variables and continuous covariates.

Response: *Thank you for pointing out the issue. The original description of “b” was imprecise, and the manuscript has been revised.*

Page 2: “BayesR … using the WTCCC1 dataset”. Explain why not also using the UKB dataset.

Response: *Thank you for raising this point. We have included the results of the BayesR on the UKB dataset in the revised manuscript (Fig.3 and Supplementary Table 29).*

Page 3: Why the term of fixed effect is different from that in GBLUP model?

Response: *Thank you for pointing out the issue. We used a unified symbol to represent the fixed effects in the revised manuscript.*

Page 3: “where the MCMC chain length was set to 10,000, and the burn-in step was set to 4,000”. The total chain length and the samples for burn-in seem very small. For both BayesR and BayesRC, did you inspect convergence, mixing and effective sample size?

Response: *Thank you for pointing out the issue. In response, we conducted a thorough parameter testing, including the MCMC chain length and burn-in for both BayesR and BayesRC. For BayesR, we compared three configurations: 10,000/4,000, 50,000/20,000, and 100,000/40,000 based on the WTCCC1 dataset. The results (Supplementary Table 27) showed no significant difference in predictive accuracy, leading us to adopt the 50,000/20,000 configuration for smaller datasets and the 10,000/4,000 for larger datasets to save the cost and time of computation. Similarly, for BayesRC, comparisons between 50,000/20,000 and 100,000/40,000 revealed no significant performance difference (Supplementary Table 28), so the 50,000/20,000 configuration was used. We have updated the Supplementary Note with these details and the rationale for the chosen parameters.*

Supplementary Table 28. *Prediction performance of the different scenarios of BayesRC in the*

WTCCC1 dataset using the functional annotation from the RegulomeDB database. The "BayesRC" represents running the BayesRC using the default parameters, where the MCMC chain length (Numit) was set to 50,000, the burnin steps were set to 20,000, and the functional annotations are the same as the IFAM. The "BayesRC_opt1" represents running the BayesRC using the alternative parameters, where the MCMC chain length (Numit) was set to 100,000, the burnin steps were set to 40,000, and the functional annotations are the same as the IFAM. The "BayesRC_opt2" represents running the BayesRC using the alternative parameters, where the MCMC chain length (Numit) was set to 50,000, the burnin steps were set to 20,000, and the functional annotations are eQTL and matched TF motif.

Trait[1]	***BayesRC Numit=50K***	***BayesRC_opt1 Numit=100K***	***BayesRC_opt2 Two annotations***
BD	*0.5261(0.0043)*	*0.5261(0.0041)*	*0.5583(0.0042)* **
CAD	*0.5178(0.0042)*	*0.5189(0.0036)*	*0.5378(0.0048)* **
CD	*0.5371(0.0047)*	*0.5384(0.0046)*	*0.5821(0.0043)* **
HT	*0.5160(0.0031)*	*0.5166(0.0033)*	*0.5438(0.0037)* **
RA	*0.5291(0.0041)*	*0.5301(0.0040)*	*0.6581(0.0036)* **
T1D	*0.5510(0.0043)*	*0.5536(0.0044)* *	*0.7828(0.0024)* **
T2D	*0.5256(0.0036)*	*0.5247(0.0037)*	*0.5582(0.0037)* **
Mean	*0.5290*	*0.5298*	*0.6030*

[1] BD: bipolar disorder; CAD: coronary artery disease; CD: Crohn's disease; HT: hypertension; RA: rheumatoid arthritis; T1D: type 1 diabetes; T2D: type 2 diabetes;
** represents a significant difference ($P < 0.05$) in the prediction accuracy between the targeted scenario and BayesRC;*
*** represents an extremely significant difference ($P < 0.01$) in the prediction accuracy between the targeted scenario and BayesRC*

Page 3: "see the study of MacLeod et al". Provide the reference.

Response: *Thank you for pointing out this omission. The appropriate citation has been added to the revised supplementary.*

Page 5, Supplementary Fig.1: Need to point out that the results are based on WTCCC1 data. The colors should be consistent with those in Fig. 1 in the main manuscript. This figure does not provide more information beyond Fig.1 in the manuscript since the samples for different traits are almost identical.

Response: *Thank you for pointing out the issue. This Figure was removed in the revised supplementary. The detailed numerical results were presented in the Supplementary Table 7.*

Page 5, Supplementary Fig.2: Need to point out that the results are based on WTCCC1 data. Figure b does not provide more information beyond Figure a since the samples for different traits are almost identical.

Response: *Thank you for pointing out the issue. We have revised the supplementary material accordingly. The redundant panel (formerly Supplementary Fig.2b) has been removed, and the figure caption explicitly states that the results were based on the WTCCC1 dataset.*

Page 10: "from the IFAM model (no optimization of random effects)". Is it a regular multiple genetic component GBLUP model?

Response: *Thank you for pointing out the issue. Yes, this model is indeed a multiple random effects GBLUP model without the optimization of random effects (i.e., the merging of annotations), which is a specific scenario of the IFAM method. We have clarified this point in the revised supplementary.*

Page 18, Supplementary Table 7: Need to point out that the results are based on WTCCC1 data.

Response: *Thank you for this observation. We have revised the caption of Supplementary Table 7 (now Supplementary Table 14) to explicitly state that the results are based on the WTCCC1 dataset.*

Page 24-25, Supplementary Table 12 and 13: Provide SE of the estimates. What does "—" in Table 7 mean?

Response: *Thank you for pointing out the issue. We have added the standard error (SE) of the estimates to Supplementary Tables 12 and 13 (now Supplementary Tables 20 and 21). Furthermore, we have clarified in the footnote of the table that the symbol "--" denotes a missing value which is anomalous.*

Page 26, Supplementary Table 14: "using functional annotation information". Using also GWAS information?

Response: *Thank you for pointing out the issue. The functional annotation information included the trait-specific significant SNPs generated from GWAS. We have revised the caption for Supplementary Table 14 (now Supplementary Table 25) to accurately reflect the composition of the annotations.*

Page 27, Supplementary Table 15: Column IFAM is actually the same as IFAM_LDSC in Supplementary Table 14. The model name should be consistent across tables.

Response: *Thank you for identifying this inconsistency. We have revised this issue to ensure the model naming is consistent throughout and to eliminate any redundant presentation of information.*

Page 26-29, Supplementary Table 14-17: It is not 20-fold cross validation (10-fold cross validation). The SE in your case is an artificial product. You can repeat the cross validation in an unlimited number of times, for example 10,000 replicates, then the SE will be close to zero.

Response: *Thank you for pointing out the issue. We agree that the initial description of the cross-validation strategy was indeed confusing and that the reported standard errors (SEs) were a product of the repeated sampling rather than a measure of variability from a single k-fold partition. All the model evaluations and comparisons in our manuscript were conducted using a random splitting strategy, thus we have revised the manuscript to consistently refer to the approach as "repetitions" rather than "k-fold cross-validation".*

Page 31, Supplementary Table 19: The title is the same as Supplementary Table 12.

Response: *Thank you for identifying this error in the supplementary table titles. We have corrected them to accurately reflect their respective contents: the title for Supplementary Table 12 (now Supplementary Table 20) specifies it pertains to variance components, while the title for Supplementary Table 19 (now Supplementary Table 22) clarifies it reports estimates of heritability.*

Page 33, Supplementary Table 21: The table provides the same information as Supplementary Fig. 8.

Response: *Thank you for pointing out the issue. We have removed Supplementary Table 21 in the revised supplementary.*

Responses to Reviewer #4:

Remarks to the Author:
The study presents a thoughtful framework that leverages functional annotations to improve genomic prediction and tests robustness across annotation sources. However, gaps in clarity regarding design and description, as well as limited benchmarking, make it difficult to assess the generality and comparative advantage of the approach.

The method provides thoughtful attempt to leverage biological annotations for prediction, and effort to test robustness by using alternative or random annotation sets.

Response: *We sincerely thank you for your positive assessment and professional suggestions.*

1. The core modeling strategy, multiple random effects/annotation-partitioned variance components, is not new. Please clarify the incremental contribution beyond existing multi-component BLUP/Bayesian frameworks and related annotation-aware methods.

Response: *Thank you for pointing out the issue. In recent years, the exponential growth of omics data has driven a substantial increase in available functional annotation information. Traditional methods, such as the multiple random effects model, are often inadequate for effectively handling this vast number of functional annotations. While multi random effects model exist, IFAM provides a critical, data-adaptive layer that addresses a fundamental practical challenge: the instability of variance component estimation when numerous, potentially overlapping annotations are included. Our core innovation is the automated merging of annotations with similar variance contributions. This strategy, validated across diverse datasets, robustly prevents model degradation and ensures computational feasibility in scenarios where the non-auto-merged multi random effects model fails, particularly with large annotation sets (Fig.2~5).*
Furthermore, our benchmarking and control experiments demonstrated that IFAM's performance gain stems not merely from the inclusion of annotations, but from its ability to efficiently and stably integrate them to extract incremental value beyond the substantial contribution of GWAS hits alone. This represents a significant step towards omics-based prediction for complex traits. The advantages of IFAM were discussed in detail in the revised manuscript.

2. Although the paper emphasizes potential applications in both animals and plants, only one pig dataset with a relatively small sample size (a few thousand animals) is analyzed. In large populations with small effective population sizes (and therefore stronger LD), the benefits of using annotations may be minimal. A careful evaluation with larger datasets is needed.

Response: *Thank you for this important suggestion. We have included a new analysis on a large-scale Yorkshire pig population (N ≈ 16,000) in the revised manuscript (Fig.5). The results demonstrated that IFAM maintains robust performance in this dataset, achieving superior predictive accuracy and computational efficiency (completing analyses within approximately one hour) compared to other methods, while also exhibiting strong stability. Furthermore, to strengthen the cross-species evaluation, we*

have added an analysis by a rice dataset (Fig.6), further showcasing IFAM's applicability to agricultural traits. We believed these additions substantially strengthen our claims regarding the generalizability of IFAM.

3. For the human datasets, trait-specific significant SNPs from association analyses are used. Does this risk contaminating the testing dataset?

Response: *Thank you for pointing out the issue. We confirmed that all GWAS were conducted exclusively within the training set for each repetition. No information from the testing set was used in the discovery of trait-associated SNPs, thus strictly avoiding any contamination or bias in the performance evaluation. This procedure has been clarified in the revised Methods section.*

4. It appears that only distinct annotations are included in the analysis. Since many annotations are biologically overlapping, this could pose convergence issues. Was annotation overlap evaluated? The conclusion "It performed well for traits with both high heritability and low heritability, and it was also suitable for any type of functional annotations" seems overly broad.

Response: *Thank you for pointing out the issue. We agreed that the overlap among annotations is a key consideration. As suggested, we have evaluated the degree of overlap between the annotation sets used in our study, and the results confirmed substantial overlap between the annotations (Supplementary Fig.14~16). The strategy of merging annotations with similar variance contributions was designed to mitigate potential issues arising from this overlap in IFAM, thereby stabilizing variance component estimation and model performance. The original conclusion was indeed overly broad, and we have revised the statement to reflect our findings.*

5. The claim that "compared with Bayesian models that assume complex variances for markers, the IFAM model, which integrated biological prior knowledge, exhibited higher computational efficiency and predictive performance" requires further justification.

Response: *Thank you for requesting further justification for this claim. We acknowledged that the original statement was overly broad. IFAM does not universally surpass all Bayesian models, and it only utilizes the functional annotations to improve the accuracy of genomic prediction under the framework of BLUP. We have revised the relevant claim in the manuscript to reflect our findings accurately. Our comprehensive comparison across five datasets showed that IFAM achieved predictive accuracy comparable to BayesR on the WTCCC1 dataset and significantly outperformed BayesR on both pig datasets. The performance of BayesRC was unexpectedly poor, and potential reasons for this were explained in the revised Discussion section. Regarding computational efficiency, a direct comparison on the large-scale Yorkshire pig population confirmed that IFAM completed the analysis substantially faster than BayesR under identical computing conditions (Supplementary Table 36).*

6. For the UKB dataset, why was GBLUP the only benchmark? Could the differences observed simply reflect the use of annotations? Although the comparisons demonstrate that the proposed model outperforms GBLUP, which is essentially a special case of

their model, the lack of benchmarking against other advanced prediction methods weakens the persuasiveness of the results. For all benchmarks, are the reported accuracy improvements statistically significant?

Response: *Thank you for pointing out the issue. We agreed that the initial comparison was limited. In the revised manuscript, we have included comprehensive benchmarks on the UKB dataset against GBLUP, BayesR, and the non-auto-merged multiple random effects model (Fig.3 and Supplementary Tables 29 ~ 30). Running Adaptive MultiBLUP and BayesRC remained computationally infeasible at this scale. The new analyses confirmed that IFAM significantly outperforms GBLUP (by 6.84% on average) and the non-auto-merged multiple random effects model (by 43.25% on average), while achieving comparable accuracy to BayesR but with superior prediction bias and computing efficiency. This provided a much more robust and persuasive evaluation of IFAM's performance.*

We have performed a paired Student's t-test to evaluate the statistical significance of the accuracy improvements. These results have been added to the relevant tables.

7. One strength is the robustness test using random annotations. However, it remains unclear how much of the observed improvement is driven by GWAS SNPs versus other annotation sources. The paper repeatedly states that GWAS SNPs boost accuracy, but it does not present results using only GWAS SNPs as the annotation. Without that control, it is impossible to determine whether most of the gain comes solely from GWAS hits or whether other annotations provide meaningful additional value.

Response: *Thank you for pointing out the issue. As recommended, we conducted a new control experiment across all five datasets, where the model had only two random effects: one for the trait-specific significant SNPs from GWAS and another for all remaining SNPs. The results were provided in Supplementary Tables 25~26, 29~30, 31~32, 34~35, and 37~38. We found that the IFAM model, which integrates multiple functional annotations including the trait-specific significant SNPs, consistently matched or outperformed this control baseline. Specifically, compared to the accuracy of the control experiment, IFAM provided an average accuracy gain of 1.34% in the UKB, 0.09% in the WTCCC1, 0.01% in the Duroc pig, 0.08% in the Yorkshire pig, and a marginal reduction of 0.12% in the rice dataset. These results indicated that the performance gains from functional annotations appear more pronounced in larger populations (e.g., UKB dataset), where their correlation with causal variants can be more effectively leveraged. Moreover, the functional annotations for humans were generally more comprehensive and higher in quality relative to those for plants and animals, which likely contributes to IFAM achieving superior accuracy in human populations.*

In this study, in addition to species-specific functional annotation information, the SNPs associated with interesting traits were viewed as a type of trait-specific annotation, referring to the study of adaptive MultiBLUP (Speed and Balding) and BayesRC (MacLeod et al.), which utilized the QTLs or SNPs significantly associated with the trait to improve the accuracy of genomic prediction. Initially, we opted for easily accessible functional annotations as proxies for multi-omics data to avoid the complexities of omics data integration, given their general applicability across multiple

traits within a species. However, preliminary experiments revealed that we had overestimated the capacity of current functional annotations to explain the phenotypic variation of interesting traits, which represent only a fraction of the complex regulatory landscape underlying causal variants. Acknowledging this limitation, referring to other studies, we incorporated trait-specific significant association signals as an annotation category to enhance biological relevance. And the performance of IFAM improves with the inclusion of annotations more directly pertinent to the targeted trait. We anticipate that rapid advances in molecular biology will progressively yield more informative annotations, enabling better dissection of complex traits.

NCOMMS-25-55562A: Responses to Reviewers' Comments

Dear Reviewers,

Thank you for reviewing our manuscript (NCOMMS-25-55562A) entitled "*IFAM: Improving genomic prediction accuracy of complex traits by integrating massive types of functional annotation information*". We highly appreciate the time and effort that you have dedicated to providing your valuable feedback on our manuscript. We are grateful for your insightful comments and professional suggestions. We have incorporated changes to reflect the suggestions and have highlighted the changes within the manuscript. Below, we provide point-by-point responses to your comments and concerns; your comments are in black regular font, while our responses are in *blue italics*.

Responses to Reviewer #1:

Remarks to the Author:
The authors responded in a detailed way to the reviewer comments.

Response: *We sincerely thank you for your positive feedback.*

Responses to Reviewer #2:

Remarks to the Author:
The manuscript has been improved significantly. Comments from me and the co-reviewer have been considered in the revised manuscript. We have only one comment remaining.

To validate model performance, the whole dataset was randomly split into training set and validation set, which were repeated 10 (or 20) times to produce 10 (or 20) replicates. However, a standard paired t-test is inappropriate for comparing models based on these replicates. Because all replicates are derived from the same finite dataset, their validation sets overlap, making the replicates non-independent. This artificially inflates statistical significance as the number of replicates increases. Therefore, an alternative validation method or a corrected paired t test is required for the validation of model prediction ability.

Response: *We sincerely thank you for your positive assessment and professional suggestions. Using a standard paired t-test on repeated random splits from the same finite dataset violates the assumption of independence, potentially inflating statistical significance. Thank you for highlighting this critical point. To address this issue, we have now re-evaluated all statistical comparisons using the corrected resampled t-test, implemented via the 'correctR' R package. All relevant statistical results and their interpretation in the manuscript and supplementary materials have been updated accordingly.*

Responses to Reviewer #4:

Remarks to the Author:
My remaining concern is the updated analysis using two random effects (GWAS-significant SNPs vs. all remaining SNPs). In this setting, adding functional-annotation data provides minimal gains in livestock (+0.01%, +0.08%) and slightly lower accuracy in crops (−0.12%). As the authors note, this may be expected, and I appreciate the extensive benchmarking, however, I suggest stating this limitation clearly in the abstract.

Response: *We sincerely thank you for your positive assessment and professional suggestions. As noted, excluding trait-specific GWAS-derived annotations, functional annotations alone yielded a 1.40% predictive gain for UKB dataset but only marginal improvements in livestock and crop datasets. This suggests that the effectiveness of multi-omics information is constrained by species-specific annotation quality, tissue and trait relevance, and the limited resolution of existing functional datasets. As functional genomics resources continue to expand, their integration is expected to provide increasing benefits for genomic prediction. Following your professional suggestions, we have now explicitly addressed this limitation in the revised Abstract. In addition, we have expanded the Discussion section to discuss the potential factors that may contribute to the observed performance differences across species.*
